# Dnmt3a Is a Haploinsufficient Tumor Suppressor in CD8+ Peripheral T Cell Lymphoma

**DOI:** 10.1371/journal.pgen.1006334

**Published:** 2016-09-30

**Authors:** Staci L. Haney, G. Michael Upchurch, Jana Opavska, David Klinkebiel, Ryan A. Hlady, Sohini Roy, Samikshan Dutta, Kaustubh Datta, Rene Opavsky

**Affiliations:** 1 Department of Genetics, Cell Biology, and Anatomy, University of Nebraska Medical Center, Omaha, Nebraska, United States of America; 2 Eppley Institute for Research in Cancer and Allied Diseases, Fred and Pamela Buffett Cancer Center, University of Nebraska Medical Center, Omaha, Nebraska, United States of America; 3 Department of Biochemistry and Molecular Biology, University of Nebraska Medical Center, Omaha, Nebraska, United States of America; 4 Department of Molecular Pharmacology and Experimental Therapeutics, Mayo Clinic, Rochester, Minnesota, United States of America; 5 Center for Leukemia and Lymphoma Research, University of Nebraska Medical Center, Omaha, Nebraska, United States of America; University of Cambridge, UNITED KINGDOM

## Abstract

DNA methyltransferase 3A (DNMT3A) is an enzyme involved in DNA methylation that is frequently mutated in human hematologic malignancies. We have previously shown that inactivation of Dnmt3a in hematopoietic cells results in chronic lymphocytic leukemia in mice. Here we show that 12% of Dnmt3a-deficient mice develop CD8+ mature peripheral T cell lymphomas (PTCL) and 29% of mice are affected by both diseases. 10% of *Dnmt3a*^*+/-*^ mice develop lymphomas, suggesting that Dnmt3a is a haploinsufficient tumor suppressor in PTCL. DNA methylation was deregulated genome-wide with 10-fold more hypo- than hypermethylated promoters and enhancers, demonstrating that hypomethylation is a major event in the development of PTCL. Hypomethylated promoters were enriched for binding sites of transcription factors AML1, NF-κB and OCT1, implying the transcription factors potential involvement in Dnmt3a-associated methylation. Whereas 71 hypomethylated genes showed an increased expression in PTCL, only 3 hypermethylated genes were silenced, suggesting that cancer-specific hypomethylation has broader effects on the transcriptome of cancer cells than hypermethylation. Interestingly, transcriptomes of *Dnmt3a*^*+/-*^ and *Dnmt3a*^*Δ/Δ*^ lymphomas were largely conserved and significantly overlapped with those of human tumors. Importantly, we observed downregulation of tumor suppressor p53 in *Dnmt3a*^*+/-*^ and *Dnmt3a*^*Δ/Δ*^ lymphomas as well as in pre-tumor thymocytes from 9 months old but not 6 weeks old *Dnmt3a*^*+/-*^ tumor-free mice, suggesting that p53 downregulation is chronologically an intermediate event in tumorigenesis. Decrease in p53 is likely an important event in tumorigenesis because its overexpression inhibited proliferation in mouse PTCL cell lines, suggesting that low levels of p53 are important for tumor maintenance. Altogether, our data link the haploinsufficient tumor suppressor function of Dnmt3a in the prevention of mouse mature CD8+ PTCL indirectly to a *bona fide* tumor suppressor of T cell malignancies p53.

## Author Summary

Global deregulation of cytosine methylation is an epigenetic hallmark of hematologic malignancies that may promote tumorigenesis by silencing tumor suppressor genes, upregulating oncogenes, and inducing genomic instability. DNA methyltransferase 3a (DNMT3A) is one of the three catalytically active enzymes responsible for cytosine methylation and one of the most frequently mutated genes in myeloid and T cell malignancies. Its role in malignant hematopoiesis, however, remains poorly understood. Here we show that Dnmt3a is a haploinsufficient tumor suppressor in the prevention of peripheral T cell lymphomas in mice. Our molecular studies identified a large number of genes deregulated in the absence of Dnmt3a that may be putative drivers of oncogenesis. We also show that downregulation of the tumor suppressor p53 is an important event in the development of mouse T cell lymphomas. Thus, this study establishes a novel mouse model to elucidate how epigenetic deregulation of transcription contributes to the pathogenesis of T cell lymphomas.

## Introduction

DNA methylation is an epigenetic modification involved in transcriptional regulation of gene expression. Three catalytically active DNA methyltransferases—Dnmt1, Dnmt3a, and Dnmt3b—are involved in the generation and maintenance of DNA methylation in mammalian cells. Dnmt3a and Dnmt3b are classified as *de novo* enzymes due to their methylation activity during early embryogenesis [1], whereas Dnmt1 has a high affinity for hemi-methylated sites and functions in the maintenance of methylation marks during cellular division [2,3].

Recent studies suggest that all Dnmts may play roles in generating and maintaining DNA methylation. For instance, in mouse hematopoietic stem cells, Dnmt3a is responsible for maintaining DNA methylation in lowly methylated regions known as canyons [4]. In addition, Dnmt1 was shown to have cancer-specific *de novo* activity in a mouse model of MYC-induced T cell lymphomas [5], whereas Dnmt3a and Dnmt3b were primarily involved in maintenance methylation in tumors [6,7]. However, a deeper understanding of individual Dnmt’s activities in normal development and in cancer is still missing.

DNA methyltransferase 3a has emerged as a central regulator of hematopoiesis over the last several years. The interest in Dnmt3a was in particular fueled by recent findings of somatic mutations in human hematologic malignancies of myeloid and T cell origin [8,9]. Given the importance of DNA methylation for differentiation of hematopoietic lineages [10] along with critical roles of Dnmt3a in differentiation and self-renewal of hematopoietic stem cells [11,12], it is not unexpected that a disruption of Dnmt3a activity affects a variety of cell types and has the potential to transform hematopoietic lineages. For example, recent studies using the *Mx1-Cre* transgene to conditionally delete Dnmt3a in hematopoietic stem and progenitor cells (HSPCs) followed by transplantation into lethally irradiated recipients showed that a vast majority of mice develop myeloid disorders such as myeloid dysplastic syndrome and acute myeloid leukemia (69%) with rare occurrences of CD4+CD8+ double positive T-ALL (<12%) or B-ALL (<4%) [13]. In addition, both myeloid deficiencies and neoplasms were observed in mice transplanted with Dnmt3a-null bone marrow obtained from *Mx1-Cre;Dnmt3a*^*fl/fl*^ mice, altogether highlighting the importance of Dnmt3a in prevention of myeloid transformation [14,15]. However, the role of Dnmt3a in differentiation into hematopoietic lineages and molecular functions in normal and malignant hematopoiesis in particular remain poorly understood.

To elucidate the role of Dnmt3a in normal and malignant hematopoiesis we used the *EμSRα-tTA;Teto-Cre;Dnmt3a*^*fl/fl*^*;Rosa26LOXP*^*EGFP/EGFP*^ (*Dnmt3a*^*Δ/Δ*^) mouse model to conditionally delete Dnmt3a in all cells of the hematopoietic compartment. Using this model, we previously showed that long-term Dnmt3a-defficiency resulted in the development of a chronic lymphocytic leukemia (CLL) around 1 year of age [16, 17]. In addition, we previously reported that combined inactivation of Dnmt3a and Dnmt3b results in the development of CLL and peripheral T cell lymphoma (PTCL) [16], however the molecular basis of PTCL is poorly understood. Here we expanded on our previous studies by observation of a larger cohort of *Dnmt3a*^*Δ/Δ*^ mice. These studies revealed that while ~60% of mice succumb to CLL, ~40% of mice develop CD8+ mature peripheral T cell lymphoma either in combination with CLL or as a singular disease. Furthermore, we found that loss of one allele of Dnmt3a is sufficient to induce CD8+ PTCL in 10% of Dnmt3a heterozygous mice with tumors retaining expression of the *wild-type* allele. Molecular profiling of methylation and gene expression identified promoter hypomethylation as a major event in tumorigenesis of PTCL, which was frequently accompanied by upregulation of gene expression. Furthermore, we identified downregulation of tumor suppressor p53 not only in *Dnmt3a*^*+/-*^ and *Dnmt3a*^*Δ/Δ*^ lymphomas but also in pre-tumor thymocytes, suggesting that p53 downregulation is likely relevant in the initiation/progression of lymphomagenesis.

Altogether, our data demonstrate that Dnmt3a is a haploinsufficient tumor suppressor in the prevention of CD8+ T cell transformation and highlight the importance of understanding of the roles of Dnmt3a target genes in disease pathogenesis.

## Results

### *Dnmt3a*^*Δ/Δ*^ mice develop B220+CD19+CD5+ CLL and CD8+ PTCL

**Fig 1:**
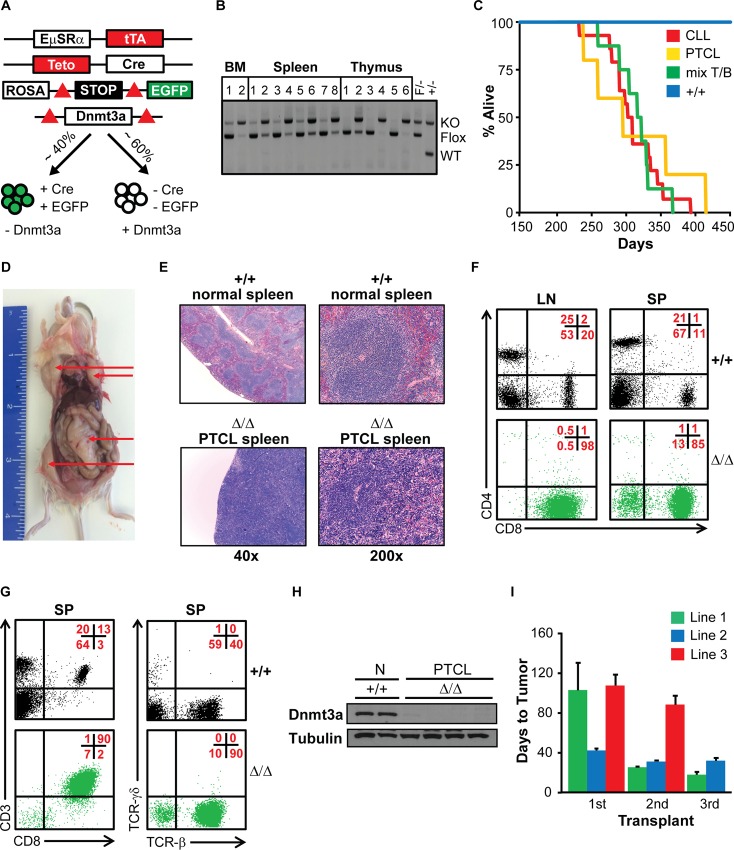
*Dnmt3a*^*Δ/Δ*^ mice develop B220+CD19+CD5+ CLL and CD8+ PTCL. (A) Genetic setting used to conditionally delete Dnmt3a. The tetracycline activator protein (tTA) is expressed in ~40% of all hematopoietic cells (including stem cells). tTA promotes expression of the *Teto-Cre* transgene. Expression of Cre results in the excision of the stop cassette located upstream of the *Rosa26LOXP*^*EGFP*^ reporter locus and deletion of Dnmt3a within the same subpopulation of cells. Thus, inclusion of the EGFP transgene allows for monitoring cells expressing tTA, and Cre, as well as to identify cells deleted for Dnmt3a. (B) PCR-based genotyping of the Dnmt3a locus using DNA isolated from FACS-sorted EGFP-positive (+) and EGFP-negative (−) populations on cells obtained from the bone marrow (BM), spleen (SP) and thymus (TH) of 6-week-old *Dnmt3a*^*−/−*^ mice. PCR reactions were performed on samples in the following order, BM: (1) -LSK, (2) +LSK, SP: (1) -B2, (2) +B2, (3) -B1, (4) +B1, (5) -myeloid, (6) +myeloid, (7) -erythroid, (8) +erythroid, TH: (1) -DP, (2) +DP, (3) -CD4, (4) +CD4, (5) -CD8, (6) +CD8. Immunophenotypes of sorted populations are as follows: LSK (Lineage-, Sca-1+, ckit+), B2 (B220+, CD5-), B1 (B220+, CD5+), Myeloid (CD11b+), erythroid (Ter119+), DP (CD4+, CD8+), CD4 (CD4+, CD8-), CD8 (CD4-, CD8+). Fragments from floxed (F), *wild-type* (WT) and knockout (KO) alleles are shown. DNA from conventional Dnmt3a heterozygous (F/- and +/-) mice were used as controls. (C) Kaplan Meyer survival curve for *Dnmt3a*^*Δ/Δ*^ mice. Moribund mice were classified at the time of terminal harvest with CLL (red), PTCL (yellow), or mixed CLL/PTCL (green) based on the presence of B-1a or CD8+ T cells tumors, as determined by FACS. *Dnmt3a*^*+/+*^ *wild-type* (+/+) controls are shown in blue. (D) A terminally ill *Dnmt3a*^*Δ/Δ*^ mouse with PTCL. Generalized lymphadenopathy is denoted by arrows. (E) H&E stained sections of *Dnmt3a*^*+/+*^ (normal) and *Dnmt3a*^*Δ/Δ*^ (PTCL) spleens (40X and 200X). (F) CD8 and CD4 expression in cells isolated from the lymph nodes (LN) and spleens (SP) of *Dnmt3a*^*+/+*^ control (+/+) and *Dnmt3a*^*Δ/Δ*^ PTCL (Δ/Δ) mice as determined by FACS. Percentages of cells in each quadrant are shown in the top right in red. (G) CD3, CD8, TCR-β and TCR-γδ expression in cell isolated from the spleens (SP) of *Dnmt3a*^*+/+*^ control (+/+) and *Dnmt3a*^*Δ/Δ*^ PTCL (Δ/Δ) mice, as determined by FACS. (H) Immunoblot showing Dnmt3a protein levels in *Dnmt3a*^*+/+*^ normal (N) controls (+/+) and *Dnmt3a*^*Δ/Δ*^ PTCL (Δ/Δ) lymph node samples. γ-tubulin is shown as a loading control. (I) Time to tumor for primary (1), secondary (2) and tertiary (3) sub-lethally irradiated FVB-recipient mice serially transplanted with CD8+ PTCL tumors isolated from the lymph nodes of *Dnmt3a* ^*Δ/Δ*^ terminally sick mice. Data are presented as average time to tumor development. Three PTCL lines are shown.

We previously utilized quadruple transgenic mice, *EμSRα-tTA;Teto-Cre;Dnmt3a*^*fl/fl*^*; Rosa26LOXP*^*EGFP/EGFP*^ (designated hereafter as *Dnmt3a*^*Δ/Δ*^), to conditionally inactivate Dnmt3a in HSPCs as well as mature cells of all hematopoietic lineages (**Fig 1A**). In such genetic setting, Dnmt3a is deleted in only ~40% of hematopoietic cells due to restricted patterns of *EμSRα-tTA* expression and cells are marked by EGFP expression driven from a reporter gene [16,17]. In the remaining 60% of hematopoietic cells the *EμSRα-tTA* transgene is not expressed and, as a result, the Dnmt3a conditional allele is not deleted and the EGFP reporter is not expressed. PCR based genotyping confirmed Dnmt3a deletion in EGFP–positive but not EGFP-negative stem cells as well as T-, B-, and myeloid cells (**Fig 1B**). Furthermore, Dnmt3a deletion was specific to cells of the hematopoietic lineages and was not observed in solid tissues (S1A **Fig**). Previously, we observed a small cohort of *Dnmt3a*^*Δ/Δ*^ mice and reported the development of a CLL-like disease with a median survival of 371 days [16]. Here we utilized a larger cohort of 42 *Dnmt3a*^*Δ/Δ*^ mice to observe the phenotypic consequences of Dnmt3a inactivation. Consistent with our previously reported data, we observed the development of a CLL-like disease in 61% of mice with a median survival of 306 days (**Fig 1C and S1B Fig**). This disease was characterized by expansion of B220+CD19+CD5+IgM+ EGFP+ cells in the spleen, blood, bone marrow, peritoneal cavity (IP) and occasionally in the lymph nodes (**S1C and S1D Fig**). Interestingly, 12% of *Dnmt3a*^*Δ/Δ*^ mice developed a different disease (MS = 295 days) characterized not only by splenomegaly but also by a significant enlargement of lymph nodes that was not observed in the CLL cases (**Fig 1C, 1D and S1B Fig**). Histological analysis of spleens showed near complete effacement of the red pulp by massively expanded white pulp (**Fig 1E**). Small- to medium-sized cells were EGFP+, expressed markers of mature T cells–CD3, CD5, TCRβ and CD8 –and were negative for the expression of CD4, TCRγδ, NK-1.1 and CD16 (**Fig 1F, 1G and S1E Fig**). To determine if *Dnmt3a*^*Δ/Δ*^ PCTLs are clonal, we analyzed TCR-Vβ rearrangement by FACS analysis. All three *Dnmt3a*^*Δ/Δ*^ lymphomas analyzed showed only one or two TCR-Vβ rearrangements, suggesting that they were clonally derived from a single cell following somatic recombination of the TCR-β locus (**S1F Fig and S1 Table**). These phenotypes are most consistent with those observed in human cytotoxic peripheral T cell lymphomas not otherwise specified (PTCL-NOS).

Dnmt3a was efficiently deleted in lymph node cells of terminally ill mice as determined by immunoblot analysis using anti-Dnmt3a antibody (**Fig 1H**). When lymph node cells from terminally sick *Dnmt3a*^*Δ/Δ*^ mice were injected into the IP cavity of sublethally irradiated FVB mice, recipients developed a CD3+CD8+ PTCL similar to that observed in the donor mice (**Fig 1I**), suggesting that *Dnmt3a*^*Δ/Δ*^ cells have tumorigenic potential. Tumor burden (scored as average weights of spleens in terminally ill mice) was on average higher in PTCL than in CLL (**S1G Fig**).

In addition to the development of distinct disease types in individual mice, in 29% of mice we observed the simultaneous development of CLL and PTCL with a median survival of 293 days (**Fig 1C, S1B and S1H Fig**). Interestingly, all CLL and PTCL cases presented with B220+CD19+CD5+ and CD3+CD8+ immunophenotypes, respectively, suggesting that normal B-1a B cells and cytotoxic CD8+ T cells are in particularly sensitive to cellular transformation in the absence of Dnmt3a. Altogether, these data suggest that Dnmt3a is a tumor suppressor gene in prevention of CLL and PTCL in mice.

### Dnmt3a is a haploinsufficient tumor suppressor in the prevention of CD8+ PTCL in mice

**Fig 2:**
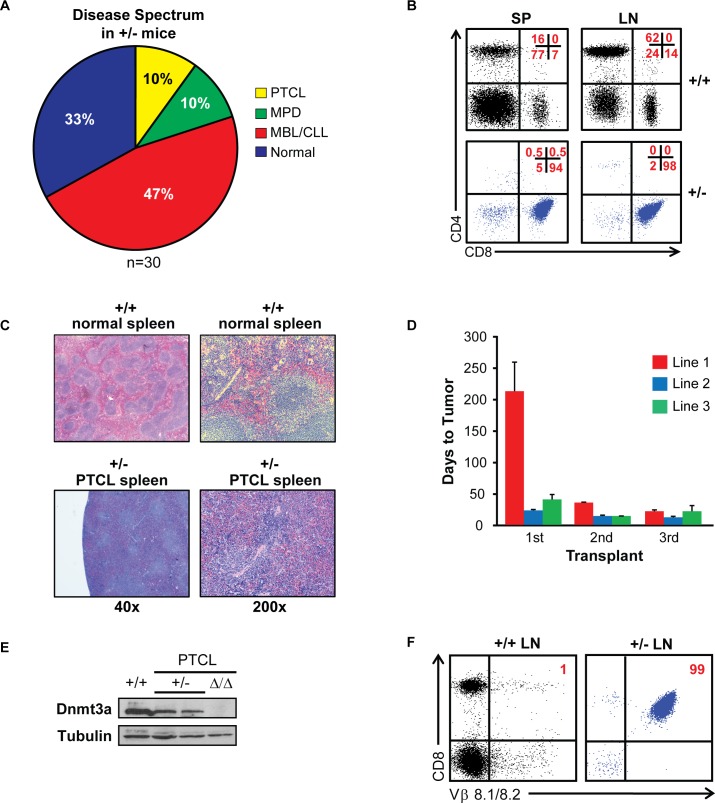
Dnmt3a is a haploinsufficient tumor suppressor in the prevention of CD8+ PTCL in mice. (A) Percentage of conventional heterozygous *Dnmt3a*^*+/-*^ mice developing PTCL (yellow), MPD (green), CLL (red), or no disease (blue) at time of harvest, as determined FACS. (N = 30). (B) FACS analysis of CD8 and CD4 expression in cells isolated from the spleens (SP) and lymph nodes (LN) of *Dnmt3a*^*+/+*^ control (+/+) and *Dnmt3a*^*+/-*^ PTCL (+/-) mice. Percentages of cells in each quadrant are shown in the top right in red. (C) H&E stained sections of *Dnmt3a*^*+/+*^ (normal) and *Dnmt3a*^*+/-*^ (PTCL) spleens (40X and 200X). (D) The time to tumor development for primary (1), secondary (2) and tertiary (3) sub-lethally irradiated FVB-recipient mice serially transplanted with CD8+ PTCL tumors isolated from the lymph nodes of a *Dnmt3a*^*+/-*^ terminally sick mice. Data are presented as average time to tumor development. Three PTCL lines are shown. (E) Immunoblot showing Dnmt3a protein levels in *Dnmt3a*^*+/+*^ controls (+/+) and *Dnmt3a*^*+/-*^ PTCL (+/-) samples. *Dnmt3a*^*Δ/Δ*^ PTCL (Δ/Δ) is shown as a negative control. γ-tubulin is shown as a loading control. (F) Representative flow diagram showing CD8 and TCR-Vβ 8.1/8.2 expression in *Dnmt3a*^*+/+*^ controls (+/+) and *Dnmt3a*^*+/-*^ PTCL LN samples.

We have recently reported that mice harboring a conventional knockout allele of Dnmt3a (*Dnmt3a*^*+/-*^ mice) develop either CLL, myeloproliferative disorder or remain healthy by 16 months of age [17]. Here we expanded these studies by observing a larger cohort of 30 *Dnmt3a*^*+/-*^ and 20 control *Dnmt3a*^*+/+*^ mice. Interestingly, we found that 3 out of 30 analyzed mice developed CD8+ T cell lymphomas, which were indistinguishable from those observed in *Dnmt3a*^*Δ/Δ*^ mice (**Fig 2A–2C**). None of the control mice were affected by lymphoma and remained healthy during the observational period. Serial transplantation of *Dnmt3a*^*+/-*^ lymphoma cells induced PTCL within 2 months in secondary and tertiary transplanted mice, illustrating their selective advantage to grow and induce disease (**Fig 2D**). *Dnmt3a*^*+/-*^ lymphomas retained approximately 50% expression of Dnmt3a, suggesting that the remaining allele is expressed in fully transformed cells (**Fig 2E).** Like *Dnmt3a*^*Δ/Δ*^ lymphomas, *Dnmt3a*^*+/-*^ lymphomas were also clonal (**Fig 2F and S1 Table**). Importantly, sequencing analysis of cDNA generated from two independent *Dnmt3a*^*+/-*^ PTCL samples revealed no mutations in the coding sequence of Dnmt3a (**S1 File**), demonstrating that the expressed Dnmt3a allele is in the *wild-type* configuration. Similarly, we did not find any mutations in the coding sequences of two genes that are commonly mutated in human T cell malignancies, Tet2 and RhoA, and their expression was not changed in Dnmt3a-deffcient lymphomas, suggesting that changes in the activity of these genes may not be involved in the transformation of T cells in this model (**S2 File and S2 Fig**). Altogether, these data suggest that Dnmt3a is a haploinsufficient tumor suppressor gene in the prevention of CD8+ T cell lymphomas and CLL in mice.

### A majority of promoters are methylated and inactive in normal mouse CD8+ T cells

**Fig 3:**
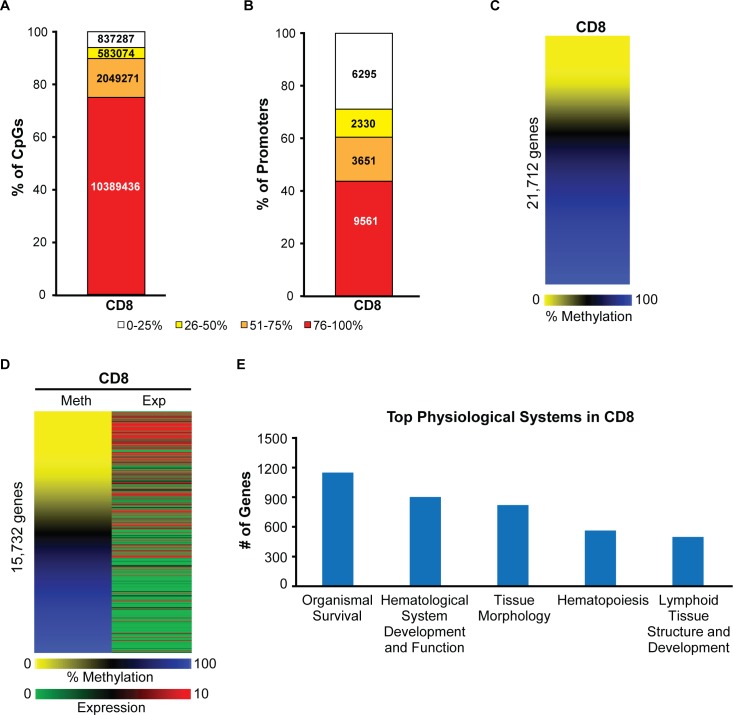
A majority of promoters are methylated and inactive in normal mouse CD8+ T cells. (A) CpG methylation in *wild-type* CD8+ cells, as determined by WGBS. Individual CpGs were placed into quartiles based on percent methylation (0–25%, 26–50%, 51–75%, and 76–100%). (B) Percent methylation shown by quartiles for core promoter regions (-300bp to +150bp relative to the TSS) in *wild-type* CD8+ cells. Methylation percentages for all CpGs across the 450bp region were averaged to give a mean methylation value for each gene’s core promoter. (C) A heat map displaying methylation status of 21,712 promoters in *wild-type* CD8+ as determined by WGBS. Methylation percentage for individual CpGs were annotated to the promoter regions −300bp to +150bp relative to the transcription start site (TSS). Methylation percentages for all CpGs across the 450bp region were averaged to give a mean methylation value for each gene promoter. Lowly methylated promoters are shown in yellow and highly methylated promoters in blue (D) Heat map presentation of gene-matched promoter methylation (as analyzed in panel C) and corresponding transcriptional expression (averaged FPKM values) in *wild-type* CD8+ cells, as determined by WGBS and RNA-seq for 15,732 genes. Highly expressed genes are denoted in red and lowly expressed genes are denoted in green. (E) Ingenuity Pathway analysis (IPA) of highly expressed genes (FPKM ≥ 10) in *wild-type* CD8+ cells. The top subcategories obtained in Physiological System, Development and Functions are displayed (P<0.05, for all subcategories).

To determine the nature of deregulated molecular events during PTCL development in *Dnmt3a*^*Δ/Δ*^ mice, we performed global methylation analysis using whole genome bisulfite sequencing (WGBS) and gene expression profiling by RNA-seq on CD8+ T cells isolated from *Dnmt3a*^*+/+*^ spleens, as this cellular population is immunophenotypically the closest normal counterpart of CD8+CD4- PTCLs. Methylation analysis revealed that 75% of 13,859,068 CpG dinucleotides were heavily methylated (≥76%), while only 6% were methylated at low levels (≤25%) (**Fig 3A**). The remaining 19% of CpG were methylated at intermediate levels (25% to 75%). Likewise, 44% of core promoters (-300 to +150 bp relative to transcription start site; TSS) were heavily methylated (≥76%), while 29% were lowly methylated (≤25%) (**Fig 3B**). Analysis of methylation across core promoter regions revealed that over 13,000 genes had a mean methylation value greater than 50%, suggesting that the majority of promoters in CD8+ T cells are heavily methylated (**Fig 3C and S2 Table**). A combined gene expression and methylation analysis revealed that the majority of genes with low levels of promoter methylation were expressed, whereas genes with high levels of promoter methylation were largely repressed, suggesting that promoter methylation correlates with gene expression (**Fig 3D and S3 Table**).

Ingenuity pathway analysis (IPA) of highly expressed genes in CD8+ T cells revealed the top subcategories of genes significantly associated with *organismal survival*, *hematological system*, *tissue morphology*, *hematopoiesis*, *lymphoid tissue structure* (**Fig 3E**), underlining their link to hematopoietic system. Altogether, these data reveal that a significant number of promoters are hypermethylated and inactive in normal CD8+ T cells, highlighting both the importance of DNA methylation in differentiation and potential for deregulation of these genes upon inactivation of DNA methyltransferases.

### *Dnmt3a*^*Δ/Δ*^ PTCL is characterized by genome wide hypomethylation

**Fig 4:**
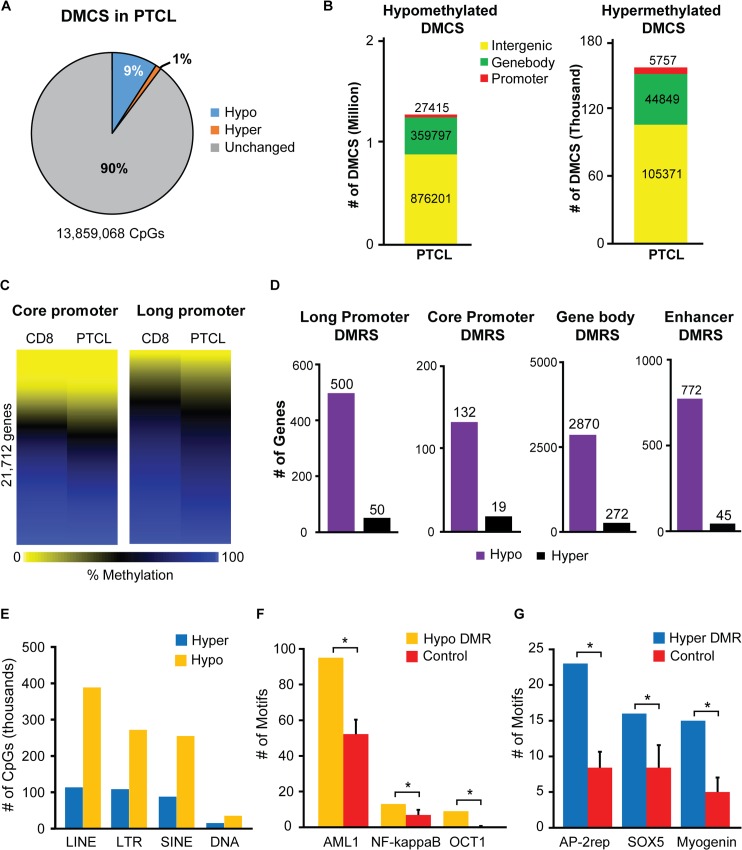
*Dnmt3a*^*Δ/Δ*^ PTCL is characterized by genome wide hypomethylation. (A) Percentage of differentially methylated CpGs (DMCS) in *Dnmt3a*^*Δ/Δ*^ PTCL relative to *wild-type* CD8+ cells. Differentially methylated CpGs (DMCS) are defined as either hypo- (blue) or hypermethylated (orange) by a ≥30% change in percent CpG methylation in tumor samples compared to *wild-type* control samples. CpGs not meeting these criteria are shown in gray (unchanged). (B) Genomic location of hypomethylated (left) and hypermethylated (right) DMCS in *Dnmt3a*^*Δ/Δ*^ PTCL, as compared to *wild-type* CD8+ cells. DMCS were annotated to long gene promoters (-1500 to +500bp relative to TSS), gene bodies, or intergenic regions. (C) Heat map comparing the methylation status of 21,712 promoters in *wild-type* CD8+ and *Dnmt3a*^*Δ/Δ*^ PTCL samples, as determined by WGBS. The averaged percent CpG methylation at core promoter regions (-300bp to +150bp relative to the TSS, *left*) and at long promoter regions (-1500bp to +500bp relative to the TSS, *right*) are displayed. (D) The number of genes with differentially methylated regions (DMRS) in their long promoters, core promoters, gene bodies and predicted enhancers in *Dnmt3a*^*Δ/Δ*^ PTCL relative to CD8+ control. (E) The number of hypo- and hypermethylated CpGs present in LINE, LTR, SINE, and DNA repeat elements in *Dnmt3a*^*Δ/Δ*^ PTCL, as compared to *wild-type* CD8+ cells. (F) The number of AML1, NF-κB, and OCT1 transcription factor motifs present in hypomethylated promoters in *Dnmt3a*^*Δ/Δ*^ PTCL, as compared to *wild-type* CD8+ cells (yellow). The average motif count of 12 randomly generated control promoter sets is shown in red, with error bars denoting standard deviation. (*) denotes p<0.05 by a Wilcoxon rank test. (G) The number of AP-2rep, SOX5, and myogenin transcription factor motifs present in hypermethylated promoters in *Dnmt3a*^*Δ/Δ*^ PTCL, as compared to *wild-type* CD8+ cells (blue). The average motif count of 12 randomly generated control promoter sets is shown in red, with error bars denoting standard deviation. (*) denotes p<0.05 by a Wilcoxon rank test.

To determine the effects of Dnmt3a loss on the cancer methylome, we next performed WGBS on DNA isolated from *Dnmt3a*^*Δ/Δ*^ PTCL cells. Out of ~14 million CpG dinucleotides analyzed, we observed decreased methylation in 1,263,413 (9%) CpGs and increased methylation in 155,977 (1%) CpGs (**Fig 4A and S4 Table)**. By analysis of differentially methylated cytosines (DMCs) we found that the majority of DMCs were present in gene bodies and intergenic regions, where hypomethylation was 8 fold higher than hypermethylation (**Fig 4B and S4 Table**). Although the vast majority of changes in methylcytosine levels occurred in gene bodies and intergenic regions, we detected an overall decrease in methylation in both long and short promoter regions (-1500 to +500 bp and -300 to +150 bp relative to TSS, respectively) in *Dnmt3a*^*Δ/Δ*^ PTCL relative to CD8+ T cell controls (**Fig 4C and S5 Table**). Likewise, analysis of differentially methylated regions (DMRS) found significant changes in the methylation of long promoters, with 500 hypomethylated DMRS and 50 hypermethylated DMRS identified in PTCL relative to CD8+ T cell controls (**Fig 4D and S6 Table**). Similarly, short promoters were hypomethylated (132) more so than hypermethylated (19) in PTCL (**Fig 4D and S6 Table**). Like with promoters, hypomethylated DMRS were 10-and 18-fold higher than hypermethylated DMRS in gene bodies and enhancers, respectively (**Fig 4D and S6 Table**). Extensive hypomethylation was also observed in repeat elements, with LINE elements showing the largest degree of hypomethylation (~4 fold) compared to hypermethyation (**Fig 4E**).

Next, we were interested in determining if differentially methylated promoter regions shared particular transcription factor binding motifs. This analysis revealed a significant enrichment for AML1, NF-κB, and OCT1 binding motifs in hypomethylated promoters (**Fig 4F**). This suggests a possible involvement of these factors in maintenance methylation performed by Dnmt3a. Similarly, AP-2rep, SOX5, and myogenin binding motifs were enriched in gene promoters hypermethylated in tumors, possibly implying role of these factors in cancer-specific aberrant methylation (**Fig 4G**). Further functional analysis will be required to test an involvement of these proteins in deregulated methylation in mouse lymphomas.

### Promoter and gene-body hypomethylation is present throughout the genome of *Dnmt3a*^*Δ/Δ*^ PTCLs

**Fig 5:**
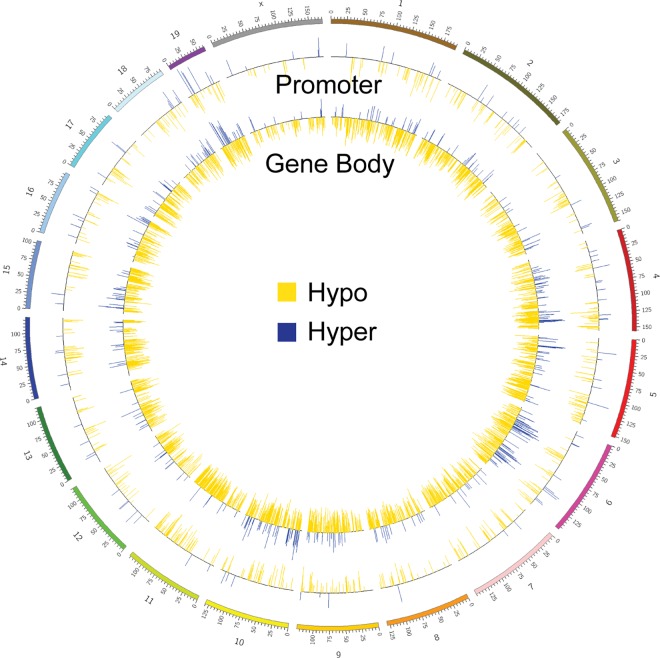
Promoter and gene-body hypomethylation is present throughout the genome of *Dnmt3a*^*Δ/Δ*^ PTCLs. Circos plot of DMRS annotated to promoters and gene bodies in *Dnmt3a*^*Δ/Δ*^ PTCL relative to *wild-type* CD8+ cells. DMRs aligning to promoter (outer circle) and gene body (inner circles) are displayed in relation to its chromosomal position in the mouse genome. Hypomethylated DMRS are indicated by yellow lines and hypermethylated DMRS are indicated by blue lines.

Locus-specific analysis revealed that hypo- and hypermethylated DMRS associated with promoters and gene bodies were relatively equally distributed across the genome, with the highest number of hypomethylated promoters present on chromosomes 11 and 5, lowest numbers on chromosomes X and 12 (**Fig 5 and S6 Table**). Interestingly, very few differentially methylated promoters were detected on the X chromosome, suggesting that Dnmt3a is dispensable for maintenance methylation in these areas of the genome (**Fig 5 and S6 Table**). Altogether, our data suggest that disease development in the absence of Dnmt3a results in decreased methylation across the genome with a significant number of gene promoters affected whose untimely activation may contribute to the malignant transformation of CD8+ T cells.

### Promoter hypomethylation is conserved across multiple *Dnmt3a*^*Δ/Δ*^ and *Dnmt3a*^*+/-*^ mouse lymphomas

To determine whether the methylation landscape generated by WGBS is specific to the PTCL sample profiled or rather represents common changes that occur in Dnmt3a-deficient lymphoma, we validated hypo- and hypermethylated promoters using reduced representation bisulfite sequencing (RRBS) on additional normal CD8+ T cells and *Dnmt3a*^*Δ/Δ*^ T cell lymphomas. This analysis confirmed hypomethylation of 90 gene promoters identified by WGBS in *Dnmt3a*^*Δ/Δ*^ PTCL sample (**S3A Fig, S3B Fig and S7 Table**). In addition, 31 out of 38 gene promoters were confirmed to be hypermethylated by RRBS (**S3A Fig, S3B Fig and S7 Table**). The lesser extent to which hypomethylated promoters were confirmed by RRBS is not surprising in view of the inherent bias of the RRBS method which tends to underestimate the number of hypomethylated events in promoters with low CG content [18]. In fact, analysis of CpG content across DMRS revealed that hypomethylated promoters represent regions of lower CpG content when compared to hypermethylated promoters (**S3C Fig**). Altogether, these data are in good agreement with results obtained from WGBS, which show large scale promoter hypomethylation in Dnmt3a-defficient PTCLs.

**Fig 6:**
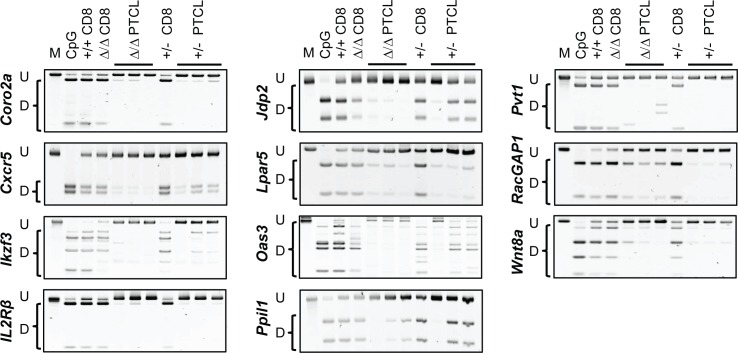
Promoter hypomethylation is conserved across multiple *Dnmt3a*^*Δ/Δ*^ and *Dnmt3a*^*+/-*^ mouse lymphomas. COBRA analysis of promoter methylation for *Coro2a*, *Cxcr5*, *Ikzf3*, *IL2Rβ*, *Jdp2*, *Lpar5*, *Oas3*, *Ppil1*, *Pvt1*, *Racgap1* and *Wnt8a* in *wild-type* CD8+, *Dnmt3a*^*Δ/Δ*^ pre-tumor CD8+, *Dnmt3a*^*Δ/Δ*^ PTCL, *Dnmt3a*^*+/-*^ pre-tumor CD8+, and *Dnmt3a*^*+/-*^ PTCL samples. PCR fragments amplified from bisulfite-treated genomic DNA were digested with the restriction enzymes *BstU*I, *Taq*1 or *Tai*I. Undigested (U) and digested (D) fragments correspond to unmethylated and methylated DNA, respectively. Control PCR fragments generated from fully methylated mouse genomic DNA that is undigested (M) or digested (CpG) are shown.

To further validate data obtained by global methods on *Dnmt3a*^*Δ/Δ*^ PTCL and to assess if methylation patterns are conserved in *Dnmt3a*^*+/-*^ PTCL, we performed locus-specific methylation analysis using Combined Bisulfite Restriction Analysis (COBRA) for 11 selected genes in multiple independent tumor samples from *Dnmt3a*^*Δ/Δ*^ and *Dnmt3a*^*+/-*^ mice. Consistently with results obtained by WGBS, promoters of *Coro2a*, *Cxcr5*, *Ikzf3*, *Il2Rβ*, *Jdp2*, *Lpar5*, *Oas3*, *Ppil1*, *Pvt1*, *RacGAP1*, *and Wnt8a* were found to be hypermethylated in normal CD8+ T cells but hypomethylated in three independent *Dnmt3a*^*Δ/Δ*^ PTCL samples (**Fig 6**). Furthermore, all 11 promoters were hypomethylated in *Dnmt3a*^*+/-*^ PTCL samples, suggesting that loss of a single Dnmt3a allele is sufficient to induce patterns of promoter hypomethylation similar to those observed in *Dnmt3a*^*Δ/Δ*^ PTCL samples (**Fig 6**). To determine if promoter hypomethylation observed in *Dnmt3a*^*Δ/Δ*^ and *Dnmt3a*^*+/-*^ PTCL occurs as a result of Dnmt3a inactivation in normal CD8+ T cells due to the lack of Dnmt3a’s *de novo* or maintenance activity, we analyzed promoter methylation in CD8+ T cells isolated from 8-week old *Dnmt3a*^*Δ/Δ*^ and *Dnmt3a*^*+/-*^ mice. For all 11 genes tested, promoters were hypermethylated in *Dnmt3a*^*Δ/Δ*^ and *Dnmt3a*^*+/-*^ CD8+ T cells to a similar degree as in *Dnmt3a*^*+/+*^ CD8+ T cells, suggesting that partial or complete inactivation of Dnmt3a does not affect the methylation status of these genes in normal CD8+ T cells during development (**Fig 6**). Altogether, our data demonstrate that changes in promoter methylation identified using WGBS likely represent tumor-specific events occurring in mouse PTCL driven by mono or bi-allelic loss of Dnmt3a.

### Gene expression is deregulated in *Dnmt3a*^*Δ/Δ*^ PTCL

**Fig 7:**
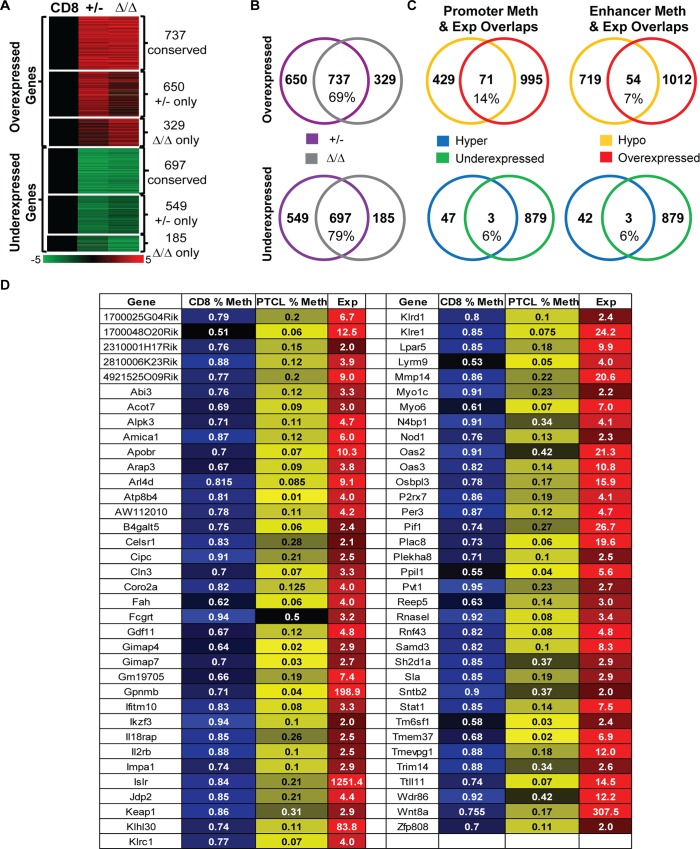
Gene expression is deregulated in *Dnmt3a*^*Δ/Δ*^ PTCL. (A) Heat map of RNA-seq global expression data displaying differentially expressed genes in *Dnmt3a*^*+/-*^ and *Dnmt3a*^*Δ/Δ*^ PTCL (≥2 fold change and a p-value < 0.05) relative to *wild-type* CD8+ cells, 737 genes were overexpressed and 697 genes were underexpressed in both *Dnmt3a*^*+/-*^ and *Dnmt3a*^*Δ/Δ*^ PTCL. 650 overexpressed and 549 underexpressed genes were specific to *Dnmt3a*^*+/-*^ PTCL, whereas 329 overexpressed and 185 underexpressed genes were only observed in *Dnmt3a*^*Δ/Δ*^ PTCL. A color bar displays fold change in gene expression with overexpressed shown in red and underexpressed in green. (B) Venn diagram showing overlap in over- and underexpressed genes between *Dnmt3a*^*+/-*^ PTCL (purple) and *Dnmt3a*^*Δ/Δ*^ PTCL (gray). (C) (left) Venn diagram showing overlap between the number of differentially expressed genes (red = overexpression; green = underexpression) and the number of differentially methylated gene promoters (yellow = hypomethylation; blue = hypermethylation) in *Dnmt3a*^*Δ/Δ*^ PTCL relative to *wild-type* CD8+ controls. (right) Overlap between differentially expressed genes and differentially methylated enhancers regions. (D) List of genes hypomethylated and overexpressed in *Dnmt3a*^*Δ/Δ*^ PTCL (HOT genes). Promoter methylation for *wild-type* CD8+ cells and *Dnmt3a*^*Δ/Δ*^ PTCL is shown in blue and yellow, respectively. Corresponding changes in gene expression for *Dnmt3a*^*Δ/Δ*^ PTCL relative to control CD8+ samples are shown in red.

To better understand deregulated molecular events in PTCL induced by mono or bi-allelic loss of Dnmt3a we performed global gene expression profiling of *Dnmt3a*^*Δ/Δ*^ and *Dnmt3a*^*+/-*^ PTCLs using RNA-seq. Comparison of gene expression patterns obtained from lymphomas to patterns obtained from normal CD8+ T cells revealed that *Dnmt3a*^*Δ/Δ*^ and *Dnmt3a*^*+/-*^ PTCLs shared strikingly similar expression profiles. In total, 737 (69%) overexpressed and 697 (79%) underexpressed genes were conserved between *Dnmt3a*^*Δ/Δ*^ and *Dnmt3a*^*+/-*^ PTCLs relative to CD8+ T cell controls (**Fig 7A and 7B and S8 Table**). We also identified 329 upregulated and 185 downregulated genes specific to *Dnmt3a*^*Δ/Δ*^ PTCL, as well as 650 upregulated and 549 downregulated genes specific to *Dnmt3a*^*+/-*^ PTCL (**Fig 7A and 7B and S8 Table**). Altogether, these data suggest that molecular events driving T cell transformation in *Dnmt3a*^*+/-*^ and *Dnmt3a*^*Δ/Δ*^ mice are likely conserved.

IPA of differentially expressed genes in PTCL identified 3 Inhibited Pathways common to both *Dnmt3a*^*+/-*^ and *Dnmt3a*^*Δ/Δ*^ PTCL (*Tec Kinase signaling*, *Type I Diabetes Mellitus Signaling*, *4-1BB Signaling in T Lymphocytes*) and 1 commonly Activated Pathways (*Cyclins and Cell Cycle regulation*) in *Dnmt3a*^*Δ/Δ*^ and *Dnmt3a*^*+/-*^ lymphomas (**S4 Fig**). The top 5 categories for “diseases and disorders” were identical for both *Dnmt3a*^*Δ/Δ*^ and *Dnmt3a*^*+/-*^ tumors (Inflammatory response, Immunological disease, Connective tissue disorder, Inflammatory disease and Skeletal and muscular disorders), further illustrating the similarities between their molecular landscapes.

Comparison of methylation and gene expression revealed that 71 genes (14%) whose promoters were hypomethylated in PTCL were associated with overexpression (**Fig 7C and 7D,** referred to herein as HOT genes–Hypomethylated and overexpressed in TCL). In contrast, we detected only three genes—*CD226*, *Fhit*, and *Emp1*—whose hypermethylation correlated with underexpression, suggesting that most of the cancer-specific hypermethylation has little effect on gene expression and tumor progression (**Fig 7C**). Further analysis revealed 54 genes (7%) whose predicted enhancer regions were hypomethylated in PTCL and were also overexpressed, whereas only 3 genes with hypermethylated enhancers were downregulated (**Fig 7C and S5 Fig**). Altogether, these data demonstrate that hypomethylation affects gene expression on a broader scale than hypermethylation in mouse *Dnmt3a*^*Δ/Δ*^ PTCL and thus may functionally contribute to a disease development.

### Gene expression changes are partially conserved between mouse and human PTCL

**Fig 8:**
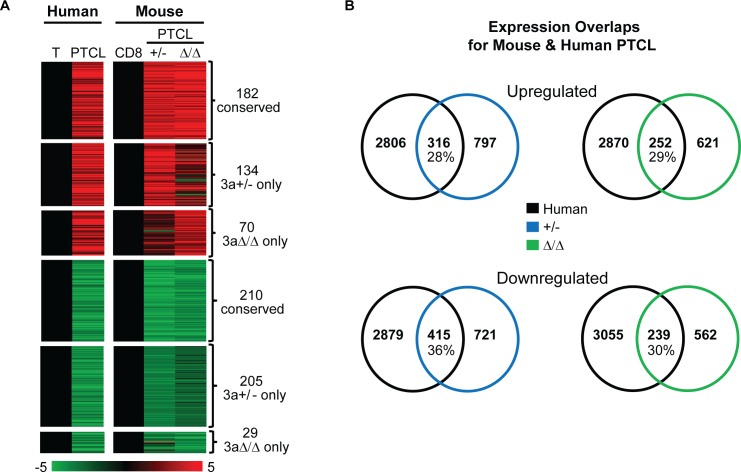
Gene expression changes are partially conserved between mouse and human PTCL. (A) Heat maps derived from global expression profiling for genes differentially expressed in both human PTCL (PTCL) relative to normal tonsil T cells (T) and in *Dnmt3a*^*+/-*^ PTCL (+/-) and/or *Dnmt3a*^*Δ/Δ*^ PTCL (Δ/Δ) relative to normal *Dnmt3a*^*+/+*^ CD8+ cells. 182 genes were commonly overexpressed in human PTCL, *Dnmt3a*^*+/-*^ PTCL, and *Dnmt3a*^*Δ/Δ*^ PTCL, while 210 genes were commonly underexpressed in all three tumor types. 134 overexpressed and 205 underexpressed genes were specific to human PTCL and *Dnmt3a*^*+/-*^ PTCL, whereas 70 overexpressed and 29 underexpressed genes were only observed in human PTCL and *Dnmt3a*^*Δ/Δ*^ PTCL. For microarray data (human samples), genes with a fold change >1.5 and a P-value <0.05 were considered significant. For RNA-seq data (mouse samples), genes with a fold change >2 and a p-value <0.05 were considered significant. A color bar displays fold change in gene expression with overexpressed shown in red and underexpressed in green. (B) Venn diagrams showing overlaps in gene expression between human PTCL (black) and mouse *Dnmt3a*^*+/-*^ PTCL (blue) and *Dnmt3a*^*Δ/Δ*^ PTCL (green), as determined in panel A of the figure.

To determine the extent of similarity between mouse and human disease on the molecular level, we compared gene expression signatures obtained from mouse PTCL to those derived from human PTCL. We utilized microarray data obtained on a set of five normal tonsil T cells and three human PTCL samples with predicted inactivating Dnmt3a mutations [19]. When we compared expression of genes deregulated in human PTCL to those genes deregulated in either *Dnmt3a*^*+/-*^ PTCL, we identified 316 (28%) overexpressed and 415 (36%) underexpressed genes that were shared between human and *Dnmt3a*^*+/-*^ PTCL (**Fig 8A and 8B and S9 Table**). Fewer genes (252 overexpressed and 239 underexpressed) were shared between human PTCL and *Dnmt3a*^*Δ/Δ*^ PTCL, suggesting that the transcriptome of lymphomas induced by loss of a single Dnmt3a allele more so resembles human disease than those that arise do to full inactivation of Dnmt3a (**Fig 8A and 8B and S9 Table**). The extent of overlap in up-and downregulated genes was significant for all comparisons (P<0.01), suggesting that similar molecular events may drive PTCL in both species.

### *Jdp2* is hypomethyalted and overexpressed in mouse and human PTCL

**Fig 9:**
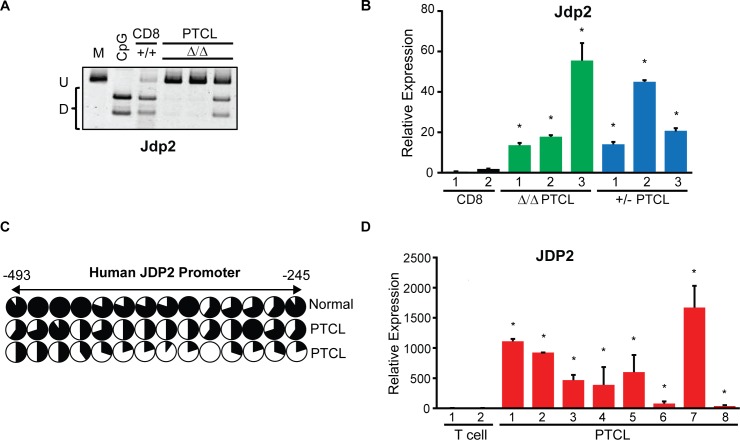
Jdp2 is hypomethylated and overexpressed in human and mouse PTCL. (A) COBRA analysis of mouse Jdp2 promoter methylation in three independent *Dnmt3a*^*Δ/Δ*^ PTCL samples. Undigested (U) and digested (D) fragments correspond to unmethylated and methylated DNA, respectively. Control PCR fragments generated from fully methylated mouse genomic DNA that is undigested (M) or digested (CpG) are shown. (B) Normalized gene expression of Jdp2 in mouse CD8+ control, *Dnmt3a*^*+/-*^ PTCL, and *Dnmt3a*^*Δ/Δ*^ PTCL samples by qRT-PCR. Data presented are the average of two independent experiments. Error bars show standard deviation and an asterisk (*) denotes a p<0.05 (student t-test). (C) Bisulfite sequencing of the JDP2 promoter in normal human CD3+ T cells and in two independent human PTCL samples. Circles represent individual CpGs within the promoter. Black and white areas denote the relative portion of methylated and un-methylated sequence reads at a CpG, respectively. (D) Normalized gene expression of JDP2 in normal human CD3+ T cells and human PTCL samples, by qRT-PCR. Data presented are the average of two independent experiments. Error bars show standard deviation and an asterisk (*) denotes a p<0.05 (student t-test).

Because promoter hypomethylation resulted in upregulated gene expression in PTCL we next asked whether any of the HOT genes may have potential oncogenic functions in the development of T cell lymphomas and whether such genes are also hypomethylated and overexpressed in human PTCLs. One such candidate gene with oncogenic function in the T cell compartment—Jun Dimerization Protein 2 protein (Jdp2)—is a component of the AP-1 transcription factor that was reported to negatively regulate *Trp53* and promote the development of T cell leukemia in mice [20]. Consistently with global WGBS data, Jdp2 was hypomethylated in *Dnmt3a*^*Δ/Δ*^ PTCLs as determined by COBRA (**Fig 9A**). Likewise, with RNA-seq data, analysis of *Jdp2* transcript levels by qRT-PCR confirmed overexpression of *Jdp2* in *Dnmt3a*^*+/-*^ and *Dnmt3a*^*Δ/Δ*^ PTCL samples (**Fig 9B**). Next, we analyzed the methylation status of the JDP2 promoter in human CD3 T cells and PTCL samples and found that like in Dnmt3a-deficient mouse PTCL, the JDP2 promoter is hypomethylated in human PTCL relative to controls (**Fig 9C**). To determine whether overexpression of JDP2 occurs in human PTCL, we compared transcript levels in a set of 8 human PTCLs to normal CD3+ T cells. This analysis showed ~20–1,700-fold increase in JDP2 levels in human PTCL relative to normal T cells (**Fig 9D**). These data demonstrate that JDP2 promoter hypomethylation correlates with its overexpression in human PTCL. To evaluate the role of Jdp2 in tumor maintenance we used an shRNA construct to knockdown the levels of Jdp2 in a Dnmt3a-deficient MYC-induced PTCL cell line. However, decrease in the levels of Jdp2 in this cell line did not affect cellular growth, suggesting Jdp2 is not required for tumor maintenance in such setting (**S6A and S6B Fig**). Overall, our data indicate that methylation likely plays a role in the regulation of Jdp2 in mouse and human PTCL.

### P53 is downregulated in pretumor thymocytes and in *Dnmt3a*^*+/-*^ and *Dnmt3a*^*Δ/Δ*^ PTCLs

**Fig 10:**
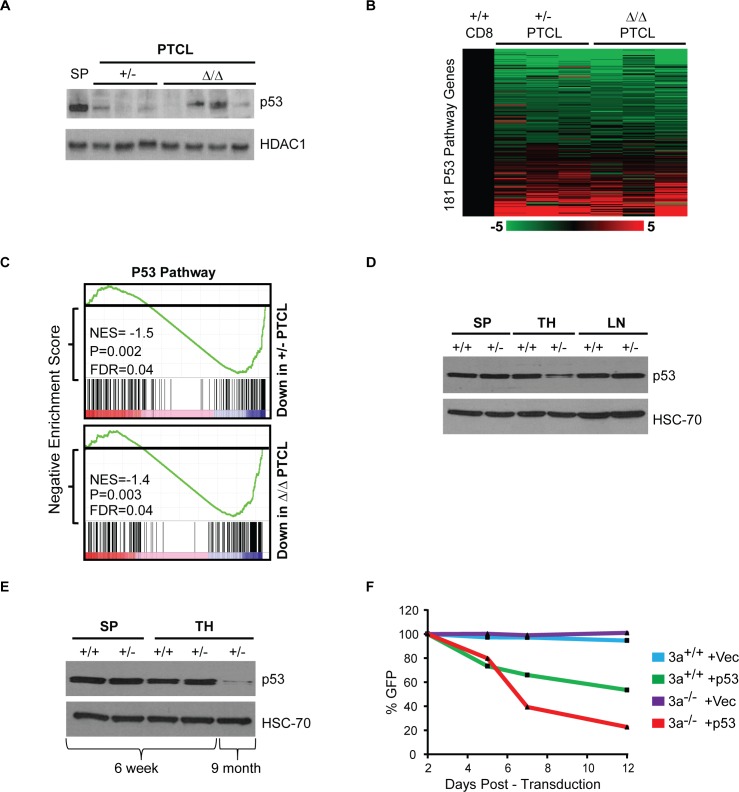
P53 is downregulated in pretumor thymocytes and in *Dnmt3a*^*+/-*^ and *Dnmt3a*^*Δ/Δ*^ PTCLs. (A) Immunoblot showing p53 protein levels in *Dnmt3a*^*+/+*^ control spleen (SP), *Dnmt3a*^*+/-*^ PTCL (+/-) *Dnmt3a*^*Δ/Δ*^ PTCL (Δ/Δ) samples. HDAC1 is shown as a loading control. (B) Heatmap showing fold change in gene expression data derived from RNA-seq of 181 p53 pathway genes identified through GSEA for *Dnmt3a*^*+/-*^ PTCL and *Dnmt3a*^*Δ/Δ*^ PTCL relative to CD8+ controls. (C) RNA-seq expression profiles on *Dnmt3a*^*+/-*^ PTCL, and *Dnmt3a*^*Δ/Δ*^ PTCL were subjected to GSEA to identify enriched signatures. Each group was run as a pairwise comparison to normal CD8+ cells. In both tumor groups, the p53 pathway was identified as being significantly downregulated relative to controls. Normalized enrichment scores (NES), false discovery rate (FDR) and P-values are shown for each analysis. Black bars indicate individual genes within the pathway. Red indicates genes with high expression and blue indicates low expression in tumors relative to controls. (D) Immunoblot showing p53 protein levels in *Dnmt3a*^*+/+*^ (+/+) and *Dnmt3a*^*+/-*^ (+/-) cells isolated from the spleen (SP), thymus (TH), and lymph node (LN) of 9 month old mice with no signs of lymphoma development. HSC-70 is shown as a loading control. (E) Immunoblot showing p53 protein levels in *Dnmt3a*^*+/+*^ (+/+) and *Dnmt3a*^*+/-*^ (+/-) cells isolated from the spleen (SP) and thymus (TH) of 6 week and 9 month old mice with no signs of lymphoma development. HSC-70 is shown as a loading control. (F) Relative percentage of EGFP positive cells as determined by FACS for *Dnmt3a*^*+/+*^ (+/+) and *Dnmt3a*^*-/-*^ (-/-) lymphoma cell lines infected with either MSCV-IRES-EGFP (Vec) or MSCV-IRES-p53-EGFP (p53). EGFP measurements were taken at multiples time points and normalized by the percentage of EGFP positive cells observed 2 days post-transduction.

Because Jdp2 was reported to negatively regulate p53 transcript levels we analyzed *Trp53* expression by qRT-PCR. Despite a 10-70-fold increase in *Jdp2* levels in *Dnmt3a*^*+/-*^ and *Dnmt3a*^*Δ/Δ*^ PTCL samples, we did not observe any effects on *Trp53* transcript levels (**S7 Fig**), suggesting that in this setting Jdp2 overexpression has no direct effect on p53 transcription. However, analysis of p53 protein levels showed downregulation of p53 in all *Dnmt3a*^*+/-*^ tumors and 3/4 *Dnmt3a*^*Δ/Δ*^ tumors, suggesting that Jdp2 may regulate p53 at the protein level or p53 downregulation in tumors occurs independently of Jdp2 overexpression (**Fig 10A**). Gene Set Enrichment Analysis (GSEA) using RNA-seq data from normal *Dnmt3a*^*+/-*^ and *Dnmt3a*^*Δ/Δ*^ PTCL revealed significant downregulation of the p53 pathway genes in both settings (**Fig 10B and 10C and S10 Table**). To determine when p53 is downregulated during lymphomagenesis, we next measured protein levels in spleens, lymph nodes and thymi isolated from 9 months old *Dnmt3a*^*+/+*^ and *Dnmt3a*^*+/-*^ mice. At this age, *Dnmt3a*^*+/-*^ mice did not show any sign of lymphomagenesis or cellular changes in the hematopoietic compartment (**S8 Fig**). Interestingly, whereas p53 levels in spleens and lymph nodes were similar between *Dnmt3a*^*+/+*^ and *Dnmt3a*^*+/-*^ settings, p53 was downregulated in thymocytes of *Dnmt3a*^*+/-*^ mice (**Fig 10D**). To determine whether downregulation of p53 occurs as a direct response to Dnmt3a monoallelic loss we analyzed p53 protein levels in splenocytes and thymocytes of 6 weeks old *Dnmt3a*^*+/+*^ and *Dnmt3a*^*+/-*^ mice. This analysis revealed no apparent differences in p53 levels, suggesting that loss of one allele of Dnmt3a is insufficient to downregulate p53 at this time point (**Fig 10E**). Because we did not succeed in establishing CD8+ lymphoma cell lines, we utilized previously generated MYC-induced *Dnmt3a*^*+/+*^ and *Dnmt3a*^*-/-*^ T cell lymphoma cell lines [7], along with MSCV-IRES-p53-GFP overexpressing both p53 and EGFP [21], to evaluate the role of p53 in lymphomagenesis. Overexpression of p53 induced selection against EGFP-positive cells in both *Dnmt3a*^*+/+*^ and *Dnmt3a*^*-/-*^ T cell lymphoma cell lines, suggesting that exogenous p53 inhibited cellular proliferation *in vitro* (**Fig 10F**). This result suggests that low p53 levels are important for tumor maintenance *in vitro* and therefore downregulation of p53 *in vivo* is likely an important event in tumorigenesis. Altogether, these data suggest that downregulation of p53 is chronologically an intermediate event in lymphomagenesis and therefore likely a relevant in initiation/progression of lymphomagenesis and may be mediated by upregulation of Jdp2.

## Discussion

In this study we show that loss of Dnmt3a in HSPCs in *EμSRα-tTA;Teto-Cre;Dnmt3a*^*fl/fl*^*; Rosa26LOXP*^*EGFP/EGFP*^ mice not only results in the development of CLL as we reported previously [16,17] but also in the development of peripheral T cell lymphomas in ~40% of *Dnmt3a*^*Δ/Δ*^ mice either alone or in combination with CLL. We further show that not only complete inactivation but also a reduction in Dnmt3a levels results in the development of PTCL in 10% of *Dnmt3a*^*+/-*^ mice. Lymphomas that develop in both *Dnmt3a*^*Δ/Δ*^ and *Dnmt3a*^*+/-*^ mice are exclusively CD8+CD4- mature T cell lymphomas. Importantly, *Dnmt3a*^*+/-*^ PTCLs retain expression of the Dnmt3a *wild-type* allele. Thus, consistent with heterozygous mutations of Dnmt3a found in human T cell malignancies, Dnmt3a is a haploinsufficient tumor suppressor gene in the prevention of mouse mature CD8+CD4- T cell lymphomas.

The growing number of various malignant phenotypes observed in the hematopoietic system with Dnmt3a-deficiency in mice raises questions about the nature of deregulated events induced by Dnmt3a inactivation. Because Dnmt3a is a methyltransferase, we were interested in finding whether genes deregulated in a methylation dependent manner could provide clues towards understanding the pathobiology of *Dnmt3a*^*Δ/Δ*^ PTCLs. A combined analysis of global methylation and gene expression identified promoter hypomethylation as a major deregulated event in PTCL development in the absence of Dnmt3a with as many as 500 genes hypomethylated in lymphomas. Of these genes, expression of as many as 71 genes (14%) was upregulated in tumors. Since Dnmt3a has now been shown to be a tumor suppressor in the prevention of a number of hematologic malignancies in a variety of biological settings [13–17], it is therefore possible that promoter hypomethylation along with gene upregulation may be either a contributing factor or even the primary event driving the initiation/progression of tumor development. In such a scenario, proto-oncogenes are silenced in normal cells but are progressively hypomethylated and overexpressed resulting in cellular transformation. Analysis of data derived from *Dnmt3a*^*Δ/Δ*^ lymphomas identified several putative drivers of T cell transformation whose promoters were hypomethylated and overexpressed in tumors (HOT genes). One such HOT gene is the Interleukin-2 receptor *Il2rb*, a component of the IL-2 signaling pathway that is important for the growth of T lymphocytes. Inappropriate activation of this pathway may promote unchecked proliferation of T cells, thus contributing to tumorigenesis [22]. The HOT gene, *Stat1*, participates in cytokine signaling in T cells and has been reported to be significantly overexpressed in human PTCL-NOS [23]. In a mouse model of *v-abl*-induced leukemia, *Stat1*^*-/-*^ mice were partially protected from the development of leukemia, demonstrating that *Stat1* possesses tumor-promoting activity [24]. Another HOT gene, *Trim14* was demonstrated to have oncogenic function in tongue squamous cell carcinoma cell lines by activating the NF-κB pathway [25]. Whereas HOT genes represent good candidates to explain the tumor suppressor function of Dnmt3a, demonstration of a causative oncogenic role in initiation/progression of lymphomagenesis for any of these genes is challenging as it requires long-term *in vivo* experiments in mice. Thus, only future functional studies can address the importance of these genes in the pathogenesis of *Dnmt3a*^*Δ/Δ*^ PTCLs.

An additional HOT gene with predicted oncogenic activity is Jun Dimerization Protein 2 (JDP2), which we found to be hypomethylated and overexpressed not only in mouse PTCL but also in human PTCLs. Jdp2 protein is a component of the AP-1 transcription factor complex that represses transactivation mediated by the Jun family of proteins and it plays a role in AP-1-mediated responses in UV-induced apoptosis and cell differentiation [26]. Jdp2 was reported to promote liver transformation as JDP transgenic mice displayed potentiation of liver cancer, higher mortality and increased number and size of tumors [27]. Importantly, Jdp2 was identified in a screen for oncogenes able to collaborate with the loss of p27^kip1^ cyclin-dependent inhibitor to induce lymphomas [28]. Altogether these data along with our findings suggest that upregulation of Jdp2 induced by loss of Dnmt3a might be a contributing factor to the development of PTCL. Because Jdp2 was reported to negatively regulate *Trp53* on a transcriptional level and promote the development of T cell leukemia in mice [20] we tested whether Trp53 levels are affected in *Dnmt3a*^*Δ/Δ*^ PTCLs. Despite a 15–70 fold increase in *Jdp2* levels we did not observe any changes in *Trp53* transcript levels, suggesting that in this setting Jdp2 overexpression has little effect on p53 transcription. However, western blot revealed decrease in p53 protein in the majority of tumor samples, suggesting that Jdp2 may regulate p53 by other mechanisms or p53 downregulation occurs through an independent pathway not involving Jdp2. Regardless of the mechanism by which p53 is downregulated in tumors, decreased p53 protein is likely contributing to CD8+ T cell transformation due to its strong tumor suppressor function in T cell compartment. For example, it was previously reported that *Trp53*^*-/-*^ mice are highly susceptible to spontaneous tumor development, with the majority of mice developing immature CD4+CD8+ thymic lymphomas [29]. To the best of our knowledge, there are no studies in mice demonstrating the tumor suppressor function of p53 specifically in CD8+ T cell lymphomas. However, a loss of the region containing the p53 gene on chromosome 17 was observed in human primary cutaneous CD8+ cytotoxic T cell lymphoma, suggesting that low p53 levels could be involved in the pathogenesis of human CD8+ PTCL [30]. The fact that p53 was downregulated in thymocytes isolated from 9 month old, but not 6 week old, *Dnmt3a*^*+/-*^ tumor-free mice indicates that p53 downregulation is chronologically an intermediate event in lymphomagenesis and this strongly suggest that this event is relevant in the initiation/progression of CD8+ PTCL. Consistent with downregulation of p53 protein levels, GSEA revealed suppression of p53 pathway genes in both *Dnmt3a*^*+/-*^ and *Dnmt3a*^*Δ/Δ*^ PTCL tumors, such as GADD45a, ZFP36L1, and KLF4. Studies using *Gadd45a*^*-/-*^ mice found that ablation of Gadd45a in lymphoma-prone AKR mice decreased the latency and increased the incidence of T cell lymphomas, while deletion of Gadd45a on a p53 deficient background altered the tumor spectrum to heavily favor the development of T cell lymphomas [31]. Similarly, mice deficient for ZFP36L1 and ZFP36L2 displayed altered T cell development and readily succumbed to CD8+ T cell acute lymphoblastic leukemia [32]. KLF4 was identified to be mutated in pediatric T-ALL patients [33] and was shown to induced apoptosis in primary T-ALL cells [34].

These results suggest the downregulation of p53 target genes may contribute to T cell transformation in Dnmt3a-deficient mice. Altogether, these data indicate that downregulation of p53 is an important event during lymphomagenesis in *Dnmt3a*^*+/-*^ and *Dnmt3a*^*Δ/Δ*^ mice.

Promoter hypomethylation and p53 downregulation may not be the only relevant events involved in the development of PTCL in Dnmt3a-defficient mice. An additional DNA methylation change that could contribute to the development of PTCL in *Dnmt3a*^*Δ/Δ*^ mice is promoter hypermethylation, as it has been linked to the inactivation of tumor suppressor genes [35,36]. Although such changes would not be linked to Dnmt3a directly as inactivation of this enzyme is an initiating event of tumorigenesis, promoter hypermethylation mediated by other DNA methyltransferase and subsequent gene silencing could still drive tumorigenesis. In particular, in our previous studies we observed upregulation of Dnmt3b in Dnmt3a-defficient MYC-induced T cell lymphomas, suggesting that such an event may result in aberrant *de novo* methylation [7]. Surprisingly, despite identification of 50 genes whose promoters are hypermethylated in PTCL relative to CD8+ T cell controls, only *Fhit*, *CD226*, and *Emp1* were underexpressed. This raises a possibility that silencing of these genes contributes to PTCL development. *Fhit* is a predicted tumor suppressor gene that is frequently deleted in B cell malignancies, including Burkitt’s lymphoma and primary effusion lymphoma [37,38]. Furthermore, *in vivo* studied using *Fhit*^*+/−*^ mice found that loss of a single allele of *Fhit* increased susceptible to carcinogen-induced tumor development in the esophagus and forestomach, further demonstrating the role of Fhit as a tumor suppressor [39]. CD226 is expressed on different hematopoietic cells including CD8+ T cells and contributes to their activation, expansion and differentiation but its deficiency in mice did not induce lymphomas, suggesting that this gene may not be a tumor suppressor gene [40]. Similarly, *Emp1* overexpression correlated with enhanced cell proliferation and poor prognosis in B cell precursor ALL leukemia, suggesting an oncogenic function of this gene at least in some hematologic malignancies [41]. However, the possible role of *Fhit*, *CD226*, and *Emp1* as tumor suppressors in CD8+ *Dnmt3a*^*Δ/Δ*^ PTCL is unclear. Thus, the role of hypermethylation and silencing in disease development and progression in mouse *Dnmt3a*^*Δ/Δ*^ PTCL will require further investigation.

One of the interesting findings presented here is the exclusive sensitivity of CD8+ T cell to transformation in *Dnmt3a*^*+/-*^ and *Dnmt3a*^*Δ/Δ*^ mice. This is not a consequence of impaired T cell development as we previously reported that loss of Dnmt3a does not affect the development of hematopoietic lineages [16]. Therefore, the reason as to why CD8+ but never CD4+ or CD4+CD8+ T cells become transformed in the absence of Dnmt3a is unclear at present. We speculate that the epigenome of CD8+ T cells is more dependent on Dnmt3a than other T cell types or CD8+ T cells may acquire genetic alterations that collaborate with epimutations more readily than other T cells. Of note, a differential sensitivity of T cell subtypes to transformation has been observed in response to infection by HTLV-1, which predominantly transforms CD4+ T cells, while HTLV-2 mainly transforms CD8+ T cells [42,43]. Further studies will have to clarify whether the methylome of CD4 cells is more resistant to the lack of Dnmt3a as well as the nature of events responsible for CD8+ T cell transformation.

Another interesting finding from our study is association of transcription factor (TF) binding motifs with regions hypomethylated and hypermethylated in Dnmt3a-defficient PTCL. Analysis of TF binding sites found motifs for three TFs—AML1, NF-κB, and OCT1 –that were enriched in hypomethylated DMRS, suggesting their potential role in maintenance methylation mediated by Dnmt3a. In such a scenario, interaction of these factors with Dnmt3a may determine which specific loci Dnmt3a is targeted to. Interestingly, the p50 subunit of the NF-κB transcription factor was reported to interact with Dnmt3a in a glioblastoma cell line [44]. Similarly, we also observed association of binding sites for Ap-2rep, SOX5, and myogenin with hypermethylated DMRS. Whether any of these transcription factors play role in aberrant promoter hypo- or hypermethylation remains to be determines.

Altogether, our data identify Dnmt3a as a critical tumor suppressor gene in the prevention of B- and T cell malignancies and link decreased Dnmt3a levels to decrease in p53, which may functionally contribute to the development of CD8+ PTCL. These data along with its documented role in prevention of myeloid malignancies defines Dnmt3a as a protector of the methylome critical for safeguarding normal hematopoiesis.

## Materials and Methods

### Mouse studies

*EμSRα-tTA* and *Dnmt3a*^*2loxP/2loxP*^ (*Dnmt3a*^*fl/fl*^) mice were acquired from D.W. Felsher (Stanford University) and R. Jaenisch (Whitehead University), respectively. *ROSA26*^*EGFP*^ and *Teto-Cre* mice came from the Jackson Laboratories. All transgenic mice were generated using standard crosses. All mice used in these studies were of the FVB/N background and were generated using standard genetic crosses. To obtain mice with a germline transmission of the *Dnmt3a*^*-*^ allele, we crossed *EμSRα-tTA;Teto-Cre;Dnmt3a*^*fl/fl*^ mice with FVB mice, taking advantage of our observation that the *EμSRα-tTA* transgene is expressed in germ cells. To generate *Dnmt3a*^*+/-*^ we subsequently bread out transgenes by crossing obtained mice with FVB mice. PCR-based genotyping of genomic DNA isolated from the tails was used to confirm genotypes. *Dnmt3a*^*+/-*^ mice were harvested at the experimental end point of 16 months.

### Human lymphoma samples

Human peripheral T cell lymphoma tissue samples were acquired from Cooperative Human Tissue Network, a National Cancer Institute supported resource (www.chtn.org).

### FACS analysis

All analysis was performed at the Flow Cytometry Facility at the University of Nebraska Medical Center. Single cell suspensions were generated from mouse organs and labeled with fluorescently conjugated antibodies (eBioscience). Data was collected using the LSR II (BD Biosciences) and analyzed using BD FACSDiva software (BD Biosciences). Immunopheotypic criteria for normal and malignant cellular populations analyzed by flow cytometry are as follows: Cytotoxic T cells (EGFP-negative CD3+CD8+ without population expansion), CD8+ peripheral T cell lymphomas (EGFP+CD3+CD8+ with population expansion), B-1a cells (EGFP-negative CD19+B220+CD5+ without population expansion), chronic lymphocytic leukemia (EGFP+CD19+B220+CD5+ with population expansion). Clonality was assessed using the mouse Vβ TCR Screening Panel (BD bioscience) which uses FITC-conjugated monoclonal antibodies to recognize mouse Vβ 2, 3, 4, 5.1 and 5.2, 6, 7, 8.1 and 8.2, 8.3, 9, 10b, 11, 12, 13, 14, and 17a T cell receptors.

### Western blot

Western blots were performed as previously described [6] with use of the following antibodies: Dnmt3a (H-295, Santa Cruz), γ-Tubulin (H-183, Santa Cruz), p53 (SC-6243, Santa Cruz), HDAC1 (ab7028, Abcam), and HSC-70 (SC-7298, Santa Cruz).

### Combined bisulfite restriction analysis (COBRA) and bisulfite sequencing

COBRA analysis was carried out as described previously [5,6]. Mouse and human bisulfite specific primers are shown in S11 Table.

#### Whole genome bisulfite sequencing (WGBS)

DNA was isolated from FACS sorted CD8+ T cells obtained from the spleens of control FVB/N (CD8+CD3+) and *Dnmt3a*^*Δ/Δ*^ terminally ill PTCL mice (EGFP+CD8+CD3+). The WGBS libraries were prepared and sequenced in the DNA Services facility at the University of Illinois at Urbana-Champaign, Roy J. Carver Biotechnology Center / W.M. Keck Center using two lanes for each sample on the Illumina HiSeq2500 sequencer with paired-end 160bp reads. Each lane produced over 310 million reads. Sequence tags were aligned with the mouse genome (Dec. 2011 mus musculus assembly mm10, Build 38) using the methylated sequence aligner Bismark [45] by the University of Nebraska Epigenomics Core facility. The resulting data file contains the percent methylation at each CpG measured. Each individual CpG was retained and percent methylation determined only if it was represented by ≥5 individual sequences. Correlation based, average linkage hierarchical clustering of genome location matching CpG methylation percentages per sample was performed using the R software package RnBeads [46]. Genome location matching differentially methylated cytosines (DMCs) and differentially methylated regions (DMRs) were determined using the R software package DSS [47]. DMCs were determined by first smoothing the raw percent methylation values based on a moving average algorithm and smoothing span of 500 bases. DMRs were then determined based on average DMC methylation change of 30 percent or greater, at least 50 percent or greater individual DMC, p-values less than 0.05, minimum base pair length of 100, minimum of three DMLs represented, and the resulting DMRs were averaged if they were closer than 50 bases. Circos plots [48] were generated to visualize DMRs that had at least a 100 base overlap with genomic promoters defined as 1500 bases upstream of the transcription start site (TSS) to 500 bases downstream of the TSS. DMRs were aligned with the mouse genomic repeats. Genomic repeats were acquired from the UCSC Genome table browser based on the RepeatMasker program [49]. The repeat was retained if the overlap between the DMR and repeat was more than 25 percent of the length of the repeat. For analysis of enhancer methylation, DMRs were aligned using the bedtools intersect routine with the 48,415 2000bp enhancer annotated regions that were determined based on histone marks (high H3K4me1/2 and low H3K4me3) [50, 51]. WGBS data is available for download through the NCBI Gene Expression Omnibus (GSE78146).

### Transcription factor enrichment

The mouse mm9 MotifMap database containing 2,237,515 transcription factor motifs [52,53] (http://motifmap.ics.uci.edu/) was used to align transcription factors motifs present within promoters (-1500 to +500 TSS) that contained a significant hypo- or hypermethylated DMR using the Bedtools intersect routine [51]. For a control comparison 12 random sets of promoters (500 promoters each were used for the hypometylated controls and 50 for the hypermethylated) were selected from the UCSC known mm9 genes database using the Excel RANDBETWEEN function and sorting from highest to lowest number. The abundance of each transcription factor within the DMR promoters and random promoters were counted using the Excel COUNTIF function. P-values were calculated used a Wilcoxon sign rank test. Only P<0.05 were considered significant.

### Reduced representation bisulfite sequencing

Splenic CD3+CD8+ T cells were isolated by FACS sorting from two *Dnmt3a* ^*Δ/Δ*^ mice with PTCL. Age-matched control T cells were FACS-sorted from spleens of FVB/N mice (n = 2). Genomic DNA was isolated using standard protocols. The RRBS libraries were prepared and sequenced at the Medical Genome Facility at the Mayo Clinic and ran on an Illumina HiSeq2500 sequencer. The Streamlined Analysis and Annotation Pipeline for RRBS data (SAAP-RRBS) was specifically designed to analyze RRBS data [54]. This software was used to align and determine the methylation status of CpGs associated with this type of restriction digest high throughput method. Sequences were initially aligned with genome mm9 then converted to mm10 using the UCSC Genome Browser Batch Coordinate Conversion (liftOver) utility. The methylation heat map was generated by taking the averages for all differentially methylated CpGs for a promoter (-1500 to +500 base pairs relative to the transcription start site). Promoters were only considered to be differentially methylated if one or more CpG sites showed a 30% change in methylation. RRBS data is available for download through the NCBI Gene Expression Omnibus (GSE78146).

### RNA-seq

RNA was isolated as previously described [6] from FACS sorted CD8+ T cells obtained from spleens of control FVB/N (CD8+CD3+) and *Dnmt3a*^*Δ/Δ*^ terminally ill PTCL mice (EGFP+CD8+CD3+). Library generation was performed using the TruSeq mRNA kit. The resulting libraries were sequenced on the Illumina HiSeq 2000 platform using paired-end 100bp runs (SeqMatic, Fremont, CA). The resulting sequencing data was first aligned using TopHat and mapped to the Mus musculus UCSC mm10 reference genome using the Bowtie2 aligner [55]. Cufflinks 2 was used to estimate FPKM of known transcripts, perform de novo assembly of novel transcripts, and calculate differential expression [56]. For differentially expressed genes, we considered those genes with a fold change ≥ 2 and a p-value < 0.05 to be significant. RNA-seq data is available for download through the NCBI Gene Expression Omnibus (GSE78146).

### Quantitative real-time qRT-PCR

qRT-PCR was performed as previously described [6]. Mouse real time primer sequences used in experiments presented are shown in S11 Table.

### Statistical methods

Data was compared in Microsoft Excel using Student’s t-test (p<0.05 considered significant) or other appropriate statistical comparison listed elsewhere in materials and methods.

### Histology

H&E staining was performed using standard protocols by the University of Nebraska Medical Center Tissue Science Facility.

### Microarray

Microarray data was downloaded from the NCBI Gene Expression Omnibus. We compared gene expression of 5 normal Tonsil T cells samples (GSE65135) to 3 PTCL samples in which DNMT3A was reported to be mutated (GSE58445) [19]. Datasets were generated with Affymetrix U133 plus 2 arrays and analyzed using Affymetrix Expression Console and Transcriptome Analysis Console (v3.0). Data was analyzed using a one-way between-subject ANOVA to generate p-values and identify differentially expressed genes (p-value < 0.05 and fold change >1.5). Genes differentially expressed in human PTCL were compared to those genes identified as being over- or underexpressed in mouse *Dnmt3a*^*+/-*^ or *Dnmt3a*^*Δ/Δ*^ relative to CD8+ T cell controls (RNAseq, Fold change >2, p<0.05).

### Ingenuity pathway analysis

All differentially expressed genes (p<0.05, fold change >2, analyzed by Cufflinks V2.0) for *Dnmt3a*^*+/-*^ PTCL relative to *wild-type* CD8+ T cells and *Dnmt3a*^*Δ/Δ*^ PTCL relative to *wild-type* CD8+ T cells were imported into IPA software. Core analysis were performed to identify top ranking pathways and categories for differentially expressed genes. Activated and inhibited pathways (Z-score>1.5, p<0.05) common to both *Dnmt3a*^*+/-*^ and *Dnmt3a*^*Δ/Δ*^ PTCL are shown in S4 Fig. In Fig 3E, IPA core analysis was performed on highly expressed genes (FPKM ≥ 10) in *wild-type* CD8+ T cells and the top subcategories obtained in Physiological System, Development and Functions were displayed (P<0.05, for all subcategories).

### Gene set enrichment analysis (GSEA)

TopHat/Cufflinks/Cuffdiff RNA-seq gene-level read_group_tracking file was converted to GCT expression dataset and matching phenotype model using the Read_group_trackingToGct module (http://www.broadinstitute.org/cancer/software/genepattern/modules/docs/Read_group_trackingToGct/1).Gene Set Enrichment Analysis (GSEA, http://www.broadinstitute.org/gsea/index.jsp) was used to test the relationship between RNA-Seq mRNA expression and the Hallmark Signature gene sets (http://software.broadinstitute.org/gsea/msigdb/genesets.jsp?collection=H). From this we concentrated our effort on the Hallmark p53 pathway gene set (http://software.broadinstitute.org/gsea/msigdb/cards/HALLMARK_P53_PATHWAY.html) that consisting of 180+ genes involved in p53 pathway and network.

### Retroviral shRNA knockdown experiments

HuSH 29-mer shRNA scrambled and shRNA Jdp2 in the retroviral vector pRFP-C-RS were purchased from Origene. Infections were performed as previously described [6], using a Dnmt3a-deficient MYC-induced CD4+ T cell lymphoma line [7]. Doubling time was calculated from each measured time point relative to the starting concentration of cells at Day 0. Each time point calculation of doubling time was considered a replicate measure and was averaged other measurements per experimental condition.

### P53 overexpression in murine T cell lymphoma cell lines

The pMSCV-IRES-EGFP (“Vector”) and pMSCV-bla-p53(WT)-IRES-EGFP (“p53”), a kind gift from Dr. Ute Moll [21], were transfected into the Phoenix_Eco_ packaging cells and retrovirus was produced. Transductions were performed as previously described [6], using *wild-type* Dnmt3a and Dnmt3a-deficient MYC-induced CD4+ T cell lymphoma lines [7]. The maximum percent of EGFP expressing cells per cell population was observed at 48 hours post-transduction and all subsequent EGFP data points were normalized to this time point. EGFP was measured periodically by flow cytometry on the LSRII available at the UNMC flow cytometry core facility. Cells were cultured in RPMI-1640 media supplemented with 10% FBS, 1% pen-strep-amphotericin B, 0.5% β-mercaptoethanol and split (1:3) to (1:5) every 3 days.

### Study approval

This study was performed in accordance with the guidelines established by the Guide for the Care and Use of Laboratory Animals at the National Institutes of Health. All experiments involving mice were approved by the IACUC (Protocol number: 08-083-10-FC) at the University of Nebraska Medical Center.

## Supporting Information

S1 Fig Loss of Dnmt3a induces CLL and PTCL in mice. (A) PCR based genotyping of the Dnmt3a locus using gDNA isolated from the thymus, lymph node, brain, skeletal muscle, kidney, and lung of *Dnmt3a*^*Δ/Δ*^ mice. gDNA isolated from tails of *Dnmt3a*^*+/+*^, *Dnmt3a*^*+/F*^, and *Dnmt3a*^*+/-*^ mice were used as controls. Knockout (KO), floxed, and wild-type (WT) bands are labeled. (B) Breakdown of phenotype spectrum in 42 *Dnmt3a*^*Δ/Δ*^ mice. (C) CD19 and CD5 expression in cells isolated from the spleen and blood of *Dnmt3a*^*+/+*^ control (+/+) and *Dnmt3a*^*Δ/Δ*^ CLL (Δ/Δ) mice, as determined by FACS. Percentage B-1a cells are shown in the red box. (D) IgD and IgM expression in cells isolated from the spleen of *Dnmt3a*^*+/+*^ control (+/+) and *Dnmt3a*^*Δ/Δ*^ CLL (Δ/Δ) mice, as determined by FACS. (E) FACS analysis showing CD8+CD3+ *Dnmt3a*^*Δ/Δ*^ PTCL cells do not express Nk-1 and CD16 markers. (F) Representative FACS diagram showing clonal TCR-Vβ expression in a *Dnmt3a*^*Δ/Δ*^ tumor. Control lymph node (+/+) is shown for reference. (G) Tumor burden as determined by average weight of spleens in *Dnmt3a*^*Δ/Δ*^ CLL (red) and *Dnmt3a*^*Δ/Δ*^ PTCL (blue). (H) FACS diagram showing the simultaneous expansion of B220+CD5+CD19+IgM+ B-1a cells and CD8+CD3+ T cells in the spleen of a *Dnmt3a*^*Δ/Δ*^ mouse.
(TIF)

S2 Fig Levels of Tet2 and RhoA are unchanged in *Dnmt3a*^*Δ/Δ*^ PTCL. Expression data from RNAseq (FPKM) for Tet2 and RhoA transcript levels in *Dnmt3a*^*+/+*^ CD8+ T cells and *Dnmt3a*^*Δ/Δ*^ PTCL samples.
(TIF)

S3 Fig RRBS analysis. (A) Heat map displaying 90 hypomethylated and 31 hypermethylated promoters identified by WGBS and confirmed by RRBS. RRBS data is shown as the average percent methylation of DMCS annotated to long promoters (-1500 to +500 relative to TSS) for *Dnmt3a*^*+/+*^ CD8+ T cells (n = 2) and *Dnmt3a*^*Δ/Δ*^ PTCL (n = 2). DMCS are defined by a ≥30% change in percent methylation in tumor samples compared to wild-type control samples. (B) RRBS confirmation of differentially methylated promoters identified by WGBS. Hypomethylated (top) and hypermethylated (bottom) genes confirmed by RRBS are shown in blue. Differentially methylated gene promoters identified by WGBS, but not confirmed RRBS are shown in orange. (C) The average number of CpG dinucleotides present in hypo- and hypermethylated promoter regions (-500 to +1500 bp relative to TSS) in *Dnmt3a*^*Δ/Δ*^ PTCL, as compared to *Dnmt3a*^*+/+*^ CD8+ T cells. Error bars show standard deviation.
(TIF)

S4 Fig IPA analysis. Summary of top categories, including “pathways” and “diseases and disorders”, derived from Ingenuity pathway analysis (IPA) of genes differentially expressed in both *Dnmt3a*^*+/-*^ and *Dnmt3a*^*Δ/Δ*^ PTCL relative to *Dnmt3a*^*+/+*^ CD8+ T cell controls. P<0.05 for all categories.
(TIF)

S5 Fig Overexpressed genes with enhancer hypomethylation. List of genes that are overexpressed and whose predicted enhancer regions are hypomethylated in *Dnmt3a*^*Δ/Δ*^ PTCL relative to *Dnmt3a*^*+/+*^ CD8+ T cell controls. Percent methylation derived from WGBS for enhancer regions for *Dnmt3a*^*+/+*^ CD8+ T cells and *Dnmt3a*^*Δ/Δ*^ PTCL is shown in blue (representing high levels of methylation) and yellow (representing low levels of methylation). Corresponding fold changes in gene expression (determined by RNA-seq) for *Dnmt3a*^*Δ/Δ*^ PTCL relative to control *Dnmt3a*^*+/+*^ CD8+ samples are shown in red.
(TIF)

S6 Fig Knockdown of Jdp2 in MYC-induced *Dnmt3a*^*-/-*^ T cell lymphoma does not affect cellular growth *in vitro*. (A) Average population doubling time for a *Dnmt3a*^*-/-*^ MYC-induced T cell lymphoma line infected with either scrambled shRNA (blue) or shRNA against Jdp2 (red). Error bars show standard deviation. (B) Normalized gene expression of *Jdp2* transcript levels as determined by qRT-PCR for a *Dnmt3a*^*-/-*^ MYC-induced T cell lymphoma line infected with either scrambled shRNA (blue) or shRNA against Jdp2 (red). Error bars show standard deviation.
(TIF)

S7 Fig *Tp53* transcript levels are unchanged in *Dnmt3a*^*+/-*^ and *Dnmt3a*^*Δ/Δ*^ PTCL. Normalized gene expression of *Tp53* transcript levels as determined by qRT-PCR in mouse *Dnmt3a*^*+/+*^ CD8+ T cell control, *Dnmt3a*^*+/-*^ PTCL, and *Dnmt3a*^*Δ/Δ*^ PTCL samples. Data presented are the average of two independent experiments. Error bars show standard deviation.
(TIF)

S8 Fig CD8+ T cells are not expanded in the spleen of a 9 months old *Dnmt3a*^*+/-*^ mouse. CD4 and CD8 expression in cells isolated from the spleen of 9 months old *Dnmt3a*^*+/+*^ (+/+) and *Dnmt3a*^*+/-*^ (+/-) mice, as determined by FACS. Percentage of cells in each quadrant are shown in red.
(TIF)

S1 Table Summary of TCR-Vβ expression in PTCL samples. *Dnmt3a*^*+/-*^ (n = 3) and *Dnmt3a*^*Δ/Δ*^ (n = 3) PTCL lymph node (LN) samples were analyzed by flow cytometry for the expression of 15 different TCR-Vβ surface markers. (-) indicates negative expression, whereas (+) denotes positive expression of TCR-Vβ markers.
(XLSX)

S2 Table CD8 promoter methylome. A heat map displaying methylation percentage of 21,712 promoters in *wild-type* CD8+ as determined by WGBS. Methylation percentage for individual CpGs were annotated to the promoter regions −300bp to +150bp relative to the transcription start site (TSS). Methylation percentages for all CpGs across the 450bp region were averaged to give a mean methylation value for each gene promoter. Lowly methylated promoters are shown in yellow and highly methylated promoters in blue. Data is presented in graphical form in Fig 3C.
(XLSX)

S3 Table CD8 promoter methylome and transciptome for core and long promoters. Heat map presentation of gene-matched promoter methylation for core promoters (tab 1) and long promoters (tab 2) and corresponding transcriptional expression (averaged FPKM values) in *wild-type* CD8+ cells, as determined by WGBS and RNA-seq for 15,732 genes. Highly expressed genes are denoted in red and lowly expressed genes are denoted in green. Data is presented in graphical form in Fig 3D.
(XLSX)

S4 Table Differentially methylated cytosines in PTCL. Table of differentially methylated cytosines in promoters (-1500 to +500bp relative to TSS) and gene bodies in *Dnmt3a*^*Δ/Δ*^ PTCL, as compared to wild-type CD8+ cells. Data is presented in graphical form in Fig 4A and 4B.
(XLSB)

S5 Table Core and long promoter heatmaps. Heat map comparing the methylation status of 21,712 promoters in *wild-type* CD8+ and *Dnmt3a*^*Δ/Δ*^ PTCL samples, as determined by WGBS. The averaged percent CpG methylation at core promoter regions (-300bp to +150bp relative to the TSS, *left*) and at long promoter regions (-1500bp to +500bp relative to the TSS, *right*) are displayed. Data is presented in graphical form in Fig 4C.
(XLSX)

S6 Table DMRS. List of differentially methylated regions (DMRS) located in long promoters, core promoters, gene bodies and predicted enhancers in *Dnmt3a*^*Δ/Δ*^ PTCL relative to CD8+ control. Data is presented in graphical form in Fig 4D.
(XLSX)

S7 Table RRBS confirmation. Heat map displaying 90 hypomethylated and 31 hypermethylated promoters identified by WGBS and confirmed by RRBS. RRBS data is shown as the average percent methylation of DMCS annotated to long promoters (-1500 to +500 relative to TSS) for *Dnmt3a*^*+/+*^ CD8+ T cells (n = 2) and *Dnmt3a*^*Δ/Δ*^ PTCL (n = 2). DMCS are defined by a ≥30% change in percent methylation in tumor samples compared to wild-type control samples. Data is presented in graphical form in S3 Fig.
(XLSX)

S8 Table RNA-seq data. Heat map of RNA-seq global expression data displaying differentially expressed genes in *Dnmt3a*^*+/-*^ and *Dnmt3a*^*Δ/Δ*^ PTCL (presented as fold change) relative to wild-type CD8+ cells, 737 genes were overexpressed and 697 genes were underexpressed in both *Dnmt3a*^*+/-*^ and *Dnmt3a*^*Δ/Δ*^ PTCL. 650 overexpressed and 549 underexpressed genes were specific to *Dnmt3a*^*+/-*^ PTCL, whereas 329 overexpressed and 185 underexpressed genes were only observed in *Dnmt3a*^*Δ/Δ*^ PTCL. Data is presented in graphical form in Fig 7A.
(XLSX)

S9 Table Overlaps in gene expression between human and mouse PTCL. Heat maps derived from global expression profiling for genes differentially expressed in both human PTCL (PTCL) relative to normal tonsil T cells (T) and in *Dnmt3a*^*+/-*^ PTCL (+/-) and/or *Dnmt3a*^*Δ/Δ*^ PTCL (Δ/Δ) relative to normal *Dnmt3a*^*+/+*^ CD8+ cells (presented as fold change). 182 genes were commonly overexpressed in human PTCL, *Dnmt3a*^*+/-*^ PTCL, and *Dnmt3a*^*Δ/Δ*^ PTCL, while 210 genes were commonly underexpressed in all three tumor types. 134 overexpressed and 205 underexpressed genes were specific to human PTCL and *Dnmt3a*^*+/-*^ PTCL, whereas 70 overexpressed and 29 underexpressed genes were only observed in human PTCL and *Dnmt3a*^*Δ/Δ*^ PTCL. For microarray data (human samples), genes with a fold change >1.5 and a P-value <0.05 were considered significant. For RNA-seq data (mouse samples), genes with a fold change >2 and a p-value <0.05 were considered significant. Data is presented in graphical form in Fig 8A.
(XLSX)

S10 Table Gene set enrichment analysis (GSEA) for p53 and MYC pathways. Heatmap showing fold change in gene expression data derived from RNA-seq of 181 p53 pathway genes identified through GSEA for *Dnmt3a*^*+/-*^ PTCL and *Dnmt3a*^*Δ/Δ*^ PTCL relative to CD8+ controls. Data is presented in graphical form in Fig 10B.
(XLSX)

S11 Table Primer sequences. List of mouse and human primer sequences used throughout the manuscript for COBRA and qRT-PCR analysis.
(XLSX)

S1 File Dnmt3a is not mutated in *Dnmt3a*^*+/-*^ PTCL samples. Sequencing results for the coding domain sequence for Dnmt3a CDS in one wild-type CD8+ sample and two *Dnmt3a*^*+/-*^ PTCL samples.
(DOCX)

S2 File RhoA and Tet2 are not mutated in *Dnmt3a*^*+/-*^ PTCL samples. Sequencing results for the coding domain sequence of RhoA and Tet2 in one wild-type CD8+ sample, three *Dnmt3a*^*+/-*^ PTCL samples, and three *Dnmt3a*^*Δ/Δ*^ PTCL samples.
(DOCX)
