# Supplementary figures and images for: Dnmt3a Is a Haploinsufficient Tumor Suppressor in CD8+ Peripheral T Cell Lymphoma
Source: PLoS Genet. 2016 Sep 30;12(9):e1006334. doi: 10.1371/journal.pgen.1006334 (PMC5045215; doi:10.1371/journal.pgen.1006334)

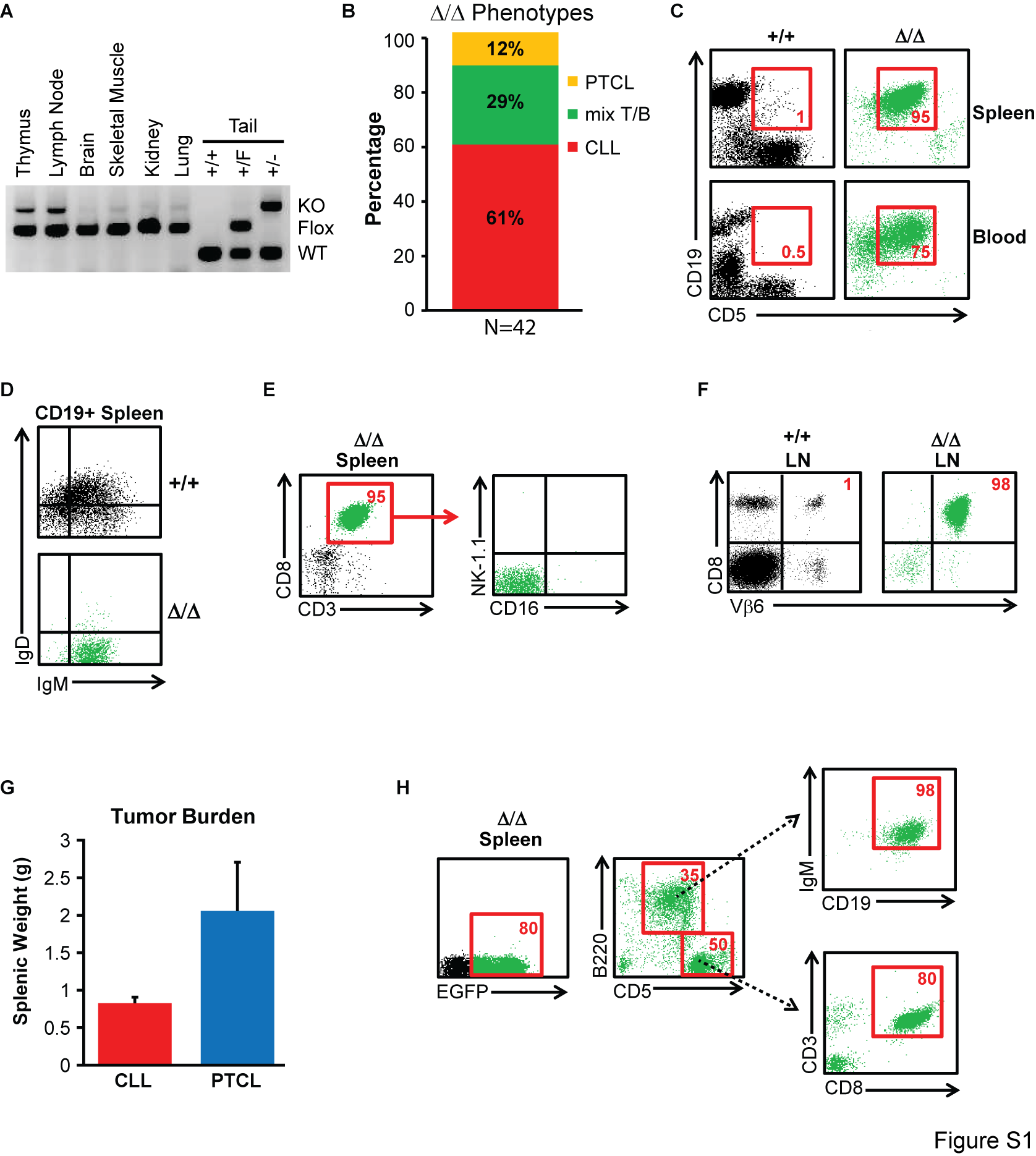

Supplement: S1 Fig — (A) PCR based genotyping of the Dnmt3a locus using gDNA isolated from the thymus, lymph node, brain, skeletal muscle, kidney, and lung of Dnmt3aΔ/Δ mice. gDNA isolated from tails of Dnmt3a+/+, Dnmt3a+/F, and Dnmt3a+/- mice were used as controls. Knockout (KO), floxed, and wild-type (WT) bands are labeled. (B) Breakdown of phenotype spectrum in 42 Dnmt3aΔ/Δ mice. (C) CD19 and CD5 expression in cells isolated from the spleen and blood of Dnmt3a+/+ control (+/+) and Dnmt3aΔ/Δ CLL (Δ/Δ) mice, as determined by FACS. Percentage B-1a cells are shown in the red box. (D) IgD and IgM expression in cells isolated from the spleen of Dnmt3a+/+ control (+/+) and Dnmt3aΔ/Δ CLL (Δ/Δ) mice, as determined by FACS. (E) FACS analysis showing CD8+CD3+ Dnmt3aΔ/Δ PTCL cells do not express Nk-1 and CD16 markers. (F) Representative FACS diagram showing clonal TCR-Vβ expression in a Dnmt3aΔ/Δ tumor. Control lymph node (+/+) is shown for reference. (G) Tumor burden as determined by average weight of spleens in Dnmt3aΔ/Δ CLL (red) and Dnmt3aΔ/Δ PTCL (blue). (H) FACS diagram showing the simultaneous expansion of B220+CD5+CD19+IgM+ B-1a cells and CD8+CD3+ T cells in the spleen of a Dnmt3aΔ/Δ mouse. (TIF) [file pgen.1006334.s001.tif]

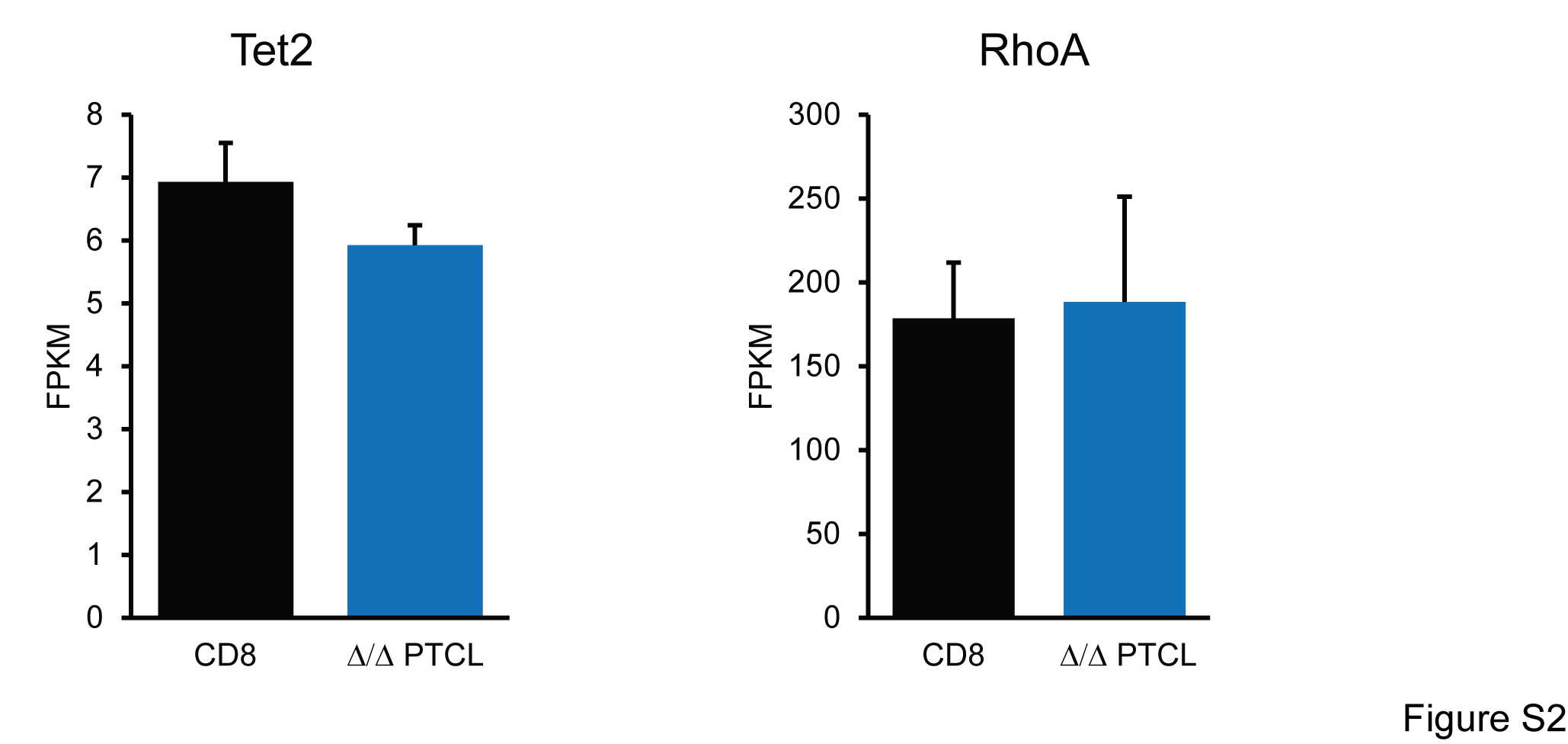

Supplement: S2 Fig — Expression data from RNAseq (FPKM) for Tet2 and RhoA transcript levels in Dnmt3a+/+ CD8+ T cells and Dnmt3aΔ/Δ PTCL samples. (TIF) [file pgen.1006334.s002.tif]

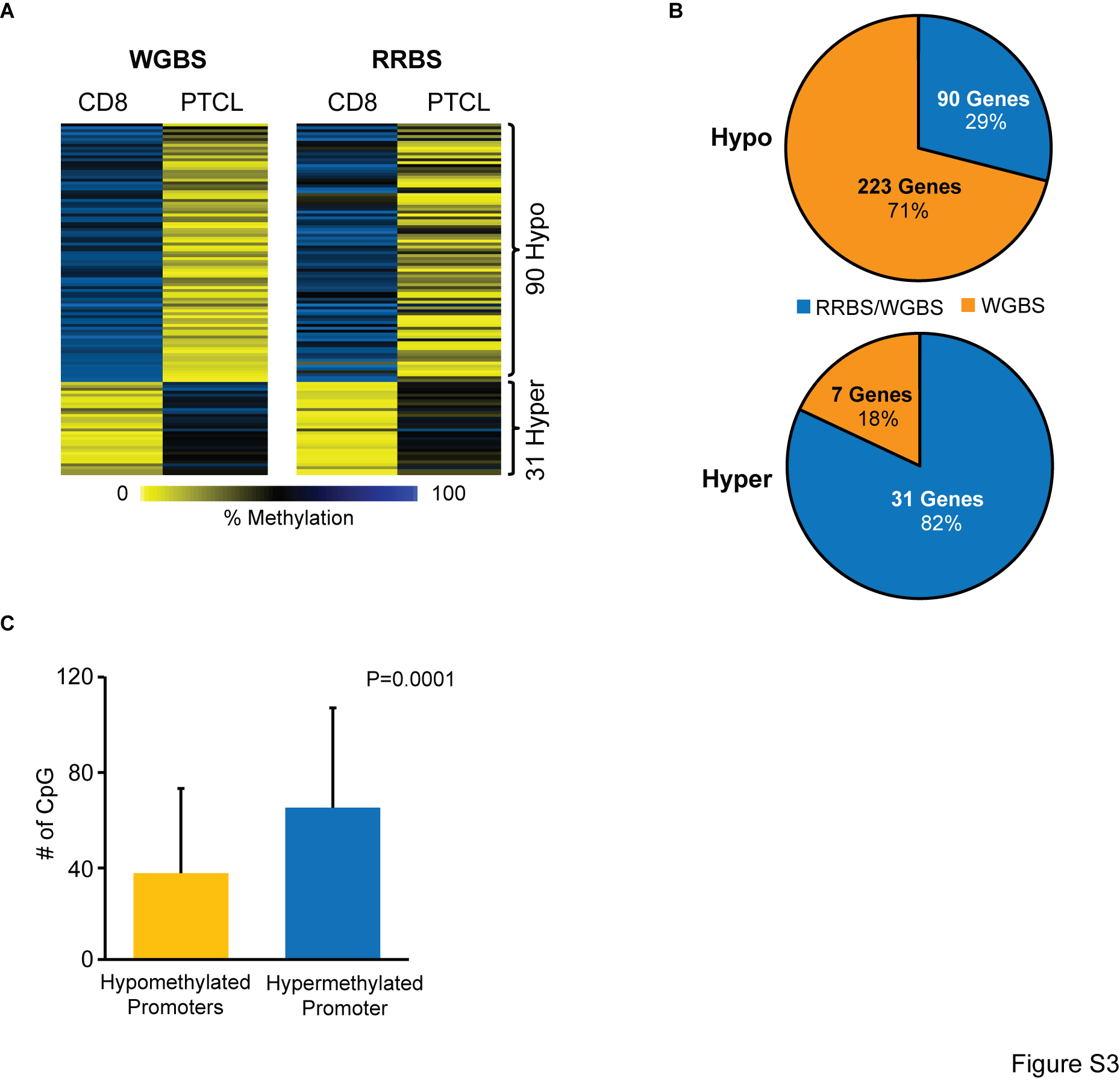

Supplement: S3 Fig — (A) Heat map displaying 90 hypomethylated and 31 hypermethylated promoters identified by WGBS and confirmed by RRBS. RRBS data is shown as the average percent methylation of DMCS annotated to long promoters (-1500 to +500 relative to TSS) for Dnmt3a+/+ CD8+ T cells (n = 2) and Dnmt3aΔ/Δ PTCL (n = 2). DMCS are defined by a ≥30% change in percent methylation in tumor samples compared to wild-type control samples. (B) RRBS confirmation of differentially methylated promoters identified by WGBS. Hypomethylated (top) and hypermethylated (bottom) genes confirmed by RRBS are shown in blue. Differentially methylated gene promoters identified by WGBS, but not confirmed RRBS are shown in orange. (C) The average number of CpG dinucleotides present in hypo- and hypermethylated promoter regions (-500 to +1500 bp relative to TSS) in Dnmt3aΔ/Δ PTCL, as compared to Dnmt3a+/+ CD8+ T cells. Error bars show standard deviation. (TIF) [file pgen.1006334.s003.tif]

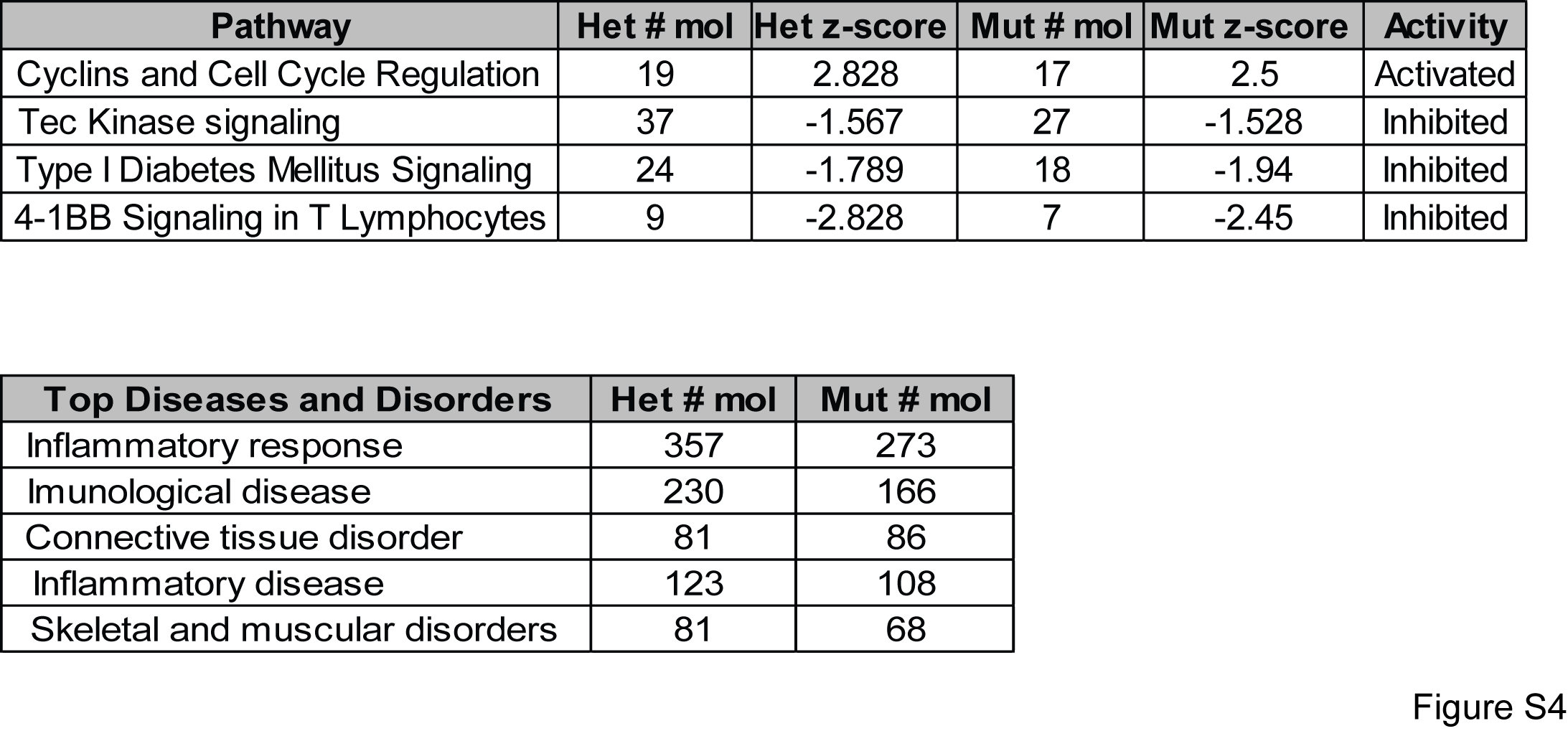

Supplement: S4 Fig — Summary of top categories, including “pathways” and “diseases and disorders”, derived from Ingenuity pathway analysis (IPA) of genes differentially expressed in both Dnmt3a+/- and Dnmt3aΔ/Δ PTCL relative to Dnmt3a+/+ CD8+ T cell controls. P<0.05 for all categories. (TIF) [file pgen.1006334.s004.tif]

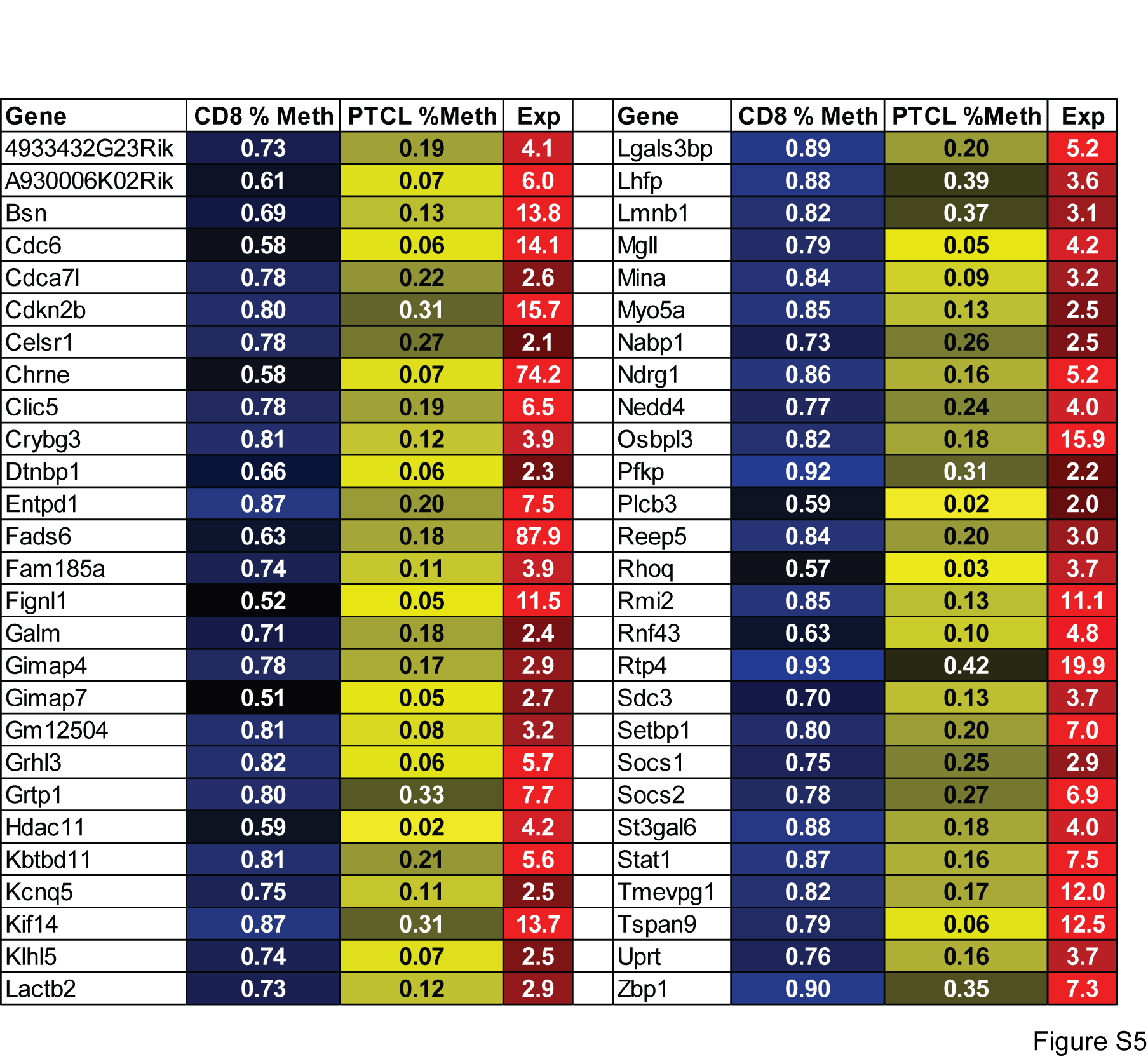

Supplement: S5 Fig — List of genes that are overexpressed and whose predicted enhancer regions are hypomethylated in Dnmt3aΔ/Δ PTCL relative to Dnmt3a+/+ CD8+ T cell controls. Percent methylation derived from WGBS for enhancer regions for Dnmt3a+/+ CD8+ T cells and Dnmt3aΔ/Δ PTCL is shown in blue (representing high levels of methylation) and yellow (representing low levels of methylation). Corresponding fold changes in gene expression (determined by RNA-seq) for Dnmt3aΔ/Δ PTCL relative to control Dnmt3a+/+ CD8+ samples are shown in red. (TIF) [file pgen.1006334.s005.tif]

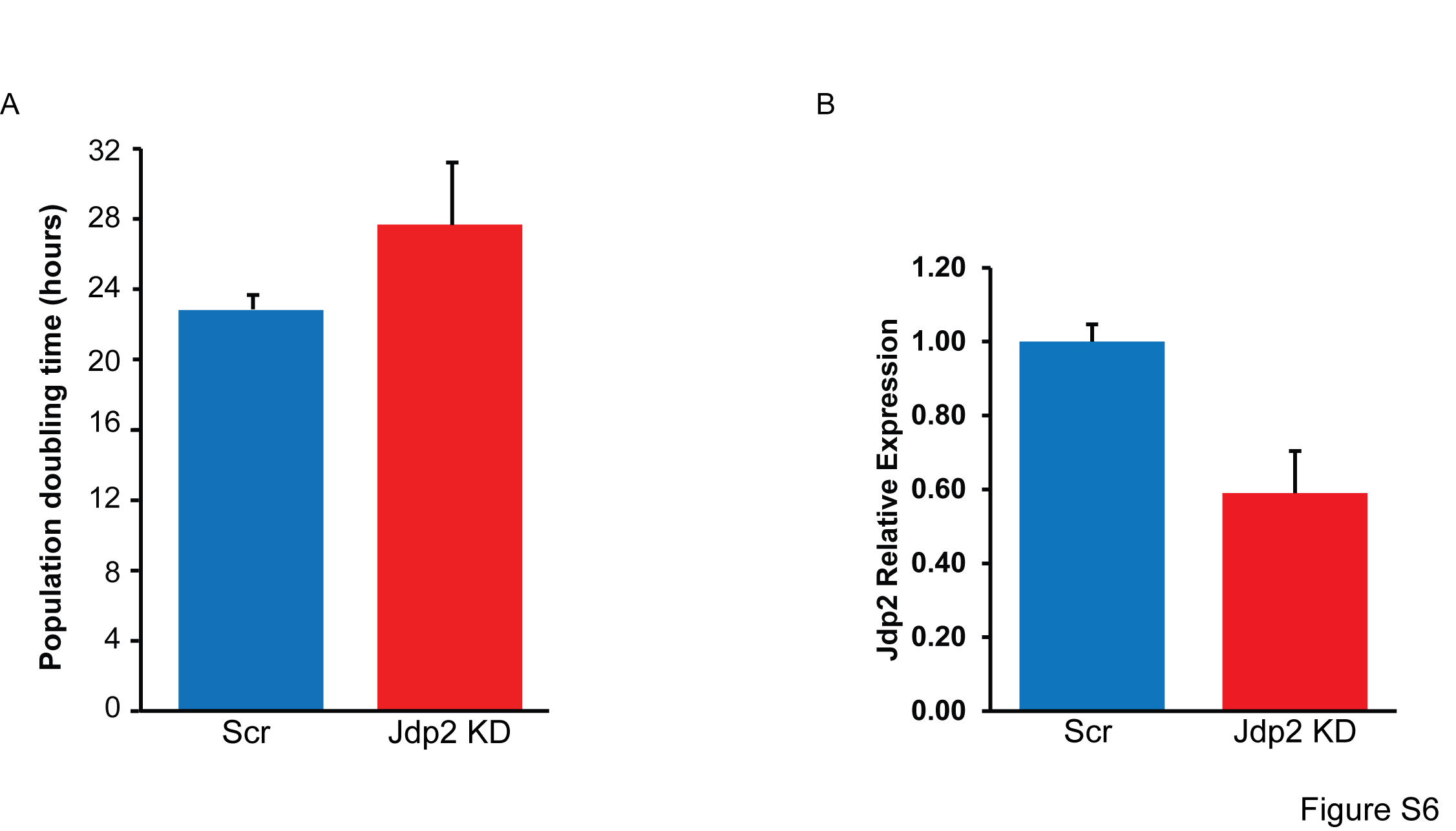

Supplement: S6 Fig — (A) Average population doubling time for a Dnmt3a-/- MYC-induced T cell lymphoma line infected with either scrambled shRNA (blue) or shRNA against Jdp2 (red). Error bars show standard deviation. (B) Normalized gene expression of Jdp2 transcript levels as determined by qRT-PCR for a Dnmt3a-/- MYC-induced T cell lymphoma line infected with either scrambled shRNA (blue) or shRNA against Jdp2 (red). Error bars show standard deviation. (TIF) [file pgen.1006334.s006.tif]

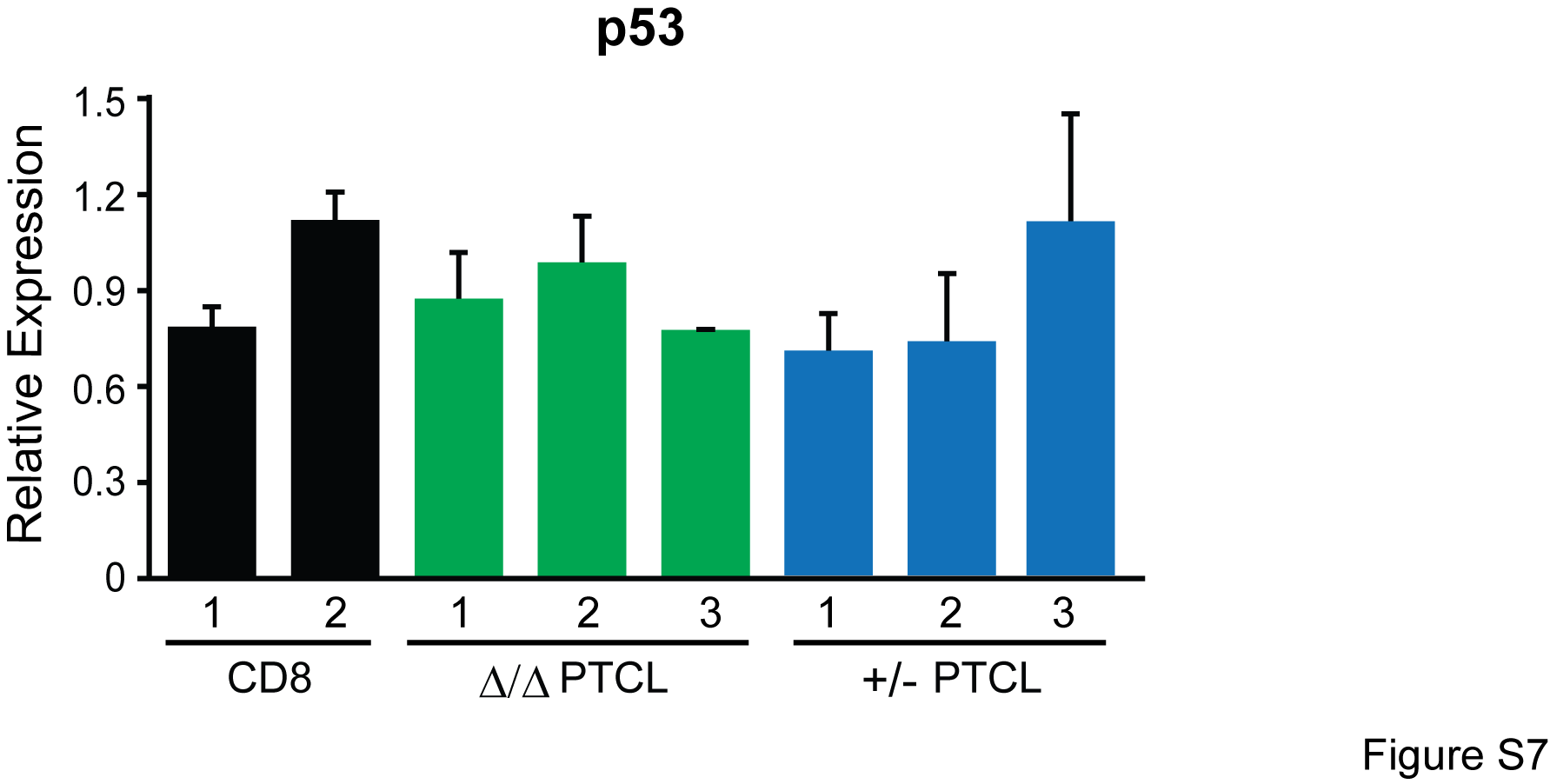

Supplement: S7 Fig — Normalized gene expression of Tp53 transcript levels as determined by qRT-PCR in mouse Dnmt3a+/+ CD8+ T cell control, Dnmt3a+/- PTCL, and Dnmt3aΔ/Δ PTCL samples. Data presented are the average of two independent experiments. Error bars show standard deviation. (TIF) [file pgen.1006334.s007.tif]

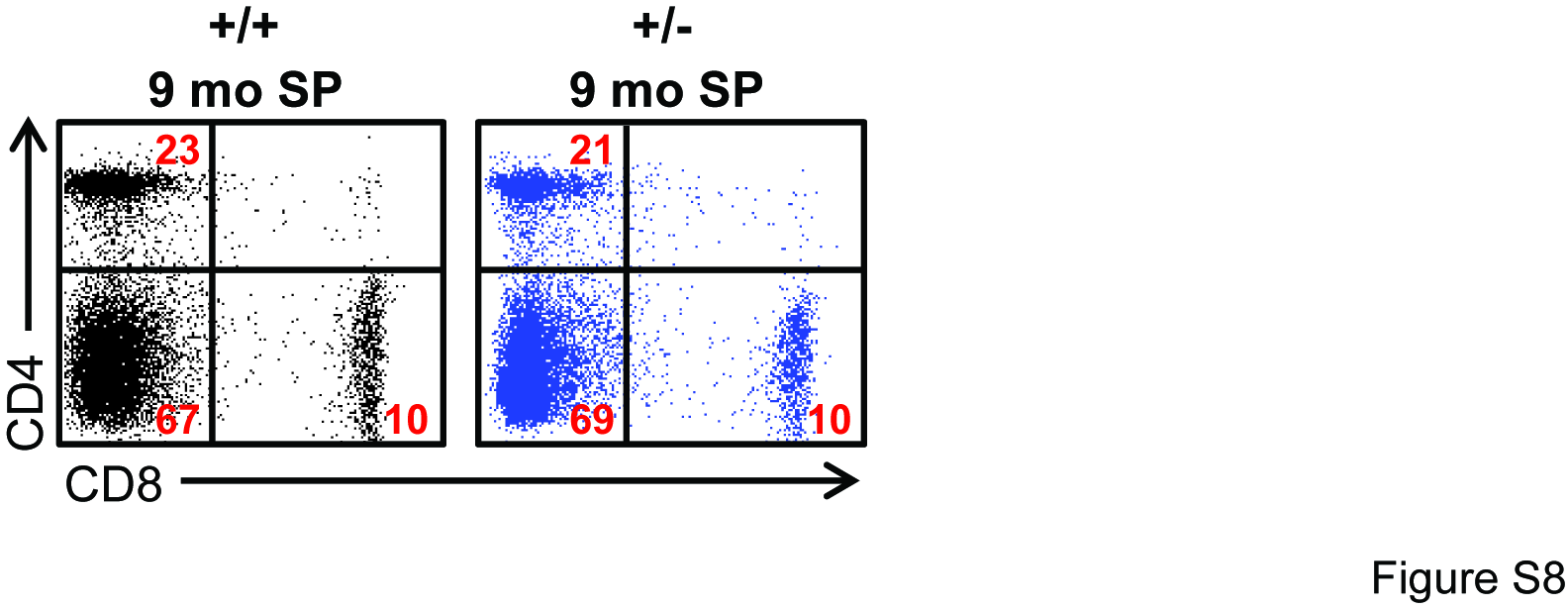

Supplement: S8 Fig — CD4 and CD8 expression in cells isolated from the spleen of 9 months old Dnmt3a+/+ (+/+) and Dnmt3a+/- (+/-) mice, as determined by FACS. Percentage of cells in each quadrant are shown in red. (TIF) [file pgen.1006334.s008.tif]
