# Supplementary material for: Dnmt3a Is a Haploinsufficient Tumor Suppressor in CD8+ Peripheral T Cell Lymphoma
Source: PLoS Genet. 2016 Sep 30;12(9):e1006334. doi: 10.1371/journal.pgen.1006334 (PMC5045215; doi:10.1371/journal.pgen.1006334)
Supplement: S1 File — Sequencing results for the coding domain sequence for Dnmt3a CDS in one wild-type CD8+ sample and two Dnmt3a+/- PTCL samples. (DOCX) [file pgen.1006334.s020.docx]

**Supplemental File S1:**

**Sequencing of Dnmt3a Wild-Type transcript cDNA coding domain sequence in mouse Dnmt3a(+/-) PTCL shows no mutations**

[Dnmt3a]_Dnmt3a(+/+)_WT_CD8_Reference atgccctccagcggccccggggacaccagcagctcctctctggagcgggaggatgatcga 60

[Dnmt3a]_Dnmt3a(+/-)_PTCL_1_(AF(X)s8) atgccctccagcggccccggggacaccagcagctcctctctggagcgggaggatgatcga 60

[Dnmt3a]_Dnmt3a(+/-)_PTCL_2_(AF%(XI)x2) atgccctccagcggccccggggacaccagcagctcctctctggagcgggaggatgatcga 60

************************************************************

[Dnmt3a]_Dnmt3a(+/+)_WT_CD8_Reference aaggaaggagaggaacaggaggagaaccgtggcaaggaagagcgccaggagcccagcgcc 120

[Dnmt3a]_Dnmt3a(+/-)_PTCL_1_(AF(X)s8) aaggaaggagaggaacaggaggagaaccgtggcaaggaagagcgccaggagcccagcgcc 120

[Dnmt3a]_Dnmt3a(+/-)_PTCL_2_(AF%(XI)x2) aaggaaggagaggaacaggaggagaaccgtggcaaggaagagcgccaggagcccagcgcc 120

************************************************************

[Dnmt3a]_Dnmt3a(+/+)_WT_CD8_Reference acggcccggaaggtggggaggcctggccggaagcgcaagcacccaccggtggaaagcagt 180

[Dnmt3a]_Dnmt3a(+/-)_PTCL_1_(AF(X)s8) acggcccggaaggtggggaggcctggccggaagcgcaagcacccaccggtggaaagcagt 180

[Dnmt3a]_Dnmt3a(+/-)_PTCL_2_(AF%(XI)x2) acggcccggaaggtggggaggcctggccggaagcgcaagcacccaccggtggaaagcagt 180

************************************************************

[Dnmt3a]_Dnmt3a(+/+)_WT_CD8_Reference gacacccccaaggacccagcagtgaccaccaagtctcagcccatggcccaggactctggc 240

[Dnmt3a]_Dnmt3a(+/-)_PTCL_1_(AF(X)s8) gacacccccaaggacccagcagtgaccaccaagtctcagcccatggcccaggactctggc 240

[Dnmt3a]_Dnmt3a(+/-)_PTCL_2_(AF%(XI)x2) gacacccccaaggacccagcagtgaccaccaagtctcagcccatggcccaggactctggc 240

************************************************************

[Dnmt3a]_Dnmt3a(+/+)_WT_CD8_Reference ccctcagatctgctacccaatggagacttggagaagcggagtgaaccccaacctgaggag 300

[Dnmt3a]_Dnmt3a(+/-)_PTCL_1_(AF(X)s8) ccctcagatctgctacccaatggagacttggagaagcggagtgaaccccaacctgaggag 300

[Dnmt3a]_Dnmt3a(+/-)_PTCL_2_(AF%(XI)x2) ccctcagatctgctacccaatggagacttggagaagcggagtgaaccccaacctgaggag 300

************************************************************

[Dnmt3a]_Dnmt3a(+/+)_WT_CD8_Reference gggagcccagctgcagggcagaagggtggggccccagctgaaggagagggaactgagacc 360

[Dnmt3a]_Dnmt3a(+/-)_PTCL_1_(AF(X)s8) gggagcccagctgcagggcagaagggtggggccccagctgaaggagagggaactgagacc 360

[Dnmt3a]_Dnmt3a(+/-)_PTCL_2_(AF%(XI)x2) gggagcccagctgcagggcagaagggtggggccccagctgaaggagagggaactgagacc 360

************************************************************

[Dnmt3a]_Dnmt3a(+/+)_WT_CD8_Reference ccaccagaagcctccagagctgtggagaatggctgctgtgtgaccaaggaaggccgtgga 420

[Dnmt3a]_Dnmt3a(+/-)_PTCL_1_(AF(X)s8) ccaccagaagcctccagagctgtggagaatggctgctgtgtgaccaaggaaggccgtgga 420

[Dnmt3a]_Dnmt3a(+/-)_PTCL_2_(AF%(XI)x2) ccaccagaagcctccagagctgtggagaatggctgctgtgtgaccaaggaaggccgtgga 420

************************************************************

[Dnmt3a]_Dnmt3a(+/+)_WT_CD8_Reference gcctctgcaggagagggcaaagaacagaagcagaccaacatcgaatccatgaaaatggag 480

[Dnmt3a]_Dnmt3a(+/-)_PTCL_1_(AF(X)s8) gcctctgcaggagagggcaaagaacagaagcagaccaacatcgaatccatgaaaatggag 480

[Dnmt3a]_Dnmt3a(+/-)_PTCL_2_(AF%(XI)x2) gcctctgcaggagagggcaaagaacagaagcagaccaacatcgaatccatgaaaatggag 480

************************************************************

[Dnmt3a]_Dnmt3a(+/+)_WT_CD8_Reference ggctcccggggccgactgcgaggtggcttgggctgggagtccagcctccgtcagcgaccc 540

[Dnmt3a]_Dnmt3a(+/-)_PTCL_1_(AF(X)s8) ggctcccggggccgactgcgaggtggcttgggctgggagtccagcctccgtcagcgaccc 540

[Dnmt3a]_Dnmt3a(+/-)_PTCL_2_(AF%(XI)x2) ggctcccggggccgactgcgaggtggcttgggctgggagtccagcctccgtcagcgaccc 540

************************************************************

[Dnmt3a]_Dnmt3a(+/+)_WT_CD8_Reference atgccaagactcaccttccaggcaggggacccctactacatcagcaaacggaaacgggat 600

[Dnmt3a]_Dnmt3a(+/-)_PTCL_1_(AF(X)s8) atgccaagactcaccttccaggcaggggacccctactacatcagcaaacggaaacgggat 600

[Dnmt3a]_Dnmt3a(+/-)_PTCL_2_(AF%(XI)x2) atgccaagactcaccttccaggcaggggacccctactacatcagcaaacggaaacgggat 600

************************************************************

[Dnmt3a]_Dnmt3a(+/+)_WT_CD8_Reference gagtggctggcacgttggaaaagggaggctgagaagaaagccaaggtaattgcagtaatg 660

[Dnmt3a]_Dnmt3a(+/-)_PTCL_1_(AF(X)s8) gagtggctggcacgttggaaaagggaggctgagaagaaagccaaggtaattgcagtaatg 660

[Dnmt3a]_Dnmt3a(+/-)_PTCL_2_(AF%(XI)x2) gagtggctggcacgttggaaaagggaggctgagaagaaagccaaggtaattgcagtaatg 660

************************************************************

[Dnmt3a]_Dnmt3a(+/+)_WT_CD8_Reference aatgctgtggaagagaaccaggcctctggagagtctcagaaggtggaggaggccagccct 720

[Dnmt3a]_Dnmt3a(+/-)_PTCL_1_(AF(X)s8) aatgctgtggaagagaaccaggcctctggagagtctcagaaggtggaggaggccagccct 720

[Dnmt3a]_Dnmt3a(+/-)_PTCL_2_(AF%(XI)x2) aatgctgtggaagagaaccaggcctctggagagtctcagaaggtggaggaggccagccct 720

************************************************************

[Dnmt3a]_Dnmt3a(+/+)_WT_CD8_Reference cctgctgtgcagcagcccacggaccctgcttctccgactgtggccaccacccctgagcca 780

[Dnmt3a]_Dnmt3a(+/-)_PTCL_1_(AF(X)s8) cctgctgtgcagcagcccacggaccctgcttctccgactgtggccaccacccctgagcca 780

[Dnmt3a]_Dnmt3a(+/-)_PTCL_2_(AF%(XI)x2) cctgctgtgcagcagcccacggaccctgcttctccgactgtggccaccacccctgagcca 780

************************************************************

[Dnmt3a]_Dnmt3a(+/+)_WT_CD8_Reference gtaggaggggatgctggggacaagaatgctaccaaagcagccgacgatgagcctgagtat 840

[Dnmt3a]_Dnmt3a(+/-)_PTCL_1_(AF(X)s8) gtaggaggggatgctggggacaagaatgctaccaaagcagccgacgatgagcctgagtat 840

[Dnmt3a]_Dnmt3a(+/-)_PTCL_2_(AF%(XI)x2) gtaggaggggatgctggggacaagaatgctaccaaagcagccgacgatgagcctgagtat 840

************************************************************

[Dnmt3a]_Dnmt3a(+/+)_WT_CD8_Reference gaggatggccggggctttggcattggagagctggtgtgggggaaacttcggggcttctcc 900

[Dnmt3a]_Dnmt3a(+/-)_PTCL_1_(AF(X)s8) gaggatggccggggctttggcattggagagctggtgtgggggaaacttcggggcttctcc 900

[Dnmt3a]_Dnmt3a(+/-)_PTCL_2_(AF%(XI)x2) gaggatggccggggctttggcattggagagctggtgtgggggaaacttcggggcttctcc 900

************************************************************

[Dnmt3a]_Dnmt3a(+/+)_WT_CD8_Reference tggtggccaggccgaattgtgtcttggtggatgacaggccggagccgagcagctgaaggc 960

[Dnmt3a]_Dnmt3a(+/-)_PTCL_1_(AF(X)s8) tggtggccaggccgaattgtgtcttggtggatgacaggccggagccgagcagctgaaggc 960

[Dnmt3a]_Dnmt3a(+/-)_PTCL_2_(AF%(XI)x2) tggtggccaggccgaattgtgtcttggtggatgacaggccggagccgagcagctgaaggc 960

************************************************************

[Dnmt3a]_Dnmt3a(+/+)_WT_CD8_Reference actcgctgggtcatgtggttcggagatggcaagttctcagtggtgtgtgtggagaagctc 1020

[Dnmt3a]_Dnmt3a(+/-)_PTCL_1_(AF(X)s8) actcgctgggtcatgtggttcggagatggcaagttctcagtggtgtgtgtggagaagctc 1020

[Dnmt3a]_Dnmt3a(+/-)_PTCL_2_(AF%(XI)x2) actcgctgggtcatgtggttcggagatggcaagttctcagtggtgtgtgtggagaagctc 1020

************************************************************

[Dnmt3a]_Dnmt3a(+/+)_WT_CD8_Reference atgccgctgagctccttctgcagtgcattccaccaggccacctacaacaagcagcccatg 1080

[Dnmt3a]_Dnmt3a(+/-)_PTCL_1_(AF(X)s8) atgccgctgagctccttctgcagtgcattccaccaggccacctacaacaagcagcccatg 1080

[Dnmt3a]_Dnmt3a(+/-)_PTCL_2_(AF%(XI)x2) atgccgctgagctccttctgcagtgcattccaccaggccacctacaacaagcagcccatg 1080

************************************************************

[Dnmt3a]_Dnmt3a(+/+)_WT_CD8_Reference taccgcaaagccatctacgaagtcctccaggtggccagcagccgtgccgggaagctgttt 1140

[Dnmt3a]_Dnmt3a(+/-)_PTCL_1_(AF(X)s8) taccgcaaagccatctacgaagtcctccaggtggccagcagccgtgccgggaagctgttt 1140

[Dnmt3a]_Dnmt3a(+/-)_PTCL_2_(AF%(XI)x2) taccgcaaagccatctacgaagtcctccaggtggccagcagccgtgccgggaagctgttt 1140

************************************************************

[Dnmt3a]_Dnmt3a(+/+)_WT_CD8_Reference ccagcttgccatgacagtgatgaaagtgacagtggcaaggctgtggaagtgcagaacaag 1200

[Dnmt3a]_Dnmt3a(+/-)_PTCL_1_(AF(X)s8) ccagcttgccatgacagtgatgaaagtgacagtggcaaggctgtggaagtgcagaacaag 1200

[Dnmt3a]_Dnmt3a(+/-)_PTCL_2_(AF%(XI)x2) ccagcttgccatgacagtgatgaaagtgacagtggcaaggctgtggaagtgcagaacaag 1200

************************************************************

[Dnmt3a]_Dnmt3a(+/+)_WT_CD8_Reference cagatgattgaatgggccctcggtggcttccagccctcgggtcctaagggcctggagcca 1260

[Dnmt3a]_Dnmt3a(+/-)_PTCL_1_(AF(X)s8) cagatgattgaatgggccctcggtggcttccagccctcgggtcctaagggcctggagcca 1260

[Dnmt3a]_Dnmt3a(+/-)_PTCL_2_(AF%(XI)x2) cagatgattgaatgggccctcggtggcttccagccctcgggtcctaagggcctggagcca 1260

************************************************************

[Dnmt3a]_Dnmt3a(+/+)_WT_CD8_Reference ccagaagaagagaagaatccttacaaggaagtttacaccgacatgtgggtggagcctgaa 1320

[Dnmt3a]_Dnmt3a(+/-)_PTCL_1_(AF(X)s8) ccagaagaagagaagaatccttacaaggaagtttacaccgacatgtgggtggagcctgaa 1320

[Dnmt3a]_Dnmt3a(+/-)_PTCL_2_(AF%(XI)x2) ccagaagaagagaagaatccttacaaggaagtttacaccgacatgtgggtggagcctgaa 1320

************************************************************

[Dnmt3a]_Dnmt3a(+/+)_WT_CD8_Reference gcagctgcttacgccccacccccaccagccaagaaacccagaaagagcacaacagagaaa 1380

[Dnmt3a]_Dnmt3a(+/-)_PTCL_1_(AF(X)s8) gcagctgcttacgccccacccccaccagccaagaaacccagaaagagcacaacagagaaa 1380

[Dnmt3a]_Dnmt3a(+/-)_PTCL_2_(AF%(XI)x2) gcagctgcttacgccccacccccaccagccaagaaacccagaaagagcacaacagagaaa 1380

************************************************************

[Dnmt3a]_Dnmt3a(+/+)_WT_CD8_Reference cctaaggtcaaggagatcattgatgagcgcacaagggagcggctggtgtatgaggtgcgc 1440

[Dnmt3a]_Dnmt3a(+/-)_PTCL_1_(AF(X)s8) cctaaggtcaaggagatcattgatgagcgcacaagggagcggctggtgtatgaggtgcgc 1440

[Dnmt3a]_Dnmt3a(+/-)_PTCL_2_(AF%(XI)x2) cctaaggtcaaggagatcattgatgagcgcacaagggagcggctggtgtatgaggtgcgc 1440

************************************************************

[Dnmt3a]_Dnmt3a(+/+)_WT_CD8_Reference cagaagtgcagaaacatcgaggacatttgtatctcatgtgggagcctcaatgtcaccctg 1500

[Dnmt3a]_Dnmt3a(+/-)_PTCL_1_(AF(X)s8) cagaagtgcagaaacatcgaggacatttgtatctcatgtgggagcctcaatgtcaccctg 1500

[Dnmt3a]_Dnmt3a(+/-)_PTCL_2_(AF%(XI)x2) cagaagtgcagaaacatcgaggacatttgtatctcatgtgggagcctcaatgtcaccctg 1500

************************************************************

[Dnmt3a]_Dnmt3a(+/+)_WT_CD8_Reference gagcacccactcttcattggaggcatgtgccagaactgtaagaactgcttcttggagtgt 1560

[Dnmt3a]_Dnmt3a(+/-)_PTCL_1_(AF(X)s8) gagcacccactcttcattggaggcatgtgccagaactgtaagaactgcttcttggagtgt 1560

[Dnmt3a]_Dnmt3a(+/-)_PTCL_2_(AF%(XI)x2) gagcacccactcttcattggaggcatgtgccagaactgtaagaactgcttcttggagtgt 1560

************************************************************

[Dnmt3a]_Dnmt3a(+/+)_WT_CD8_Reference gcttaccagtatgacgacgatgggtaccagtcctattgcaccatctgctgtggggggcgt 1620

[Dnmt3a]_Dnmt3a(+/-)_PTCL_1_(AF(X)s8) gcttaccagtatgacgacgatgggtaccagtcctattgcaccatctgctgtggggggcgt 1620

[Dnmt3a]_Dnmt3a(+/-)_PTCL_2_(AF%(XI)x2) gcttaccagtatgacgacgatgggtaccagtcctattgcaccatctgctgtggggggcgt 1620

************************************************************

[Dnmt3a]_Dnmt3a(+/+)_WT_CD8_Reference gaagtgctcatgtgtgggaacaacaactgctgcaggtgcttttgtgtcgagtgtgtggat 1680

[Dnmt3a]_Dnmt3a(+/-)_PTCL_1_(AF(X)s8) gaagtgctcatgtgtgggaacaacaactgctgcaggtgcttttgtgtcgagtgtgtggat 1680

[Dnmt3a]_Dnmt3a(+/-)_PTCL_2_(AF%(XI)x2) gaagtgctcatgtgtgggaacaacaactgctgcaggtgcttttgtgtcgagtgtgtggat 1680

************************************************************

[Dnmt3a]_Dnmt3a(+/+)_WT_CD8_Reference ctcttggtggggccaggagctgctcaggcagccattaaggaagacccctggaactgctac 1740

[Dnmt3a]_Dnmt3a(+/-)_PTCL_1_(AF(X)s8) ctcttggtggggccaggagctgctcaggcagccattaaggaagacccctggaactgctac 1740

[Dnmt3a]_Dnmt3a(+/-)_PTCL_2_(AF%(XI)x2) ctcttggtggggccaggagctgctcaggcagccattaaggaagacccctggaactgctac 1740

************************************************************

[Dnmt3a]_Dnmt3a(+/+)_WT_CD8_Reference atgtgcgggcataagggcacctatgggctgctgcgaagacgggaagactggccttctcga 1800

[Dnmt3a]_Dnmt3a(+/-)_PTCL_1_(AF(X)s8) atgtgcgggcataagggcacctatgggctgctgcgaagacgggaagactggccttctcga 1800

[Dnmt3a]_Dnmt3a(+/-)_PTCL_2_(AF%(XI)x2) atgtgcgggcataagggcacctatgggctgctgcgaagacgggaagactggccttctcga 1800

************************************************************

[Dnmt3a]_Dnmt3a(+/+)_WT_CD8_Reference ctccagatgttctttgccaataaccatgaccaggaatttgaccccccaaaggtttaccca 1860

[Dnmt3a]_Dnmt3a(+/-)_PTCL_1_(AF(X)s8) ctccagatgttctttgccaataaccatgaccaggaatttgaccccccaaaggtttaccca 1860

[Dnmt3a]_Dnmt3a(+/-)_PTCL_2_(AF%(XI)x2) ctccagatgttctttgccaataaccatgaccaggaatttgaccccccaaaggtttaccca 1860

************************************************************

[Dnmt3a]_Dnmt3a(+/+)_WT_CD8_Reference cctgtgccagctgagaagaggaagcccatccgcgtgctgtctctctttgatgggattgct 1920

[Dnmt3a]_Dnmt3a(+/-)_PTCL_1_(AF(X)s8) cctgtgccagctgagaagaggaagcccatccgcgtgctgtctctctttgatgggattgct 1920

[Dnmt3a]_Dnmt3a(+/-)_PTCL_2_(AF%(XI)x2) cctgtgccagctgagaagaggaagcccatccgcgtgctgtctctctttgatgggattgct 1920

************************************************************

[Dnmt3a]_Dnmt3a(+/+)_WT_CD8_Reference acagggctcctggtgctgaaggacctgggcatccaagtggaccgctacattgcctccgag 1980

[Dnmt3a]_Dnmt3a(+/-)_PTCL_1_(AF(X)s8) acagggctcctggtgctgaaggacctgggcatccaagtggaccgctacattgcctccgag 1980

[Dnmt3a]_Dnmt3a(+/-)_PTCL_2_(AF%(XI)x2) acagggctcctggtgctgaaggacctgggcatccaagtggaccgctacattgcctccgag 1980

************************************************************

[Dnmt3a]_Dnmt3a(+/+)_WT_CD8_Reference gtgtgtgaggactccatcacggtgggcatggtgcggcaccagggaaagatcatgtacgtc 2040

[Dnmt3a]_Dnmt3a(+/-)_PTCL_1_(AF(X)s8) gtgtgtgaggactccatcacggtgggcatggtgcggcaccagggaaagatcatgtacgtc 2040

[Dnmt3a]_Dnmt3a(+/-)_PTCL_2_(AF%(XI)x2) gtgtgtgaggactccatcacggtgggcatggtgcggcaccagggaaagatcatgtacgtc 2040

************************************************************

[Dnmt3a]_Dnmt3a(+/+)_WT_CD8_Reference ggggacgtccgcagcgtcacacagaagcatatccaggagtggggcccattcgacctggtg 2100

[Dnmt3a]_Dnmt3a(+/-)_PTCL_1_(AF(X)s8) ggggacgtccgcagcgtcacacagaagcatatccaggagtggggcccattcgacctggtg 2100

[Dnmt3a]_Dnmt3a(+/-)_PTCL_2_(AF%(XI)x2) ggggacgtccgcagcgtcacacagaagcatatccaggagtggggcccattcgacctggtg 2100

************************************************************

[Dnmt3a]_Dnmt3a(+/+)_WT_CD8_Reference attggaggcagtccctgcaatgacctctccattgtcaaccctgcccgcaagggactttat 2160

[Dnmt3a]_Dnmt3a(+/-)_PTCL_1_(AF(X)s8) attggaggcagtccctgcaatgacctctccattgtcaaccctgcccgcaagggactttat 2160

[Dnmt3a]_Dnmt3a(+/-)_PTCL_2_(AF%(XI)x2) attggaggcagtccctgcaatgacctctccattgtcaaccctgcccgcaagggactttat 2160

************************************************************

[Dnmt3a]_Dnmt3a(+/+)_WT_CD8_Reference gagggtactggccgcctcttctttgagttctaccgcctcctgcatgatgcgcggcccaag 2220

[Dnmt3a]_Dnmt3a(+/-)_PTCL_1_(AF(X)s8) gagggtactggccgcctcttctttgagttctaccgcctcctgcatgatgcgcggcccaag 2220

[Dnmt3a]_Dnmt3a(+/-)_PTCL_2_(AF%(XI)x2) gagggtactggccgcctcttctttgagttctaccgcctcctgcatgatgcgcggcccaag 2220

************************************************************

[Dnmt3a]_Dnmt3a(+/+)_WT_CD8_Reference gagggagatgatcgccccttcttctggctctttgagaatgtggtggccatgggcgttagt 2280

[Dnmt3a]_Dnmt3a(+/-)_PTCL_1_(AF(X)s8) gagggagatgatcgccccttcttctggctctttgagaatgtggtggccatgggcgttagt 2280

[Dnmt3a]_Dnmt3a(+/-)_PTCL_2_(AF%(XI)x2) gagggagatgatcgccccttcttctggctctttgagaatgtggtggccatgggcgttagt 2280

************************************************************

[Dnmt3a]_Dnmt3a(+/+)_WT_CD8_Reference gacaagagggacatctcgcgatttcttgagtctaaccccgtgatgattgacgccaaagaa 2340

[Dnmt3a]_Dnmt3a(+/-)_PTCL_1_(AF(X)s8) gacaagagggacatctcgcgatttcttgagtctaaccccgtgatgattgacgccaaagaa 2340

[Dnmt3a]_Dnmt3a(+/-)_PTCL_2_(AF%(XI)x2) gacaagagggacatctcgcgatttcttgagtctaaccccgtgatgattgacgccaaagaa 2340

************************************************************

[Dnmt3a]_Dnmt3a(+/+)_WT_CD8_Reference gtgtctgctgcacacagggcccgttacttctggggtaaccttcctggcatgaacaggcct 2400

[Dnmt3a]_Dnmt3a(+/-)_PTCL_1_(AF(X)s8) gtgtctgctgcacacagggcccgttacttctggggtaaccttcctggcatgaacaggcct 2400

[Dnmt3a]_Dnmt3a(+/-)_PTCL_2_(AF%(XI)x2) gtgtctgctgcacacagggcccgttacttctggggtaaccttcctggcatgaacaggcct 2400

************************************************************

[Dnmt3a]_Dnmt3a(+/+)_WT_CD8_Reference ttggcatccactgtgaatgataagctggagctgcaagagtgtctggagcacggcagaata 2460

[Dnmt3a]_Dnmt3a(+/-)_PTCL_1_(AF(X)s8) ttggcatccactgtgaatgataagctggagctgcaagagtgtctggagcacggcagaata 2460

[Dnmt3a]_Dnmt3a(+/-)_PTCL_2_(AF%(XI)x2) ttggcatccactgtgaatgataagctggagctgcaagagtgtctggagcacggcagaata 2460

************************************************************

[Dnmt3a]_Dnmt3a(+/+)_WT_CD8_Reference gccaagttcagcaaagtgaggaccattaccaccaggtcaaactctataaagcagggcaaa 2520

[Dnmt3a]_Dnmt3a(+/-)_PTCL_1_(AF(X)s8) gccaagttcagcaaagtgaggaccattaccaccaggtcaaactctataaagcagggcaaa 2520

[Dnmt3a]_Dnmt3a(+/-)_PTCL_2_(AF%(XI)x2) gccaagttcagcaaagtgaggaccattaccaccaggtcaaactctataaagcagggcaaa 2520

************************************************************

[Dnmt3a]_Dnmt3a(+/+)_WT_CD8_Reference gaccagcatttccccgtcttcatgaacgagaaggaggacatcctgtggtgcactgaaatg 2580

[Dnmt3a]_Dnmt3a(+/-)_PTCL_1_(AF(X)s8) gaccagcatttccccgtcttcatgaacgagaaggaggacatcctgtggtgcactgaaatg 2580

[Dnmt3a]_Dnmt3a(+/-)_PTCL_2_(AF%(XI)x2) gaccagcatttccccgtcttcatgaacgagaaggaggacatcctgtggtgcactgaaatg 2580

************************************************************

[Dnmt3a]_Dnmt3a(+/+)_WT_CD8_Reference gaaagggtgtttggcttccccgtccactacacagacgtctccaacatgagccgcttggcg 2640

[Dnmt3a]_Dnmt3a(+/-)_PTCL_1_(AF(X)s8) gaaagggtgtttggcttccccgtccactacacagacgtctccaacatgagccgcttggcg 2640

[Dnmt3a]_Dnmt3a(+/-)_PTCL_2_(AF%(XI)x2) gaaagggtgtttggcttccccgtccactacacagacgtctccaacatgagccgcttggcg 2640

************************************************************

[Dnmt3a]_Dnmt3a(+/+)_WT_CD8_Reference aggcagagactgctgggccgatcgtggagcgtgccggtcatccgccacctcttcgctccg 2700

[Dnmt3a]_Dnmt3a(+/-)_PTCL_1_(AF(X)s8) aggcagagactgctgggccgatcgtggagcgtgccggtcatccgccacctcttcgctccg 2700

[Dnmt3a]_Dnmt3a(+/-)_PTCL_2_(AF%(XI)x2) aggcagagactgctgggccgatcgtggagcgtgccggtcatccgccacctcttcgctccg 2700

************************************************************

[Dnmt3a]_Dnmt3a(+/+)_WT_CD8_Reference ctgaaggaatattttgcttgtgtgtaa 2727

[Dnmt3a]_Dnmt3a(+/-)_PTCL_1_(AF(X)s8) ctgaaggaatattttgcttgtgtgtaa 2727

[Dnmt3a]_Dnmt3a(+/-)_PTCL_2_(AF%(XI)x2) ctgaaggaatattttgcttgtgtgtaa 2727

***************************
