# Supplementary material for: Dnmt3a Is a Haploinsufficient Tumor Suppressor in CD8+ Peripheral T Cell Lymphoma
Source: PLoS Genet. 2016 Sep 30;12(9):e1006334. doi: 10.1371/journal.pgen.1006334 (PMC5045215; doi:10.1371/journal.pgen.1006334)
Supplement: S2 File — Sequencing results for the coding domain sequence of RhoA and Tet2 in one wild-type CD8+ sample, three Dnmt3a+/- PTCL samples, and three Dnmt3aΔ/Δ PTCL samples. (DOCX) [file pgen.1006334.s021.docx]

**Supplemental File S2:**

**Sequencing of RhoA transcript cDNA coding domain sequence in mouse Dnmt3a(+/-) and Dnmt3a(Δ/Δ) PTCL shows no mutations**

[RhoA]_Dnmt3a(+/+)_WT_CD8_Reference atggctgccatcaggaagaaactggtgattgttggtgatggagcttgtggtaagacatgc 60

[RhoA]_Dnmt3a(+/-)_PTCL_1_(AF(X)s8) atggctgccatcaggaagaaactggtgattgttggtgatggagcttgtggtaagacatgc 60

[RhoA]_Dnmt3a(+/-)_PTCL_2_(AF%(XI)x2) atggctgccatcaggaagaaactggtgattgttggtgatggagcttgtggtaagacatgc 60

[RhoA]_Dnmt3a(+/-)_PTCL_3_(AF(IX)i1) atggctgccatcaggaagaaactggtgattgttggtgatggagcttgtggtaagacatgc 60

[RhoA]_Dnmt3a(-/-)_PTCL_1_(AF(IX)d4) atggctgccatcaggaagaaactggtgattgttggtgatggagcttgtggtaagacatgc 60

[RhoA]_Dnmt3a(-/-)_PTCL_2_(AF(IX)i4) atggctgccatcaggaagaaactggtgattgttggtgatggagcttgtggtaagacatgc 60

[RhoA]_Dnmt3a(-/-)_PTCL_3_(AF(IX)q3) atggctgccatcaggaagaaactggtgattgttggtgatggagcttgtggtaagacatgc 60

************************************************************

[RhoA]_Dnmt3a(+/+)_WT_CD8_Reference ttgctcatagtcttcagcaaggaccagttcccagaggtctatgtgcccacggtgtttgaa 120

[RhoA]_Dnmt3a(+/-)_PTCL_1_(AF(X)s8) ttgctcatagtcttcagcaaggaccagttcccagaggtctatgtgcccacggtgtttgaa 120

[RhoA]_Dnmt3a(+/-)_PTCL_2_(AF%(XI)x2) ttgctcatagtcttcagcaaggaccagttcccagaggtctatgtgcccacggtgtttgaa 120

[RhoA]_Dnmt3a(+/-)_PTCL_3_(AF(IX)i1) ttgctcatagtcttcagcaaggaccagttcccagaggtctatgtgcccacggtgtttgaa 120

[RhoA]_Dnmt3a(-/-)_PTCL_1_(AF(IX)d4) ttgctcatagtcttcagcaaggaccagttcccagaggtctatgtgcccacggtgtttgaa 120

[RhoA]_Dnmt3a(-/-)_PTCL_2_(AF(IX)i4) ttgctcatagtcttcagcaaggaccagttcccagaggtctatgtgcccacggtgtttgaa 120

[RhoA]_Dnmt3a(-/-)_PTCL_3_(AF(IX)q3) ttgctcatagtcttcagcaaggaccagttcccagaggtctatgtgcccacggtgtttgaa 120

************************************************************

[RhoA]_Dnmt3a(+/+)_WT_CD8_Reference aactatgtggcggatatcgaggtggatgggaagcaggtagagttggctttatgggacaca 180

[RhoA]_Dnmt3a(+/-)_PTCL_1_(AF(X)s8) aactatgtggcggatatcgaggtggatgggaagcaggtagagttggctttatgggacaca 180

[RhoA]_Dnmt3a(+/-)_PTCL_2_(AF%(XI)x2) aactatgtggcggatatcgaggtggatgggaagcaggtagagttggctttatgggacaca 180

[RhoA]_Dnmt3a(+/-)_PTCL_3_(AF(IX)i1) aactatgtggcggatatcgaggtggatgggaagcaggtagagttggctttatgggacaca 180

[RhoA]_Dnmt3a(-/-)_PTCL_1_(AF(IX)d4) aactatgtggcggatatcgaggtggatgggaagcaggtagagttggctttatgggacaca 180

[RhoA]_Dnmt3a(-/-)_PTCL_2_(AF(IX)i4) aactatgtggcggatatcgaggtggatgggaagcaggtagagttggctttatgggacaca 180

[RhoA]_Dnmt3a(-/-)_PTCL_3_(AF(IX)q3) aactatgtggcggatatcgaggtggatgggaagcaggtagagttggctttatgggacaca 180

************************************************************

[RhoA]_Dnmt3a(+/+)_WT_CD8_Reference gctggacaggaagattatgaccgcctgcggcctctctcttatccagacaccgatgttata 240

[RhoA]_Dnmt3a(+/-)_PTCL_1_(AF(X)s8) gctggacaggaagattatgaccgcctgcggcctctctcttatccagacaccgatgttata 240

[RhoA]_Dnmt3a(+/-)_PTCL_2_(AF%(XI)x2) gctggacaggaagattatgaccgcctgcggcctctctcttatccagacaccgatgttata 240

[RhoA]_Dnmt3a(+/-)_PTCL_3_(AF(IX)i1) gctggacaggaagattatgaccgcctgcggcctctctcttatccagacaccgatgttata 240

[RhoA]_Dnmt3a(-/-)_PTCL_1_(AF(IX)d4) gctggacaggaagattatgaccgcctgcggcctctctcttatccagacaccgatgttata 240

[RhoA]_Dnmt3a(-/-)_PTCL_2_(AF(IX)i4) gctggacaggaagattatgaccgcctgcggcctctctcttatccagacaccgatgttata 240

[RhoA]_Dnmt3a(-/-)_PTCL_3_(AF(IX)q3) gctggacaggaagattatgaccgcctgcggcctctctcttatccagacaccgatgttata 240

************************************************************

[RhoA]_Dnmt3a(+/+)_WT_CD8_Reference ttgatgtgtttttccattgacagccctgatagtttagaaaacatcccagaaaaatggact 300

[RhoA]_Dnmt3a(+/-)_PTCL_1_(AF(X)s8) ttgatgtgtttttccattgacagccctgatagtttagaaaacatcccagaaaaatggact 300

[RhoA]_Dnmt3a(+/-)_PTCL_2_(AF%(XI)x2) ttgatgtgtttttccattgacagccctgatagtttagaaaacatcccagaaaaatggact 300

[RhoA]_Dnmt3a(+/-)_PTCL_3_(AF(IX)i1) ttgatgtgtttttccattgacagccctgatagtttagaaaacatcccagaaaaatggact 300

[RhoA]_Dnmt3a(-/-)_PTCL_1_(AF(IX)d4) ttgatgtgtttttccattgacagccctgatagtttagaaaacatcccagaaaaatggact 300

[RhoA]_Dnmt3a(-/-)_PTCL_2_(AF(IX)i4) ttgatgtgtttttccattgacagccctgatagtttagaaaacatcccagaaaaatggact 300

[RhoA]_Dnmt3a(-/-)_PTCL_3_(AF(IX)q3) ttgatgtgtttttccattgacagccctgatagtttagaaaacatcccagaaaaatggact 300

************************************************************

[RhoA]_Dnmt3a(+/+)_WT_CD8_Reference ccagaagtcaagcatttctgtccaaatgtgcccatcatcctggttgggaacaagaaggac 360

[RhoA]_Dnmt3a(+/-)_PTCL_1_(AF(X)s8) ccagaagtcaagcatttctgtccaaatgtgcccatcatcctggttgggaacaagaaggac 360

[RhoA]_Dnmt3a(+/-)_PTCL_2_(AF%(XI)x2) ccagaagtcaagcatttctgtccaaatgtgcccatcatcctggttgggaacaagaaggac 360

[RhoA]_Dnmt3a(+/-)_PTCL_3_(AF(IX)i1) ccagaagtcaagcatttctgtccaaatgtgcccatcatcctggttgggaacaagaaggac 360

[RhoA]_Dnmt3a(-/-)_PTCL_1_(AF(IX)d4) ccagaagtcaagcatttctgtccaaatgtgcccatcatcctggttgggaacaagaaggac 360

[RhoA]_Dnmt3a(-/-)_PTCL_2_(AF(IX)i4) ccagaagtcaagcatttctgtccaaatgtgcccatcatcctggttgggaacaagaaggac 360

[RhoA]_Dnmt3a(-/-)_PTCL_3_(AF(IX)q3) ccagaagtcaagcatttctgtccaaatgtgcccatcatcctggttgggaacaagaaggac 360

************************************************************

[RhoA]_Dnmt3a(+/+)_WT_CD8_Reference cttcggaatgacgagcacacgagacgggagttggccaaaatgaagcaggagccggtaaaa 420

[RhoA]_Dnmt3a(+/-)_PTCL_1_(AF(X)s8) cttcggaatgacgagcacacgagacgggagttggccaaaatgaagcaggagccggtaaaa 420

[RhoA]_Dnmt3a(+/-)_PTCL_2_(AF%(XI)x2) cttcggaatgacgagcacacgagacgggagttggccaaaatgaagcaggagccggtaaaa 420

[RhoA]_Dnmt3a(+/-)_PTCL_3_(AF(IX)i1) cttcggaatgacgagcacacgagacgggagttggccaaaatgaagcaggagccggtaaaa 420

[RhoA]_Dnmt3a(-/-)_PTCL_1_(AF(IX)d4) cttcggaatgacgagcacacgagacgggagttggccaaaatgaagcaggagccggtaaaa 420

[RhoA]_Dnmt3a(-/-)_PTCL_2_(AF(IX)i4) cttcggaatgacgagcacacgagacgggagttggccaaaatgaagcaggagccggtaaaa 420

[RhoA]_Dnmt3a(-/-)_PTCL_3_(AF(IX)q3) cttcggaatgacgagcacacgagacgggagttggccaaaatgaagcaggagccggtaaaa 420

************************************************************

[RhoA]_Dnmt3a(+/+)_WT_CD8_Reference cctgaagaaggcagagatatggcaaacaggattggcgcttttgggtacatggagtgttca 480

[RhoA]_Dnmt3a(+/-)_PTCL_1_(AF(X)s8) cctgaagaaggcagagatatggcaaacaggattggcgcttttgggtacatggagtgttca 480

[RhoA]_Dnmt3a(+/-)_PTCL_2_(AF%(XI)x2) cctgaagaaggcagagatatggcaaacaggattggcgcttttgggtacatggagtgttca 480

[RhoA]_Dnmt3a(+/-)_PTCL_3_(AF(IX)i1) cctgaagaaggcagagatatggcaaacaggattggcgcttttgggtacatggagtgttca 480

[RhoA]_Dnmt3a(-/-)_PTCL_1_(AF(IX)d4) cctgaagaaggcagagatatggcaaacaggattggcgcttttgggtacatggagtgttca 480

[RhoA]_Dnmt3a(-/-)_PTCL_2_(AF(IX)i4) cctgaagaaggcagagatatggcaaacaggattggcgcttttgggtacatggagtgttca 480

[RhoA]_Dnmt3a(-/-)_PTCL_3_(AF(IX)q3) cctgaagaaggcagagatatggcaaacaggattggcgcttttgggtacatggagtgttca 480

************************************************************

[RhoA]_Dnmt3a(+/+)_WT_CD8_Reference gcaaagaccaaagatggagtgagagaggtttttgagatggccacgagagctgctctgcaa 540

[RhoA]_Dnmt3a(+/-)_PTCL_1_(AF(X)s8) gcaaagaccaaagatggagtgagagaggtttttgagatggccacgagagctgctctgcaa 540

[RhoA]_Dnmt3a(+/-)_PTCL_2_(AF%(XI)x2) gcaaagaccaaagatggagtgagagaggtttttgagatggccacgagagctgctctgcaa 540

[RhoA]_Dnmt3a(+/-)_PTCL_3_(AF(IX)i1) gcaaagaccaaagatggagtgagagaggtttttgagatggccacgagagctgctctgcaa 540

[RhoA]_Dnmt3a(-/-)_PTCL_1_(AF(IX)d4) gcaaagaccaaagatggagtgagagaggtttttgagatggccacgagagctgctctgcaa 540

[RhoA]_Dnmt3a(-/-)_PTCL_2_(AF(IX)i4) gcaaagaccaaagatggagtgagagaggtttttgagatggccacgagagctgctctgcaa 540

[RhoA]_Dnmt3a(-/-)_PTCL_3_(AF(IX)q3) gcaaagaccaaagatggagtgagagaggtttttgagatggccacgagagctgctctgcaa 540

************************************************************

[RhoA]_Dnmt3a(+/+)_WT_CD8_Reference gctagacgtgggaagaaaaagtctgggtgcctcatcttgtga 582

[RhoA]_Dnmt3a(+/-)_PTCL_1_(AF(X)s8) gctagacgtgggaagaaaaagtctgggtgcctcatcttgtga 582

[RhoA]_Dnmt3a(+/-)_PTCL_2_(AF%(XI)x2) gctagacgtgggaagaaaaagtctgggtgcctcatcttgtga 582

[RhoA]_Dnmt3a(+/-)_PTCL_3_(AF(IX)i1) gctagacgtgggaagaaaaagtctgggtgcctcatcttgtga 582

[RhoA]_Dnmt3a(-/-)_PTCL_1_(AF(IX)d4) gctagacgtgggaagaaaaagtctgggtgcctcatcttgtga 582

[RhoA]_Dnmt3a(-/-)_PTCL_2_(AF(IX)i4) gctagacgtgggaagaaaaagtctgggtgcctcatcttgtga 582

[RhoA]_Dnmt3a(-/-)_PTCL_3_(AF(IX)q3) gctagacgtgggaagaaaaagtctgggtgcctcatcttgtga 582

******************************************

**Sequencing of Tet2 transcript cDNA coding domain sequence in mouse Dnmt3a(+/-) and Dnmt3a(Δ/Δ) PTCL shows no mutations**

[Tet2]_Dnmt3a(+/+)_WT_CD8_Reference atggaacaggacagaaccacccatgctgagggcaccagactgagtccattcctgatagca 60

[Tet2]_Dnmt3a(+/-)_PTCL_1_(AF(X)s8) atggaacaggacagaaccacccatgctgagggcaccagactgagtccattcctgatagca 60

[Tet2]_Dnmt3a(+/-)_PTCL_2_(AF%(XI)x2) atggaacaggacagaaccacccatgctgagggcaccagactgagtccattcctgatagca 60

[Tet2]_Dnmt3a(-/-)_PTCL_1_(AF(IX)d4) atggaacaggacagaaccacccatgctgagggcaccagactgagtccattcctgatagca 60

[Tet2]_Dnmt3a(-/-)_PTCL_2_(AF(IX)i4) atggaacaggacagaaccacccatgctgagggcaccagactgagtccattcctgatagca 60

************************************************************

[Tet2]_Dnmt3a(+/+)_WT_CD8_Reference ccaccttctcccatcagccatacagaacctctggctgtcaaactccagaatggaagcccg 120

[Tet2]_Dnmt3a(+/-)_PTCL_1_(AF(X)s8) ccaccttctcccatcagccatacagaacctctggctgtcaaactccagaatggaagcccg 120

[Tet2]_Dnmt3a(+/-)_PTCL_2_(AF%(XI)x2) ccaccttctcccatcagccatacagaacctctggctgtcaaactccagaatggaagcccg 120

[Tet2]_Dnmt3a(-/-)_PTCL_1_(AF(IX)d4) ccaccttctcccatcagccatacagaacctctggctgtcaaactccagaatggaagcccg 120

[Tet2]_Dnmt3a(-/-)_PTCL_2_(AF(IX)i4) ccaccttctcccatcagccatacagaacctctggctgtcaaactccagaatggaagcccg 120

************************************************************

[Tet2]_Dnmt3a(+/+)_WT_CD8_Reference ttagcagagagacctcatccagaagtaaatggagacaccaagtggcaatcttcccaaagc 180

[Tet2]_Dnmt3a(+/-)_PTCL_1_(AF(X)s8) ttagcagagagacctcatccagaagtaaatggagacaccaagtggcaatcttcccaaagc 180

[Tet2]_Dnmt3a(+/-)_PTCL_2_(AF%(XI)x2) ttagcagagagacctcatccagaagtaaatggagacaccaagtggcaatcttcccaaagc 180

[Tet2]_Dnmt3a(-/-)_PTCL_1_(AF(IX)d4) ttagcagagagacctcatccagaagtaaatggagacaccaagtggcaatcttcccaaagc 180

[Tet2]_Dnmt3a(-/-)_PTCL_2_(AF(IX)i4) ttagcagagagacctcatccagaagtaaatggagacaccaagtggcaatcttcccaaagc 180

************************************************************

[Tet2]_Dnmt3a(+/+)_WT_CD8_Reference tgttatggaatatcccacatgaaaggcagccagagcagtcatgagagtccacatgaagac 240

[Tet2]_Dnmt3a(+/-)_PTCL_1_(AF(X)s8) tgttatggaatatcccacatgaaaggcagccagagcagtcatgagagtccacatgaagac 240

[Tet2]_Dnmt3a(+/-)_PTCL_2_(AF%(XI)x2) tgttatggaatatcccacatgaaaggcagccagagcagtcatgagagtccacatgaagac 240

[Tet2]_Dnmt3a(-/-)_PTCL_1_(AF(IX)d4) tgttatggaatatcccacatgaaaggcagccagagcagtcatgagagtccacatgaagac 240

[Tet2]_Dnmt3a(-/-)_PTCL_2_(AF(IX)i4) tgttatggaatatcccacatgaaaggcagccagagcagtcatgagagtccacatgaagac 240

************************************************************

[Tet2]_Dnmt3a(+/+)_WT_CD8_Reference agagggtattccaggtgcttacagaatggagggataaaacgcacagtcagtgaaccatct 300

[Tet2]_Dnmt3a(+/-)_PTCL_1_(AF(X)s8) agagggtattccaggtgcttacagaatggagggataaaacgcacagtcagtgaaccatct 300

[Tet2]_Dnmt3a(+/-)_PTCL_2_(AF%(XI)x2) agagggtattccaggtgcttacagaatggagggataaaacgcacagtcagtgaaccatct 300

[Tet2]_Dnmt3a(-/-)_PTCL_1_(AF(IX)d4) agagggtattccaggtgcttacagaatggagggataaaacgcacagtcagtgaaccatct 300

[Tet2]_Dnmt3a(-/-)_PTCL_2_(AF(IX)i4) agagggtattccaggtgcttacagaatggagggataaaacgcacagtcagtgaaccatct 300

************************************************************

[Tet2]_Dnmt3a(+/+)_WT_CD8_Reference ctctctgggctccatccaaacaagatattgaaactagaccaaaaagctaagggagaaagc 360

[Tet2]_Dnmt3a(+/-)_PTCL_1_(AF(X)s8) ctctctgggctccatccaaacaagatattgaaactagaccaaaaagctaagggagaaagc 360

[Tet2]_Dnmt3a(+/-)_PTCL_2_(AF%(XI)x2) ctctctgggctccatccaaacaagatattgaaactagaccaaaaagctaagggagaaagc 360

[Tet2]_Dnmt3a(-/-)_PTCL_1_(AF(IX)d4) ctctctgggctccatccaaacaagatattgaaactagaccaaaaagctaagggagaaagc 360

[Tet2]_Dnmt3a(-/-)_PTCL_2_(AF(IX)i4) ctctctgggctccatccaaacaagatattgaaactagaccaaaaagctaagggagaaagc 360

************************************************************

[Tet2]_Dnmt3a(+/+)_WT_CD8_Reference aatatcttcgaggaaagccaagaaagaaaccacggtaaaagcagccgtcagccaaatgtc 420

[Tet2]_Dnmt3a(+/-)_PTCL_1_(AF(X)s8) aatatcttcgaggaaagccaagaaagaaaccacggtaaaagcagccgtcagccaaatgtc 420

[Tet2]_Dnmt3a(+/-)_PTCL_2_(AF%(XI)x2) aatatcttcgaggaaagccaagaaagaaaccacggtaaaagcagccgtcagccaaatgtc 420

[Tet2]_Dnmt3a(-/-)_PTCL_1_(AF(IX)d4) aatatcttcgaggaaagccaagaaagaaaccacggtaaaagcagccgtcagccaaatgtc 420

[Tet2]_Dnmt3a(-/-)_PTCL_2_(AF(IX)i4) aatatcttcgaggaaagccaagaaagaaaccacggtaaaagcagccgtcagccaaatgtc 420

************************************************************

[Tet2]_Dnmt3a(+/+)_WT_CD8_Reference tccggactaagtgataatggagagcccgtgacctcgaccacccaggaaagttcaggtgca 480

[Tet2]_Dnmt3a(+/-)_PTCL_1_(AF(X)s8) tccggactaagtgataatggagagcccgtgacctcgaccacccaggaaagttcaggtgca 480

[Tet2]_Dnmt3a(+/-)_PTCL_2_(AF%(XI)x2) tccggactaagtgataatggagagcccgtgacctcgaccacccaggaaagttcaggtgca 480

[Tet2]_Dnmt3a(-/-)_PTCL_1_(AF(IX)d4) tccggactaagtgataatggagagcccgtgacctcgaccacccaggaaagttcaggtgca 480

[Tet2]_Dnmt3a(-/-)_PTCL_2_(AF(IX)i4) tccggactaagtgataatggagagcccgtgacctcgaccacccaggaaagttcaggtgca 480

************************************************************

[Tet2]_Dnmt3a(+/+)_WT_CD8_Reference gatgctttcccaacacggaactacaatggagttgaaattcaggttctcaacgagcaggaa 540

[Tet2]_Dnmt3a(+/-)_PTCL_1_(AF(X)s8) gatgctttcccaacacggaactacaatggagttgaaattcaggttctcaacgagcaggaa 540

[Tet2]_Dnmt3a(+/-)_PTCL_2_(AF%(XI)x2) gatgctttcccaacacggaactacaatggagttgaaattcaggttctcaacgagcaggaa 540

[Tet2]_Dnmt3a(-/-)_PTCL_1_(AF(IX)d4) gatgctttcccaacacggaactacaatggagttgaaattcaggttctcaacgagcaggaa 540

[Tet2]_Dnmt3a(-/-)_PTCL_2_(AF(IX)i4) gatgctttcccaacacggaactacaatggagttgaaattcaggttctcaacgagcaggaa 540

************************************************************

[Tet2]_Dnmt3a(+/+)_WT_CD8_Reference ggggaaaaaggcaggagcgttacattacttaaaaacaagattgtgctaatgcctaatggt 600

[Tet2]_Dnmt3a(+/-)_PTCL_1_(AF(X)s8) ggggaaaaaggcaggagcgttacattacttaaaaacaagattgtgctaatgcctaatggt 600

[Tet2]_Dnmt3a(+/-)_PTCL_2_(AF%(XI)x2) ggggaaaaaggcaggagcgttacattacttaaaaacaagattgtgctaatgcctaatggt 600

[Tet2]_Dnmt3a(-/-)_PTCL_1_(AF(IX)d4) ggggaaaaaggcaggagcgttacattacttaaaaacaagattgtgctaatgcctaatggt 600

[Tet2]_Dnmt3a(-/-)_PTCL_2_(AF(IX)i4) ggggaaaaaggcaggagcgttacattacttaaaaacaagattgtgctaatgcctaatggt 600

************************************************************

[Tet2]_Dnmt3a(+/+)_WT_CD8_Reference gctacagtttctgcccattctgaggagaacacacgtggtgaactcctggaaaaaacacag 660

[Tet2]_Dnmt3a(+/-)_PTCL_1_(AF(X)s8) gctacagtttctgcccattctgaggagaacacacgtggtgaactcctggaaaaaacacag 660

[Tet2]_Dnmt3a(+/-)_PTCL_2_(AF%(XI)x2) gctacagtttctgcccattctgaggagaacacacgtggtgaactcctggaaaaaacacag 660

[Tet2]_Dnmt3a(-/-)_PTCL_1_(AF(IX)d4) gctacagtttctgcccattctgaggagaacacacgtggtgaactcctggaaaaaacacag 660

[Tet2]_Dnmt3a(-/-)_PTCL_2_(AF(IX)i4) gctacagtttctgcccattctgaggagaacacacgtggtgaactcctggaaaaaacacag 660

************************************************************

[Tet2]_Dnmt3a(+/+)_WT_CD8_Reference tgttatccagattgtgtctccattgccgtgcagagtaccgcatctcatgtaaacacccct 720

[Tet2]_Dnmt3a(+/-)_PTCL_1_(AF(X)s8) tgttatccagattgtgtctccattgccgtgcagagtaccgcatctcatgtaaacacccct 720

[Tet2]_Dnmt3a(+/-)_PTCL_2_(AF%(XI)x2) tgttatccagattgtgtctccattgccgtgcagagtaccgcatctcatgtaaacacccct 720

[Tet2]_Dnmt3a(-/-)_PTCL_1_(AF(IX)d4) tgttatccagattgtgtctccattgccgtgcagagtaccgcatctcatgtaaacacccct 720

[Tet2]_Dnmt3a(-/-)_PTCL_2_(AF(IX)i4) tgttatccagattgtgtctccattgccgtgcagagtaccgcatctcatgtaaacacccct 720

************************************************************

[Tet2]_Dnmt3a(+/+)_WT_CD8_Reference agcagtcaggctgctatcgagttgtcccacgagatccctcaaccatcgcttacctcagcg 780

[Tet2]_Dnmt3a(+/-)_PTCL_1_(AF(X)s8) agcagtcaggctgctatcgagttgtcccacgagatccctcaaccatcgcttacctcagcg 780

[Tet2]_Dnmt3a(+/-)_PTCL_2_(AF%(XI)x2) agcagtcaggctgctatcgagttgtcccacgagatccctcaaccatcgcttacctcagcg 780

[Tet2]_Dnmt3a(-/-)_PTCL_1_(AF(IX)d4) agcagtcaggctgctatcgagttgtcccacgagatccctcaaccatcgcttacctcagcg 780

[Tet2]_Dnmt3a(-/-)_PTCL_2_(AF(IX)i4) agcagtcaggctgctatcgagttgtcccacgagatccctcaaccatcgcttacctcagcg 780

************************************************************

[Tet2]_Dnmt3a(+/+)_WT_CD8_Reference cagatcaatttctcccagacctcaagcttgcagctgcctccagagccagctgcaatggtg 840

[Tet2]_Dnmt3a(+/-)_PTCL_1_(AF(X)s8) cagatcaatttctcccagacctcaagcttgcagctgcctccagagccagctgcaatggtg 840

[Tet2]_Dnmt3a(+/-)_PTCL_2_(AF%(XI)x2) cagatcaatttctcccagacctcaagcttgcagctgcctccagagccagctgcaatggtg 840

[Tet2]_Dnmt3a(-/-)_PTCL_1_(AF(IX)d4) cagatcaatttctcccagacctcaagcttgcagctgcctccagagccagctgcaatggtg 840

[Tet2]_Dnmt3a(-/-)_PTCL_2_(AF(IX)i4) cagatcaatttctcccagacctcaagcttgcagctgcctccagagccagctgcaatggtg 840

************************************************************

[Tet2]_Dnmt3a(+/+)_WT_CD8_Reference actaaggcctgtgatgctgataatgccagtaaaccagctatagtaccaggtacctgtcct 900

[Tet2]_Dnmt3a(+/-)_PTCL_1_(AF(X)s8) actaaggcctgtgatgctgataatgccagtaaaccagctatagtaccaggtacctgtcct 900

[Tet2]_Dnmt3a(+/-)_PTCL_2_(AF%(XI)x2) actaaggcctgtgatgctgataatgccagtaaaccagctatagtaccaggtacctgtcct 900

[Tet2]_Dnmt3a(-/-)_PTCL_1_(AF(IX)d4) actaaggcctgtgatgctgataatgccagtaaaccagctatagtaccaggtacctgtcct 900

[Tet2]_Dnmt3a(-/-)_PTCL_2_(AF(IX)i4) actaaggcctgtgatgctgataatgccagtaaaccagctatagtaccaggtacctgtcct 900

************************************************************

[Tet2]_Dnmt3a(+/+)_WT_CD8_Reference tttcagaaagcagaacaccaacaaaagtcagctttggacataggcccatctcgtgcagaa 960

[Tet2]_Dnmt3a(+/-)_PTCL_1_(AF(X)s8) tttcagaaagcagaacaccaacaaaagtcagctttggacataggcccatctcgtgcagaa 960

[Tet2]_Dnmt3a(+/-)_PTCL_2_(AF%(XI)x2) tttcagaaagcagaacaccaacaaaagtcagctttggacataggcccatctcgtgcagaa 960

[Tet2]_Dnmt3a(-/-)_PTCL_1_(AF(IX)d4) tttcagaaagcagaacaccaacaaaagtcagctttggacataggcccatctcgtgcagaa 960

[Tet2]_Dnmt3a(-/-)_PTCL_2_(AF(IX)i4) tttcagaaagcagaacaccaacaaaagtcagctttggacataggcccatctcgtgcagaa 960

************************************************************

[Tet2]_Dnmt3a(+/+)_WT_CD8_Reference aacaaaaccatccaaggaagcatggagctatttgctgaagaatactatcctagttccgac 1020

[Tet2]_Dnmt3a(+/-)_PTCL_1_(AF(X)s8) aacaaaaccatccaaggaagcatggagctatttgctgaagaatactatcctagttccgac 1020

[Tet2]_Dnmt3a(+/-)_PTCL_2_(AF%(XI)x2) aacaaaaccatccaaggaagcatggagctatttgctgaagaatactatcctagttccgac 1020

[Tet2]_Dnmt3a(-/-)_PTCL_1_(AF(IX)d4) aacaaaaccatccaaggaagcatggagctatttgctgaagaatactatcctagttccgac 1020

[Tet2]_Dnmt3a(-/-)_PTCL_2_(AF(IX)i4) aacaaaaccatccaaggaagcatggagctatttgctgaagaatactatcctagttccgac 1020

************************************************************

[Tet2]_Dnmt3a(+/+)_WT_CD8_Reference cggaatttgcaagcttcgcatggcagctctgaacagtattcaaagcaaaaggaaaccaat 1080

[Tet2]_Dnmt3a(+/-)_PTCL_1_(AF(X)s8) cggaatttgcaagcttcgcatggcagctctgaacagtattcaaagcaaaaggaaaccaat 1080

[Tet2]_Dnmt3a(+/-)_PTCL_2_(AF%(XI)x2) cggaatttgcaagcttcgcatggcagctctgaacagtattcaaagcaaaaggaaaccaat 1080

[Tet2]_Dnmt3a(-/-)_PTCL_1_(AF(IX)d4) cggaatttgcaagcttcgcatggcagctctgaacagtattcaaagcaaaaggaaaccaat 1080

[Tet2]_Dnmt3a(-/-)_PTCL_2_(AF(IX)i4) cggaatttgcaagcttcgcatggcagctctgaacagtattcaaagcaaaaggaaaccaat 1080

************************************************************

[Tet2]_Dnmt3a(+/+)_WT_CD8_Reference ggtgcttacttcaggcaaagctcgaagttccctaaagattccatctctcccactactgtg 1140

[Tet2]_Dnmt3a(+/-)_PTCL_1_(AF(X)s8) ggtgcttacttcaggcaaagctcgaagttccctaaagattccatctctcccactactgtg 1140

[Tet2]_Dnmt3a(+/-)_PTCL_2_(AF%(XI)x2) ggtgcttacttcaggcaaagctcgaagttccctaaagattccatctctcccactactgtg 1140

[Tet2]_Dnmt3a(-/-)_PTCL_1_(AF(IX)d4) ggtgcttacttcaggcaaagctcgaagttccctaaagattccatctctcccactactgtg 1140

[Tet2]_Dnmt3a(-/-)_PTCL_2_(AF(IX)i4) ggtgcttacttcaggcaaagctcgaagttccctaaagattccatctctcccactactgtg 1140

************************************************************

[Tet2]_Dnmt3a(+/+)_WT_CD8_Reference accccaccgtcacaatcacttcttgctccccgtcttgttcttcagcctcctttagaagga 1200

[Tet2]_Dnmt3a(+/-)_PTCL_1_(AF(X)s8) accccaccgtcacaatcacttcttgctccccgtcttgttcttcagcctcctttagaagga 1200

[Tet2]_Dnmt3a(+/-)_PTCL_2_(AF%(XI)x2) accccaccgtcacaatcacttcttgctccccgtcttgttcttcagcctcctttagaagga 1200

[Tet2]_Dnmt3a(-/-)_PTCL_1_(AF(IX)d4) accccaccgtcacaatcacttcttgctccccgtcttgttcttcagcctcctttagaagga 1200

[Tet2]_Dnmt3a(-/-)_PTCL_2_(AF(IX)i4) accccaccgtcacaatcacttcttgctccccgtcttgttcttcagcctcctttagaagga 1200

************************************************************

[Tet2]_Dnmt3a(+/+)_WT_CD8_Reference aaaggcgctctaaatgatgtagctttggaagaacaccatgactaccccaaccgaagcaac 1260

[Tet2]_Dnmt3a(+/-)_PTCL_1_(AF(X)s8) aaaggcgctctaaatgatgtagctttggaagaacaccatgactaccccaaccgaagcaac 1260

[Tet2]_Dnmt3a(+/-)_PTCL_2_(AF%(XI)x2) aaaggcgctctaaatgatgtagctttggaagaacaccatgactaccccaaccgaagcaac 1260

[Tet2]_Dnmt3a(-/-)_PTCL_1_(AF(IX)d4) aaaggcgctctaaatgatgtagctttggaagaacaccatgactaccccaaccgaagcaac 1260

[Tet2]_Dnmt3a(-/-)_PTCL_2_(AF(IX)i4) aaaggcgctctaaatgatgtagctttggaagaacaccatgactaccccaaccgaagcaac 1260

************************************************************

[Tet2]_Dnmt3a(+/+)_WT_CD8_Reference cgaactcttttaagggaagggaaaatagaccatcaacccaagacatcatctagccagagt 1320

[Tet2]_Dnmt3a(+/-)_PTCL_1_(AF(X)s8) cgaactcttttaagggaagggaaaatagaccatcaacccaagacatcatctagccagagt 1320

[Tet2]_Dnmt3a(+/-)_PTCL_2_(AF%(XI)x2) cgaactcttttaagggaagggaaaatagaccatcaacccaagacatcatctagccagagt 1320

[Tet2]_Dnmt3a(-/-)_PTCL_1_(AF(IX)d4) cgaactcttttaagggaagggaaaatagaccatcaacccaagacatcatctagccagagt 1320

[Tet2]_Dnmt3a(-/-)_PTCL_2_(AF(IX)i4) cgaactcttttaagggaagggaaaatagaccatcaacccaagacatcatctagccagagt 1320

************************************************************

[Tet2]_Dnmt3a(+/+)_WT_CD8_Reference ctgaatccatctgtacatacacccaacccccccttgatgcttccagaacagcatcagaat 1380

[Tet2]_Dnmt3a(+/-)_PTCL_1_(AF(X)s8) ctgaatccatctgtacatacacccaacccccccttgatgcttccagaacagcatcagaat 1380

[Tet2]_Dnmt3a(+/-)_PTCL_2_(AF%(XI)x2) ctgaatccatctgtacatacacccaacccccccttgatgcttccagaacagcatcagaat 1380

[Tet2]_Dnmt3a(-/-)_PTCL_1_(AF(IX)d4) ctgaatccatctgtacatacacccaacccccccttgatgcttccagaacagcatcagaat 1380

[Tet2]_Dnmt3a(-/-)_PTCL_2_(AF(IX)i4) ctgaatccatctgtacatacacccaacccccccttgatgcttccagaacagcatcagaat 1380

************************************************************

[Tet2]_Dnmt3a(+/+)_WT_CD8_Reference gattgtggctcaccgagccctgaaaagtcaagaaaaatgtcagaatatctcatgtattac 1440

[Tet2]_Dnmt3a(+/-)_PTCL_1_(AF(X)s8) gattgtggctcaccgagccctgaaaagtcaagaaaaatgtcagaatatctcatgtattac 1440

[Tet2]_Dnmt3a(+/-)_PTCL_2_(AF%(XI)x2) gattgtggctcaccgagccctgaaaagtcaagaaaaatgtcagaatatctcatgtattac 1440

[Tet2]_Dnmt3a(-/-)_PTCL_1_(AF(IX)d4) gattgtggctcaccgagccctgaaaagtcaagaaaaatgtcagaatatctcatgtattac 1440

[Tet2]_Dnmt3a(-/-)_PTCL_2_(AF(IX)i4) gattgtggctcaccgagccctgaaaagtcaagaaaaatgtcagaatatctcatgtattac 1440

************************************************************

[Tet2]_Dnmt3a(+/+)_WT_CD8_Reference ctgccaaatcatggccacagtggaggtttacaagaacatagccaatacctgatggggcac 1500

[Tet2]_Dnmt3a(+/-)_PTCL_1_(AF(X)s8) ctgccaaatcatggccacagtggaggtttacaagaacatagccaatacctgatggggcac 1500

[Tet2]_Dnmt3a(+/-)_PTCL_2_(AF%(XI)x2) ctgccaaatcatggccacagtggaggtttacaagaacatagccaatacctgatggggcac 1500

[Tet2]_Dnmt3a(-/-)_PTCL_1_(AF(IX)d4) ctgccaaatcatggccacagtggaggtttacaagaacatagccaatacctgatggggcac 1500

[Tet2]_Dnmt3a(-/-)_PTCL_2_(AF(IX)i4) ctgccaaatcatggccacagtggaggtttacaagaacatagccaatacctgatggggcac 1500

************************************************************

[Tet2]_Dnmt3a(+/+)_WT_CD8_Reference agggagcaagagattccgaaggatgcaaacgggaaacaaacgcaaggctctgtacaggca 1560

[Tet2]_Dnmt3a(+/-)_PTCL_1_(AF(X)s8) agggagcaagagattccgaaggatgcaaacgggaaacaaacgcaaggctctgtacaggca 1560

[Tet2]_Dnmt3a(+/-)_PTCL_2_(AF%(XI)x2) agggagcaagagattccgaaggatgcaaacgggaaacaaacgcaaggctctgtacaggca 1560

[Tet2]_Dnmt3a(-/-)_PTCL_1_(AF(IX)d4) agggagcaagagattccgaaggatgcaaacgggaaacaaacgcaaggctctgtacaggca 1560

[Tet2]_Dnmt3a(-/-)_PTCL_2_(AF(IX)i4) agggagcaagagattccgaaggatgcaaacgggaaacaaacgcaaggctctgtacaggca 1560

************************************************************

[Tet2]_Dnmt3a(+/+)_WT_CD8_Reference gcacctggctggatagaactgaaagccccgaatttgcatgaagcactccatcagacaaaa 1620

[Tet2]_Dnmt3a(+/-)_PTCL_1_(AF(X)s8) gcacctggctggatagaactgaaagccccgaatttgcatgaagcactccatcagacaaaa 1620

[Tet2]_Dnmt3a(+/-)_PTCL_2_(AF%(XI)x2) gcacctggctggatagaactgaaagccccgaatttgcatgaagcactccatcagacaaaa 1620

[Tet2]_Dnmt3a(-/-)_PTCL_1_(AF(IX)d4) gcacctggctggatagaactgaaagccccgaatttgcatgaagcactccatcagacaaaa 1620

[Tet2]_Dnmt3a(-/-)_PTCL_2_(AF(IX)i4) gcacctggctggatagaactgaaagccccgaatttgcatgaagcactccatcagacaaaa 1620

************************************************************

[Tet2]_Dnmt3a(+/+)_WT_CD8_Reference cgcaaggatatatccttgcactcagtcctccactctcagaccggccctgtcaatcagatg 1680

[Tet2]_Dnmt3a(+/-)_PTCL_1_(AF(X)s8) cgcaaggatatatccttgcactcagtcctccactctcagaccggccctgtcaatcagatg 1680

[Tet2]_Dnmt3a(+/-)_PTCL_2_(AF%(XI)x2) cgcaaggatatatccttgcactcagtcctccactctcagaccggccctgtcaatcagatg 1680

[Tet2]_Dnmt3a(-/-)_PTCL_1_(AF(IX)d4) cgcaaggatatatccttgcactcagtcctccactctcagaccggccctgtcaatcagatg 1680

[Tet2]_Dnmt3a(-/-)_PTCL_2_(AF(IX)i4) cgcaaggatatatccttgcactcagtcctccactctcagaccggccctgtcaatcagatg 1680

************************************************************

[Tet2]_Dnmt3a(+/+)_WT_CD8_Reference agctccaaacagtccactggcaatgtcaacatgccaggaggattccaaaggctaccttac 1740

[Tet2]_Dnmt3a(+/-)_PTCL_1_(AF(X)s8) agctccaaacagtccactggcaatgtcaacatgccaggaggattccaaaggctaccttac 1740

[Tet2]_Dnmt3a(+/-)_PTCL_2_(AF%(XI)x2) agctccaaacagtccactggcaatgtcaacatgccaggaggattccaaaggctaccttac 1740

[Tet2]_Dnmt3a(-/-)_PTCL_1_(AF(IX)d4) agctccaaacagtccactggcaatgtcaacatgccaggaggattccaaaggctaccttac 1740

[Tet2]_Dnmt3a(-/-)_PTCL_2_(AF(IX)i4) agctccaaacagtccactggcaatgtcaacatgccaggaggattccaaaggctaccttac 1740

************************************************************

[Tet2]_Dnmt3a(+/+)_WT_CD8_Reference ctccagaaaacagcccagccagagcagaaggcacaaatgtaccaagtgcaagtgaaccaa 1800

[Tet2]_Dnmt3a(+/-)_PTCL_1_(AF(X)s8) ctccagaaaacagcccagccagagcagaaggcacaaatgtaccaagtgcaagtgaaccaa 1800

[Tet2]_Dnmt3a(+/-)_PTCL_2_(AF%(XI)x2) ctccagaaaacagcccagccagagcagaaggcacaaatgtaccaagtgcaagtgaaccaa 1800

[Tet2]_Dnmt3a(-/-)_PTCL_1_(AF(IX)d4) ctccagaaaacagcccagccagagcagaaggcacaaatgtaccaagtgcaagtgaaccaa 1800

[Tet2]_Dnmt3a(-/-)_PTCL_2_(AF(IX)i4) ctccagaaaacagcccagccagagcagaaggcacaaatgtaccaagtgcaagtgaaccaa 1800

************************************************************

[Tet2]_Dnmt3a(+/+)_WT_CD8_Reference ggaccgtctccaggtatgggggaccaacatcttcagttccagaaagctttataccaggag 1860

[Tet2]_Dnmt3a(+/-)_PTCL_1_(AF(X)s8) ggaccgtctccaggtatgggggaccaacatcttcagttccagaaagctttataccaggag 1860

[Tet2]_Dnmt3a(+/-)_PTCL_2_(AF%(XI)x2) ggaccgtctccaggtatgggggaccaacatcttcagttccagaaagctttataccaggag 1860

[Tet2]_Dnmt3a(-/-)_PTCL_1_(AF(IX)d4) ggaccgtctccaggtatgggggaccaacatcttcagttccagaaagctttataccaggag 1860

[Tet2]_Dnmt3a(-/-)_PTCL_2_(AF(IX)i4) ggaccgtctccaggtatgggggaccaacatcttcagttccagaaagctttataccaggag 1860

************************************************************

[Tet2]_Dnmt3a(+/+)_WT_CD8_Reference tgcatccccaggacagatccgtcatctgaggctcacccgcaagcaccgagcgttcctcag 1920

[Tet2]_Dnmt3a(+/-)_PTCL_1_(AF(X)s8) tgcatccccaggacagatccgtcatctgaggctcacccgcaagcaccgagcgttcctcag 1920

[Tet2]_Dnmt3a(+/-)_PTCL_2_(AF%(XI)x2) tgcatccccaggacagatccgtcatctgaggctcacccgcaagcaccgagcgttcctcag 1920

[Tet2]_Dnmt3a(-/-)_PTCL_1_(AF(IX)d4) tgcatccccaggacagatccgtcatctgaggctcacccgcaagcaccgagcgttcctcag 1920

[Tet2]_Dnmt3a(-/-)_PTCL_2_(AF(IX)i4) tgcatccccaggacagatccgtcatctgaggctcacccgcaagcaccgagcgttcctcag 1920

************************************************************

[Tet2]_Dnmt3a(+/+)_WT_CD8_Reference tatcatttccagcaaagagtaaatccctccagtgataagcatttgagtcaacaggccaca 1980

[Tet2]_Dnmt3a(+/-)_PTCL_1_(AF(X)s8) tatcatttccagcaaagagtaaatccctccagtgataagcatttgagtcaacaggccaca 1980

[Tet2]_Dnmt3a(+/-)_PTCL_2_(AF%(XI)x2) tatcatttccagcaaagagtaaatccctccagtgataagcatttgagtcaacaggccaca 1980

[Tet2]_Dnmt3a(-/-)_PTCL_1_(AF(IX)d4) tatcatttccagcaaagagtaaatccctccagtgataagcatttgagtcaacaggccaca 1980

[Tet2]_Dnmt3a(-/-)_PTCL_2_(AF(IX)i4) tatcatttccagcaaagagtaaatccctccagtgataagcatttgagtcaacaggccaca 1980

************************************************************

[Tet2]_Dnmt3a(+/+)_WT_CD8_Reference gagactcaacggttatcaggctttttacaacatactcctcagacgcaggcatcacaaaca 2040

[Tet2]_Dnmt3a(+/-)_PTCL_1_(AF(X)s8) gagactcaacggttatcaggctttttacaacatactcctcagacgcaggcatcacaaaca 2040

[Tet2]_Dnmt3a(+/-)_PTCL_2_(AF%(XI)x2) gagactcaacggttatcaggctttttacaacatactcctcagacgcaggcatcacaaaca 2040

[Tet2]_Dnmt3a(-/-)_PTCL_1_(AF(IX)d4) gagactcaacggttatcaggctttttacaacatactcctcagacgcaggcatcacaaaca 2040

[Tet2]_Dnmt3a(-/-)_PTCL_2_(AF(IX)i4) gagactcaacggttatcaggctttttacaacatactcctcagacgcaggcatcacaaaca 2040

************************************************************

[Tet2]_Dnmt3a(+/+)_WT_CD8_Reference ccagcatcccagaactcaaatttccctcaaatctgccagcagcagcagcagcagcagtta 2100

[Tet2]_Dnmt3a(+/-)_PTCL_1_(AF(X)s8) ccagcatcccagaactcaaatttccctcaaatctgccagcagcagcagcagcagcagtta 2100

[Tet2]_Dnmt3a(+/-)_PTCL_2_(AF%(XI)x2) ccagcatcccagaactcaaatttccctcaaatctgccagcagcagcagcagcagcagtta 2100

[Tet2]_Dnmt3a(-/-)_PTCL_1_(AF(IX)d4) ccagcatcccagaactcaaatttccctcaaatctgccagcagcagcagcagcagcagtta 2100

[Tet2]_Dnmt3a(-/-)_PTCL_2_(AF(IX)i4) ccagcatcccagaactcaaatttccctcaaatctgccagcagcagcagcagcagcagtta 2100

************************************************************

[Tet2]_Dnmt3a(+/+)_WT_CD8_Reference cagaggaagaataaagagcaaatgcctcagactttctctcatctccaaggtagcaatgat 2160

[Tet2]_Dnmt3a(+/-)_PTCL_1_(AF(X)s8) cagaggaagaataaagagcaaatgcctcagactttctctcatctccaaggtagcaatgat 2160

[Tet2]_Dnmt3a(+/-)_PTCL_2_(AF%(XI)x2) cagaggaagaataaagagcaaatgcctcagactttctctcatctccaaggtagcaatgat 2160

[Tet2]_Dnmt3a(-/-)_PTCL_1_(AF(IX)d4) cagaggaagaataaagagcaaatgcctcagactttctctcatctccaaggtagcaatgat 2160

[Tet2]_Dnmt3a(-/-)_PTCL_2_(AF(IX)i4) cagaggaagaataaagagcaaatgcctcagactttctctcatctccaaggtagcaatgat 2160

************************************************************

[Tet2]_Dnmt3a(+/+)_WT_CD8_Reference aagcaaagagaaggctcgtgctttggccagattaaagtggaagaaagcttttgtgtcgga 2220

[Tet2]_Dnmt3a(+/-)_PTCL_1_(AF(X)s8) aagcaaagagaaggctcgtgctttggccagattaaagtggaagaaagcttttgtgtcgga 2220

[Tet2]_Dnmt3a(+/-)_PTCL_2_(AF%(XI)x2) aagcaaagagaaggctcgtgctttggccagattaaagtggaagaaagcttttgtgtcgga 2220

[Tet2]_Dnmt3a(-/-)_PTCL_1_(AF(IX)d4) aagcaaagagaaggctcgtgctttggccagattaaagtggaagaaagcttttgtgtcgga 2220

[Tet2]_Dnmt3a(-/-)_PTCL_2_(AF(IX)i4) aagcaaagagaaggctcgtgctttggccagattaaagtggaagaaagcttttgtgtcgga 2220

************************************************************

[Tet2]_Dnmt3a(+/+)_WT_CD8_Reference aatcagtactccaaatcaagtaatttccaaactcacaataatacccaaggggggttggag 2280

[Tet2]_Dnmt3a(+/-)_PTCL_1_(AF(X)s8) aatcagtactccaaatcaagtaatttccaaactcacaataatacccaaggggggttggag 2280

[Tet2]_Dnmt3a(+/-)_PTCL_2_(AF%(XI)x2) aatcagtactccaaatcaagtaatttccaaactcacaataatacccaaggggggttggag 2280

[Tet2]_Dnmt3a(-/-)_PTCL_1_(AF(IX)d4) aatcagtactccaaatcaagtaatttccaaactcacaataatacccaaggggggttggag 2280

[Tet2]_Dnmt3a(-/-)_PTCL_2_(AF(IX)i4) aatcagtactccaaatcaagtaatttccaaactcacaataatacccaaggggggttggag 2280

************************************************************

[Tet2]_Dnmt3a(+/+)_WT_CD8_Reference caagtacaaaatataaataaaaattttccttattcgaagatcttaacaccaaattcgagc 2340

[Tet2]_Dnmt3a(+/-)_PTCL_1_(AF(X)s8) caagtacaaaatataaataaaaattttccttattcgaagatcttaacaccaaattcgagc 2340

[Tet2]_Dnmt3a(+/-)_PTCL_2_(AF%(XI)x2) caagtacaaaatataaataaaaattttccttattcgaagatcttaacaccaaattcgagc 2340

[Tet2]_Dnmt3a(-/-)_PTCL_1_(AF(IX)d4) caagtacaaaatataaataaaaattttccttattcgaagatcttaacaccaaattcgagc 2340

[Tet2]_Dnmt3a(-/-)_PTCL_2_(AF(IX)i4) caagtacaaaatataaataaaaattttccttattcgaagatcttaacaccaaattcgagc 2340

************************************************************

[Tet2]_Dnmt3a(+/+)_WT_CD8_Reference aacttacagattctcccttcaaatgacacacacccggcttgtgagcgggaacaagctcta 2400

[Tet2]_Dnmt3a(+/-)_PTCL_1_(AF(X)s8) aacttacagattctcccttcaaatgacacacacccggcttgtgagcgggaacaagctcta 2400

[Tet2]_Dnmt3a(+/-)_PTCL_2_(AF%(XI)x2) aacttacagattctcccttcaaatgacacacacccggcttgtgagcgggaacaagctcta 2400

[Tet2]_Dnmt3a(-/-)_PTCL_1_(AF(IX)d4) aacttacagattctcccttcaaatgacacacacccggcttgtgagcgggaacaagctcta 2400

[Tet2]_Dnmt3a(-/-)_PTCL_2_(AF(IX)i4) aacttacagattctcccttcaaatgacacacacccggcttgtgagcgggaacaagctcta 2400

************************************************************

[Tet2]_Dnmt3a(+/+)_WT_CD8_Reference catcccgtaggaagtaagacctcaaacctgcagaacatgcagtatttcccgaataatgtg 2460

[Tet2]_Dnmt3a(+/-)_PTCL_1_(AF(X)s8) catcccgtaggaagtaagacctcaaacctgcagaacatgcagtatttcccgaataatgtg 2460

[Tet2]_Dnmt3a(+/-)_PTCL_2_(AF%(XI)x2) catcccgtaggaagtaagacctcaaacctgcagaacatgcagtatttcccgaataatgtg 2460

[Tet2]_Dnmt3a(-/-)_PTCL_1_(AF(IX)d4) catcccgtaggaagtaagacctcaaacctgcagaacatgcagtatttcccgaataatgtg 2460

[Tet2]_Dnmt3a(-/-)_PTCL_2_(AF(IX)i4) catcccgtaggaagtaagacctcaaacctgcagaacatgcagtatttcccgaataatgtg 2460

************************************************************

[Tet2]_Dnmt3a(+/+)_WT_CD8_Reference accccaaatcaggacgttcaccggtgctttcaggaacaagcgcagaagcctcagcaagct 2520

[Tet2]_Dnmt3a(+/-)_PTCL_1_(AF(X)s8) accccaaatcaggacgttcaccggtgctttcaggaacaagcgcagaagcctcagcaagct 2520

[Tet2]_Dnmt3a(+/-)_PTCL_2_(AF%(XI)x2) accccaaatcaggacgttcaccggtgctttcaggaacaagcgcagaagcctcagcaagct 2520

[Tet2]_Dnmt3a(-/-)_PTCL_1_(AF(IX)d4) accccaaatcaggacgttcaccggtgctttcaggaacaagcgcagaagcctcagcaagct 2520

[Tet2]_Dnmt3a(-/-)_PTCL_2_(AF(IX)i4) accccaaatcaggacgttcaccggtgctttcaggaacaagcgcagaagcctcagcaagct 2520

************************************************************

[Tet2]_Dnmt3a(+/+)_WT_CD8_Reference tcgtctctacaggggcttaaggacagaagccagggtgagtctccagccccaccagctgag 2580

[Tet2]_Dnmt3a(+/-)_PTCL_1_(AF(X)s8) tcgtctctacaggggcttaaggacagaagccagggtgagtctccagccccaccagctgag 2580

[Tet2]_Dnmt3a(+/-)_PTCL_2_(AF%(XI)x2) tcgtctctacaggggcttaaggacagaagccagggtgagtctccagccccaccagctgag 2580

[Tet2]_Dnmt3a(-/-)_PTCL_1_(AF(IX)d4) tcgtctctacaggggcttaaggacagaagccagggtgagtctccagccccaccagctgag 2580

[Tet2]_Dnmt3a(-/-)_PTCL_2_(AF(IX)i4) tcgtctctacaggggcttaaggacagaagccagggtgagtctccagccccaccagctgag 2580

************************************************************

[Tet2]_Dnmt3a(+/+)_WT_CD8_Reference gcagctcaacagaggtatttggtgcataatgaagcaaaggcactccctgtgcctgagcaa 2640

[Tet2]_Dnmt3a(+/-)_PTCL_1_(AF(X)s8) gcagctcaacagaggtatttggtgcataatgaagcaaaggcactccctgtgcctgagcaa 2640

[Tet2]_Dnmt3a(+/-)_PTCL_2_(AF%(XI)x2) gcagctcaacagaggtatttggtgcataatgaagcaaaggcactccctgtgcctgagcaa 2640

[Tet2]_Dnmt3a(-/-)_PTCL_1_(AF(IX)d4) gcagctcaacagaggtatttggtgcataatgaagcaaaggcactccctgtgcctgagcaa 2640

[Tet2]_Dnmt3a(-/-)_PTCL_2_(AF(IX)i4) gcagctcaacagaggtatttggtgcataatgaagcaaaggcactccctgtgcctgagcaa 2640

************************************************************

[Tet2]_Dnmt3a(+/+)_WT_CD8_Reference ggaggaagtcagacacagacccctcctcagaaggacactcagaagcacgctgccttaagg 2700

[Tet2]_Dnmt3a(+/-)_PTCL_1_(AF(X)s8) ggaggaagtcagacacagacccctcctcagaaggacactcagaagcacgctgccttaagg 2700

[Tet2]_Dnmt3a(+/-)_PTCL_2_(AF%(XI)x2) ggaggaagtcagacacagacccctcctcagaaggacactcagaagcacgctgccttaagg 2700

[Tet2]_Dnmt3a(-/-)_PTCL_1_(AF(IX)d4) ggaggaagtcagacacagacccctcctcagaaggacactcagaagcacgctgccttaagg 2700

[Tet2]_Dnmt3a(-/-)_PTCL_2_(AF(IX)i4) ggaggaagtcagacacagacccctcctcagaaggacactcagaagcacgctgccttaagg 2700

************************************************************

[Tet2]_Dnmt3a(+/+)_WT_CD8_Reference tggcttctcttacagaagcaagaacagcagcaaacacagcaatcccagcctggtcataac 2760

[Tet2]_Dnmt3a(+/-)_PTCL_1_(AF(X)s8) tggcttctcttacagaagcaagaacagcagcaaacacagcaatcccagcctggtcataac 2760

[Tet2]_Dnmt3a(+/-)_PTCL_2_(AF%(XI)x2) tggcttctcttacagaagcaagaacagcagcaaacacagcaatcccagcctggtcataac 2760

[Tet2]_Dnmt3a(-/-)_PTCL_1_(AF(IX)d4) tggcttctcttacagaagcaagaacagcagcaaacacagcaatcccagcctggtcataac 2760

[Tet2]_Dnmt3a(-/-)_PTCL_2_(AF(IX)i4) tggcttctcttacagaagcaagaacagcagcaaacacagcaatcccagcctggtcataac 2760

************************************************************

[Tet2]_Dnmt3a(+/+)_WT_CD8_Reference cagatgcttaggccaatcaagactgagcctgtatccaaaccttcttcctatagatacccc 2820

[Tet2]_Dnmt3a(+/-)_PTCL_1_(AF(X)s8) cagatgcttaggccaatcaagactgagcctgtatccaaaccttcttcctatagatacccc 2820

[Tet2]_Dnmt3a(+/-)_PTCL_2_(AF%(XI)x2) cagatgcttaggccaatcaagactgagcctgtatccaaaccttcttcctatagatacccc 2820

[Tet2]_Dnmt3a(-/-)_PTCL_1_(AF(IX)d4) cagatgcttaggccaatcaagactgagcctgtatccaaaccttcttcctatagatacccc 2820

[Tet2]_Dnmt3a(-/-)_PTCL_2_(AF(IX)i4) cagatgcttaggccaatcaagactgagcctgtatccaaaccttcttcctatagatacccc 2820

************************************************************

[Tet2]_Dnmt3a(+/+)_WT_CD8_Reference ttgtcaccgccacaagaaaatatgtccagcaggataaagcaagagatctcctctccaagc 2880

[Tet2]_Dnmt3a(+/-)_PTCL_1_(AF(X)s8) ttgtcaccgccacaagaaaatatgtccagcaggataaagcaagagatctcctctccaagc 2880

[Tet2]_Dnmt3a(+/-)_PTCL_2_(AF%(XI)x2) ttgtcaccgccacaagaaaatatgtccagcaggataaagcaagagatctcctctccaagc 2880

[Tet2]_Dnmt3a(-/-)_PTCL_1_(AF(IX)d4) ttgtcaccgccacaagaaaatatgtccagcaggataaagcaagagatctcctctccaagc 2880

[Tet2]_Dnmt3a(-/-)_PTCL_2_(AF(IX)i4) ttgtcaccgccacaagaaaatatgtccagcaggataaagcaagagatctcctctccaagc 2880

************************************************************

[Tet2]_Dnmt3a(+/+)_WT_CD8_Reference cgtgacaatgggcagccaaagagcatcattgagaccatggaacagcacctgaagcagttt 2940

[Tet2]_Dnmt3a(+/-)_PTCL_1_(AF(X)s8) cgtgacaatgggcagccaaagagcatcattgagaccatggaacagcacctgaagcagttt 2940

[Tet2]_Dnmt3a(+/-)_PTCL_2_(AF%(XI)x2) cgtgacaatgggcagccaaagagcatcattgagaccatggaacagcacctgaagcagttt 2940

[Tet2]_Dnmt3a(-/-)_PTCL_1_(AF(IX)d4) cgtgacaatgggcagccaaagagcatcattgagaccatggaacagcacctgaagcagttt 2940

[Tet2]_Dnmt3a(-/-)_PTCL_2_(AF(IX)i4) cgtgacaatgggcagccaaagagcatcattgagaccatggaacagcacctgaagcagttt 2940

************************************************************

[Tet2]_Dnmt3a(+/+)_WT_CD8_Reference cagctcaagtcactctgtgactataaggctctgactctcaagtcacagaaacacgtgaaa 3000

[Tet2]_Dnmt3a(+/-)_PTCL_1_(AF(X)s8) cagctcaagtcactctgtgactataaggctctgactctcaagtcacagaaacacgtgaaa 3000

[Tet2]_Dnmt3a(+/-)_PTCL_2_(AF%(XI)x2) cagctcaagtcactctgtgactataaggctctgactctcaagtcacagaaacacgtgaaa 3000

[Tet2]_Dnmt3a(-/-)_PTCL_1_(AF(IX)d4) cagctcaagtcactctgtgactataaggctctgactctcaagtcacagaaacacgtgaaa 3000

[Tet2]_Dnmt3a(-/-)_PTCL_2_(AF(IX)i4) cagctcaagtcactctgtgactataaggctctgactctcaagtcacagaaacacgtgaaa 3000

************************************************************

[Tet2]_Dnmt3a(+/+)_WT_CD8_Reference gtgccaacagatatccaggctgcagaatcggagaaccacgcccgagctgcagagcctcaa 3060

[Tet2]_Dnmt3a(+/-)_PTCL_1_(AF(X)s8) gtgccaacagatatccaggctgcagaatcggagaaccacgcccgagctgcagagcctcaa 3060

[Tet2]_Dnmt3a(+/-)_PTCL_2_(AF%(XI)x2) gtgccaacagatatccaggctgcagaatcggagaaccacgcccgagctgcagagcctcaa 3060

[Tet2]_Dnmt3a(-/-)_PTCL_1_(AF(IX)d4) gtgccaacagatatccaggctgcagaatcggagaaccacgcccgagctgcagagcctcaa 3060

[Tet2]_Dnmt3a(-/-)_PTCL_2_(AF(IX)i4) gtgccaacagatatccaggctgcagaatcggagaaccacgcccgagctgcagagcctcaa 3060

************************************************************

[Tet2]_Dnmt3a(+/+)_WT_CD8_Reference gcaaccaaaagcacagattgttctgttctcgacgatgtttcagaatcagatactcctggt 3120

[Tet2]_Dnmt3a(+/-)_PTCL_1_(AF(X)s8) gcaaccaaaagcacagattgttctgttctcgacgatgtttcagaatcagatactcctggt 3120

[Tet2]_Dnmt3a(+/-)_PTCL_2_(AF%(XI)x2) gcaaccaaaagcacagattgttctgttctcgacgatgtttcagaatcagatactcctggt 3120

[Tet2]_Dnmt3a(-/-)_PTCL_1_(AF(IX)d4) gcaaccaaaagcacagattgttctgttctcgacgatgtttcagaatcagatactcctggt 3120

[Tet2]_Dnmt3a(-/-)_PTCL_2_(AF(IX)i4) gcaaccaaaagcacagattgttctgttctcgacgatgtttcagaatcagatactcctggt 3120

************************************************************

[Tet2]_Dnmt3a(+/+)_WT_CD8_Reference gaacaaagtcagaatggcaaatgtgaaggatgcaatccagacaaagatgaagctccttat 3180

[Tet2]_Dnmt3a(+/-)_PTCL_1_(AF(X)s8) gaacaaagtcagaatggcaaatgtgaaggatgcaatccagacaaagatgaagctccttat 3180

[Tet2]_Dnmt3a(+/-)_PTCL_2_(AF%(XI)x2) gaacaaagtcagaatggcaaatgtgaaggatgcaatccagacaaagatgaagctccttat 3180

[Tet2]_Dnmt3a(-/-)_PTCL_1_(AF(IX)d4) gaacaaagtcagaatggcaaatgtgaaggatgcaatccagacaaagatgaagctccttat 3180

[Tet2]_Dnmt3a(-/-)_PTCL_2_(AF(IX)i4) gaacaaagtcagaatggcaaatgtgaaggatgcaatccagacaaagatgaagctccttat 3180

************************************************************

[Tet2]_Dnmt3a(+/+)_WT_CD8_Reference tatacccatctaggagctggtcctgatgtggcagctattagaacactcatggaagaaagg 3240

[Tet2]_Dnmt3a(+/-)_PTCL_1_(AF(X)s8) tatacccatctaggagctggtcctgatgtggcagctattagaacactcatggaagaaagg 3240

[Tet2]_Dnmt3a(+/-)_PTCL_2_(AF%(XI)x2) tatacccatctaggagctggtcctgatgtggcagctattagaacactcatggaagaaagg 3240

[Tet2]_Dnmt3a(-/-)_PTCL_1_(AF(IX)d4) tatacccatctaggagctggtcctgatgtggcagctattagaacactcatggaagaaagg 3240

[Tet2]_Dnmt3a(-/-)_PTCL_2_(AF(IX)i4) tatacccatctaggagctggtcctgatgtggcagctattagaacactcatggaagaaagg 3240

************************************************************

[Tet2]_Dnmt3a(+/+)_WT_CD8_Reference tatggagagaagggtaaagctattaggattgaaaaagtcatatatactggtaaagaaggc 3300

[Tet2]_Dnmt3a(+/-)_PTCL_1_(AF(X)s8) tatggagagaagggtaaagctattaggattgaaaaagtcatatatactggtaaagaaggc 3300

[Tet2]_Dnmt3a(+/-)_PTCL_2_(AF%(XI)x2) tatggagagaagggtaaagctattaggattgaaaaagtcatatatactggtaaagaaggc 3300

[Tet2]_Dnmt3a(-/-)_PTCL_1_(AF(IX)d4) tatggagagaagggtaaagctattaggattgaaaaagtcatatatactggtaaagaaggc 3300

[Tet2]_Dnmt3a(-/-)_PTCL_2_(AF(IX)i4) tatggagagaagggtaaagctattaggattgaaaaagtcatatatactggtaaagaaggc 3300

************************************************************

[Tet2]_Dnmt3a(+/+)_WT_CD8_Reference aagagctctcagggatgtcctattgctaaatgggtatatcggagatcgagtgaggaggag 3360

[Tet2]_Dnmt3a(+/-)_PTCL_1_(AF(X)s8) aagagctctcagggatgtcctattgctaaatgggtatatcggagatcgagtgaggaggag 3360

[Tet2]_Dnmt3a(+/-)_PTCL_2_(AF%(XI)x2) aagagctctcagggatgtcctattgctaaatgggtatatcggagatcgagtgaggaggag 3360

[Tet2]_Dnmt3a(-/-)_PTCL_1_(AF(IX)d4) aagagctctcagggatgtcctattgctaaatgggtatatcggagatcgagtgaggaggag 3360

[Tet2]_Dnmt3a(-/-)_PTCL_2_(AF(IX)i4) aagagctctcagggatgtcctattgctaaatgggtatatcggagatcgagtgaggaggag 3360

************************************************************

[Tet2]_Dnmt3a(+/+)_WT_CD8_Reference aaactactgtgtttggtacgagtgcgacctaatcacacatgtgagacggcggtgatggta 3420

[Tet2]_Dnmt3a(+/-)_PTCL_1_(AF(X)s8) aaactactgtgtttggtacgagtgcgacctaatcacacatgtgagacggcggtgatggta 3420

[Tet2]_Dnmt3a(+/-)_PTCL_2_(AF%(XI)x2) aaactactgtgtttggtacgagtgcgacctaatcacacatgtgagacggcggtgatggta 3420

[Tet2]_Dnmt3a(-/-)_PTCL_1_(AF(IX)d4) aaactactgtgtttggtacgagtgcgacctaatcacacatgtgagacggcggtgatggta 3420

[Tet2]_Dnmt3a(-/-)_PTCL_2_(AF(IX)i4) aaactactgtgtttggtacgagtgcgacctaatcacacatgtgagacggcggtgatggta 3420

************************************************************

[Tet2]_Dnmt3a(+/+)_WT_CD8_Reference attgccatcatgttgtgggacggaatcccaaagctactcgcatcagaactctactcagaa 3480

[Tet2]_Dnmt3a(+/-)_PTCL_1_(AF(X)s8) attgccatcatgttgtgggacggaatcccaaagctactcgcatcagaactctactcagaa 3480

[Tet2]_Dnmt3a(+/-)_PTCL_2_(AF%(XI)x2) attgccatcatgttgtgggacggaatcccaaagctactcgcatcagaactctactcagaa 3480

[Tet2]_Dnmt3a(-/-)_PTCL_1_(AF(IX)d4) attgccatcatgttgtgggacggaatcccaaagctactcgcatcagaactctactcagaa 3480

[Tet2]_Dnmt3a(-/-)_PTCL_2_(AF(IX)i4) attgccatcatgttgtgggacggaatcccaaagctactcgcatcagaactctactcagaa 3480

************************************************************

[Tet2]_Dnmt3a(+/+)_WT_CD8_Reference cttacagatatcttgggcaagtgtggcatatgcaccaaccgtcgctgttctcagaatgaa 3540

[Tet2]_Dnmt3a(+/-)_PTCL_1_(AF(X)s8) cttacagatatcttgggcaagtgtggcatatgcaccaaccgtcgctgttctcagaatgaa 3540

[Tet2]_Dnmt3a(+/-)_PTCL_2_(AF%(XI)x2) cttacagatatcttgggcaagtgtggcatatgcaccaaccgtcgctgttctcagaatgaa 3540

[Tet2]_Dnmt3a(-/-)_PTCL_1_(AF(IX)d4) cttacagatatcttgggcaagtgtggcatatgcaccaaccgtcgctgttctcagaatgaa 3540

[Tet2]_Dnmt3a(-/-)_PTCL_2_(AF(IX)i4) cttacagatatcttgggcaagtgtggcatatgcaccaaccgtcgctgttctcagaatgaa 3540

************************************************************

[Tet2]_Dnmt3a(+/+)_WT_CD8_Reference actagaaactgttgttgtcagggtgagaatccagagacctgtggtgcctccttttctttt 3600

[Tet2]_Dnmt3a(+/-)_PTCL_1_(AF(X)s8) actagaaactgttgttgtcagggtgagaatccagagacctgtggtgcctccttttctttt 3600

[Tet2]_Dnmt3a(+/-)_PTCL_2_(AF%(XI)x2) actagaaactgttgttgtcagggtgagaatccagagacctgtggtgcctccttttctttt 3600

[Tet2]_Dnmt3a(-/-)_PTCL_1_(AF(IX)d4) actagaaactgttgttgtcagggtgagaatccagagacctgtggtgcctccttttctttt 3600

[Tet2]_Dnmt3a(-/-)_PTCL_2_(AF(IX)i4) actagaaactgttgttgtcagggtgagaatccagagacctgtggtgcctccttttctttt 3600

************************************************************

[Tet2]_Dnmt3a(+/+)_WT_CD8_Reference ggttgttcttggagcatgtactataatggatgtaagtttgccagaagcaagaaaccaagg 3660

[Tet2]_Dnmt3a(+/-)_PTCL_1_(AF(X)s8) ggttgttcttggagcatgtactataatggatgtaagtttgccagaagcaagaaaccaagg 3660

[Tet2]_Dnmt3a(+/-)_PTCL_2_(AF%(XI)x2) ggttgttcttggagcatgtactataatggatgtaagtttgccagaagcaagaaaccaagg 3660

[Tet2]_Dnmt3a(-/-)_PTCL_1_(AF(IX)d4) ggttgttcttggagcatgtactataatggatgtaagtttgccagaagcaagaaaccaagg 3660

[Tet2]_Dnmt3a(-/-)_PTCL_2_(AF(IX)i4) ggttgttcttggagcatgtactataatggatgtaagtttgccagaagcaagaaaccaagg 3660

************************************************************

[Tet2]_Dnmt3a(+/+)_WT_CD8_Reference aaatttaggctacatggagctgagccaaaagaggaagagagactaggttctcatttgcaa 3720

[Tet2]_Dnmt3a(+/-)_PTCL_1_(AF(X)s8) aaatttaggctacatggagctgagccaaaagaggaagagagactaggttctcatttgcaa 3720

[Tet2]_Dnmt3a(+/-)_PTCL_2_(AF%(XI)x2) aaatttaggctacatggagctgagccaaaagaggaagagagactaggttctcatttgcaa 3720

[Tet2]_Dnmt3a(-/-)_PTCL_1_(AF(IX)d4) aaatttaggctacatggagctgagccaaaagaggaagagagactaggttctcatttgcaa 3720

[Tet2]_Dnmt3a(-/-)_PTCL_2_(AF(IX)i4) aaatttaggctacatggagctgagccaaaagaggaagagagactaggttctcatttgcaa 3720

************************************************************

[Tet2]_Dnmt3a(+/+)_WT_CD8_Reference aacctggctactgtcattgctccaatatacaagaagcttgcacccgatgcatacaataat 3780

[Tet2]_Dnmt3a(+/-)_PTCL_1_(AF(X)s8) aacctggctactgtcattgctccaatatacaagaagcttgcacccgatgcatacaataat 3780

[Tet2]_Dnmt3a(+/-)_PTCL_2_(AF%(XI)x2) aacctggctactgtcattgctccaatatacaagaagcttgcacccgatgcatacaataat 3780

[Tet2]_Dnmt3a(-/-)_PTCL_1_(AF(IX)d4) aacctggctactgtcattgctccaatatacaagaagcttgcacccgatgcatacaataat 3780

[Tet2]_Dnmt3a(-/-)_PTCL_2_(AF(IX)i4) aacctggctactgtcattgctccaatatacaagaagcttgcacccgatgcatacaataat 3780

************************************************************

[Tet2]_Dnmt3a(+/+)_WT_CD8_Reference caggttgaatttgaacaccaagccccagactgctgtttgggtctgaaggaaggccggcca 3840

[Tet2]_Dnmt3a(+/-)_PTCL_1_(AF(X)s8) caggttgaatttgaacaccaagccccagactgctgtttgggtctgaaggaaggccggcca 3840

[Tet2]_Dnmt3a(+/-)_PTCL_2_(AF%(XI)x2) caggttgaatttgaacaccaagccccagactgctgtttgggtctgaaggaaggccggcca 3840

[Tet2]_Dnmt3a(-/-)_PTCL_1_(AF(IX)d4) caggttgaatttgaacaccaagccccagactgctgtttgggtctgaaggaaggccggcca 3840

[Tet2]_Dnmt3a(-/-)_PTCL_2_(AF(IX)i4) caggttgaatttgaacaccaagccccagactgctgtttgggtctgaaggaaggccggcca 3840

************************************************************

[Tet2]_Dnmt3a(+/+)_WT_CD8_Reference ttctcaggagtcactgcatgtttggacttctctgctcattcccacagagaccagcagaac 3900

[Tet2]_Dnmt3a(+/-)_PTCL_1_(AF(X)s8) ttctcaggagtcactgcatgtttggacttctctgctcattcccacagagaccagcagaac 3900

[Tet2]_Dnmt3a(+/-)_PTCL_2_(AF%(XI)x2) ttctcaggagtcactgcatgtttggacttctctgctcattcccacagagaccagcagaac 3900

[Tet2]_Dnmt3a(-/-)_PTCL_1_(AF(IX)d4) ttctcaggagtcactgcatgtttggacttctctgctcattcccacagagaccagcagaac 3900

[Tet2]_Dnmt3a(-/-)_PTCL_2_(AF(IX)i4) ttctcaggagtcactgcatgtttggacttctctgctcattcccacagagaccagcagaac 3900

************************************************************

[Tet2]_Dnmt3a(+/+)_WT_CD8_Reference atgccaaatggcagtacagtggtggtcaccctcaatagagaagacaatcgagaagtcgga 3960

[Tet2]_Dnmt3a(+/-)_PTCL_1_(AF(X)s8) atgccaaatggcagtacagtggtggtcaccctcaatagagaagacaatcgagaagtcgga 3960

[Tet2]_Dnmt3a(+/-)_PTCL_2_(AF%(XI)x2) atgccaaatggcagtacagtggtggtcaccctcaatagagaagacaatcgagaagtcgga 3960

[Tet2]_Dnmt3a(-/-)_PTCL_1_(AF(IX)d4) atgccaaatggcagtacagtggtggtcaccctcaatagagaagacaatcgagaagtcgga 3960

[Tet2]_Dnmt3a(-/-)_PTCL_2_(AF(IX)i4) atgccaaatggcagtacagtggtggtcaccctcaatagagaagacaatcgagaagtcgga 3960

************************************************************

[Tet2]_Dnmt3a(+/+)_WT_CD8_Reference gctaagcctgaggatgagcagttccacgtgctgcctatgtacatcatcgcccctgaggat 4020

[Tet2]_Dnmt3a(+/-)_PTCL_1_(AF(X)s8) gctaagcctgaggatgagcagttccacgtgctgcctatgtacatcatcgcccctgaggat 4020

[Tet2]_Dnmt3a(+/-)_PTCL_2_(AF%(XI)x2) gctaagcctgaggatgagcagttccacgtgctgcctatgtacatcatcgcccctgaggat 4020

[Tet2]_Dnmt3a(-/-)_PTCL_1_(AF(IX)d4) gctaagcctgaggatgagcagttccacgtgctgcctatgtacatcatcgcccctgaggat 4020

[Tet2]_Dnmt3a(-/-)_PTCL_2_(AF(IX)i4) gctaagcctgaggatgagcagttccacgtgctgcctatgtacatcatcgcccctgaggat 4020

************************************************************

[Tet2]_Dnmt3a(+/+)_WT_CD8_Reference gagtttgggagtacggaaggccaggagaagaagatacggatggggtccattgaggttctg 4080

[Tet2]_Dnmt3a(+/-)_PTCL_1_(AF(X)s8) gagtttgggagtacggaaggccaggagaagaagatacggatggggtccattgaggttctg 4080

[Tet2]_Dnmt3a(+/-)_PTCL_2_(AF%(XI)x2) gagtttgggagtacggaaggccaggagaagaagatacggatggggtccattgaggttctg 4080

[Tet2]_Dnmt3a(-/-)_PTCL_1_(AF(IX)d4) gagtttgggagtacggaaggccaggagaagaagatacggatggggtccattgaggttctg 4080

[Tet2]_Dnmt3a(-/-)_PTCL_2_(AF(IX)i4) gagtttgggagtacggaaggccaggagaagaagatacggatggggtccattgaggttctg 4080

************************************************************

[Tet2]_Dnmt3a(+/+)_WT_CD8_Reference cagtcatttcggaggagaagggtcataaggataggagagctgcccaagagttgcaagaag 4140

[Tet2]_Dnmt3a(+/-)_PTCL_1_(AF(X)s8) cagtcatttcggaggagaagggtcataaggataggagagctgcccaagagttgcaagaag 4140

[Tet2]_Dnmt3a(+/-)_PTCL_2_(AF%(XI)x2) cagtcatttcggaggagaagggtcataaggataggagagctgcccaagagttgcaagaag 4140

[Tet2]_Dnmt3a(-/-)_PTCL_1_(AF(IX)d4) cagtcatttcggaggagaagggtcataaggataggagagctgcccaagagttgcaagaag 4140

[Tet2]_Dnmt3a(-/-)_PTCL_2_(AF(IX)i4) cagtcatttcggaggagaagggtcataaggataggagagctgcccaagagttgcaagaag 4140

************************************************************

[Tet2]_Dnmt3a(+/+)_WT_CD8_Reference aaagcggagcccaagaaagccaagaccaagaaagcagctcgaaagcgttcctctctggag 4200

[Tet2]_Dnmt3a(+/-)_PTCL_1_(AF(X)s8) aaagcggagcccaagaaagccaagaccaagaaagcagctcgaaagcgttcctctctggag 4200

[Tet2]_Dnmt3a(+/-)_PTCL_2_(AF%(XI)x2) aaagcggagcccaagaaagccaagaccaagaaagcagctcgaaagcgttcctctctggag 4200

[Tet2]_Dnmt3a(-/-)_PTCL_1_(AF(IX)d4) aaagcggagcccaagaaagccaagaccaagaaagcagctcgaaagcgttcctctctggag 4200

[Tet2]_Dnmt3a(-/-)_PTCL_2_(AF(IX)i4) aaagcggagcccaagaaagccaagaccaagaaagcagctcgaaagcgttcctctctggag 4200

************************************************************

[Tet2]_Dnmt3a(+/+)_WT_CD8_Reference aactgctccagtaggactgagaagggaaagtcttcctcacatacaaagctgatggaaaat 4260

[Tet2]_Dnmt3a(+/-)_PTCL_1_(AF(X)s8) aactgctccagtaggactgagaagggaaagtcttcctcacatacaaagctgatggaaaat 4260

[Tet2]_Dnmt3a(+/-)_PTCL_2_(AF%(XI)x2) aactgctccagtaggactgagaagggaaagtcttcctcacatacaaagctgatggaaaat 4260

[Tet2]_Dnmt3a(-/-)_PTCL_1_(AF(IX)d4) aactgctccagtaggactgagaagggaaagtcttcctcacatacaaagctgatggaaaat 4260

[Tet2]_Dnmt3a(-/-)_PTCL_2_(AF(IX)i4) aactgctccagtaggactgagaagggaaagtcttcctcacatacaaagctgatggaaaat 4260

************************************************************

[Tet2]_Dnmt3a(+/+)_WT_CD8_Reference gcaagccatatgaaacaaatgacagcacaaccgcagctttcgggcccggtcatccggcag 4320

[Tet2]_Dnmt3a(+/-)_PTCL_1_(AF(X)s8) gcaagccatatgaaacaaatgacagcacaaccgcagctttcgggcccggtcatccggcag 4320

[Tet2]_Dnmt3a(+/-)_PTCL_2_(AF%(XI)x2) gcaagccatatgaaacaaatgacagcacaaccgcagctttcgggcccggtcatccggcag 4320

[Tet2]_Dnmt3a(-/-)_PTCL_1_(AF(IX)d4) gcaagccatatgaaacaaatgacagcacaaccgcagctttcgggcccggtcatccggcag 4320

[Tet2]_Dnmt3a(-/-)_PTCL_2_(AF(IX)i4) gcaagccatatgaaacaaatgacagcacaaccgcagctttcgggcccggtcatccggcag 4320

************************************************************

[Tet2]_Dnmt3a(+/+)_WT_CD8_Reference ccaccaacactccagaggcaccttcagcaagggcagaggccacagcagccgcagccacct 4380

[Tet2]_Dnmt3a(+/-)_PTCL_1_(AF(X)s8) ccaccaacactccagaggcaccttcagcaagggcagaggccacagcagccgcagccacct 4380

[Tet2]_Dnmt3a(+/-)_PTCL_2_(AF%(XI)x2) ccaccaacactccagaggcaccttcagcaagggcagaggccacagcagccgcagccacct 4380

[Tet2]_Dnmt3a(-/-)_PTCL_1_(AF(IX)d4) ccaccaacactccagaggcaccttcagcaagggcagaggccacagcagccgcagccacct 4380

[Tet2]_Dnmt3a(-/-)_PTCL_2_(AF(IX)i4) ccaccaacactccagaggcaccttcagcaagggcagaggccacagcagccgcagccacct 4380

************************************************************

[Tet2]_Dnmt3a(+/+)_WT_CD8_Reference cagccgcagccgcagacgacacctcagccacagccacagccacagcatatcatgcccggt 4440

[Tet2]_Dnmt3a(+/-)_PTCL_1_(AF(X)s8) cagccgcagccgcagacgacacctcagccacagccacagccacagcatatcatgcccggt 4440

[Tet2]_Dnmt3a(+/-)_PTCL_2_(AF%(XI)x2) cagccgcagccgcagacgacacctcagccacagccacagccacagcatatcatgcccggt 4440

[Tet2]_Dnmt3a(-/-)_PTCL_1_(AF(IX)d4) cagccgcagccgcagacgacacctcagccacagccacagccacagcatatcatgcccggt 4440

[Tet2]_Dnmt3a(-/-)_PTCL_2_(AF(IX)i4) cagccgcagccgcagacgacacctcagccacagccacagccacagcatatcatgcccggt 4440

************************************************************

[Tet2]_Dnmt3a(+/+)_WT_CD8_Reference aactctcagtctgttggttctcattgttctggatccaccagtgtctacacgagacagcct 4500

[Tet2]_Dnmt3a(+/-)_PTCL_1_(AF(X)s8) aactctcagtctgttggttctcattgttctggatccaccagtgtctacacgagacagcct 4500

[Tet2]_Dnmt3a(+/-)_PTCL_2_(AF%(XI)x2) aactctcagtctgttggttctcattgttctggatccaccagtgtctacacgagacagcct 4500

[Tet2]_Dnmt3a(-/-)_PTCL_1_(AF(IX)d4) aactctcagtctgttggttctcattgttctggatccaccagtgtctacacgagacagcct 4500

[Tet2]_Dnmt3a(-/-)_PTCL_2_(AF(IX)i4) aactctcagtctgttggttctcattgttctggatccaccagtgtctacacgagacagcct 4500

************************************************************

[Tet2]_Dnmt3a(+/+)_WT_CD8_Reference actcctcacagtccttatcccagctcagcacacacctcagatatttatggagataccaac 4560

[Tet2]_Dnmt3a(+/-)_PTCL_1_(AF(X)s8) actcctcacagtccttatcccagctcagcacacacctcagatatttatggagataccaac 4560

[Tet2]_Dnmt3a(+/-)_PTCL_2_(AF%(XI)x2) actcctcacagtccttatcccagctcagcacacacctcagatatttatggagataccaac 4560

[Tet2]_Dnmt3a(-/-)_PTCL_1_(AF(IX)d4) actcctcacagtccttatcccagctcagcacacacctcagatatttatggagataccaac 4560

[Tet2]_Dnmt3a(-/-)_PTCL_2_(AF(IX)i4) actcctcacagtccttatcccagctcagcacacacctcagatatttatggagataccaac 4560

************************************************************

[Tet2]_Dnmt3a(+/+)_WT_CD8_Reference catgtgaacttttaccccacttcatctcatgcctcgggttcatatttgaatccttctaat 4620

[Tet2]_Dnmt3a(+/-)_PTCL_1_(AF(X)s8) catgtgaacttttaccccacttcatctcatgcctcgggttcatatttgaatccttctaat 4620

[Tet2]_Dnmt3a(+/-)_PTCL_2_(AF%(XI)x2) catgtgaacttttaccccacttcatctcatgcctcgggttcatatttgaatccttctaat 4620

[Tet2]_Dnmt3a(-/-)_PTCL_1_(AF(IX)d4) catgtgaacttttaccccacttcatctcatgcctcgggttcatatttgaatccttctaat 4620

[Tet2]_Dnmt3a(-/-)_PTCL_2_(AF(IX)i4) catgtgaacttttaccccacttcatctcatgcctcgggttcatatttgaatccttctaat 4620

************************************************************

[Tet2]_Dnmt3a(+/+)_WT_CD8_Reference tacatgaacccctaccttgggcttttgaatcagaataaccaatatgcaccttttccatac 4680

[Tet2]_Dnmt3a(+/-)_PTCL_1_(AF(X)s8) tacatgaacccctaccttgggcttttgaatcagaataaccaatatgcaccttttccatac 4680

[Tet2]_Dnmt3a(+/-)_PTCL_2_(AF%(XI)x2) tacatgaacccctaccttgggcttttgaatcagaataaccaatatgcaccttttccatac 4680

[Tet2]_Dnmt3a(-/-)_PTCL_1_(AF(IX)d4) tacatgaacccctaccttgggcttttgaatcagaataaccaatatgcaccttttccatac 4680

[Tet2]_Dnmt3a(-/-)_PTCL_2_(AF(IX)i4) tacatgaacccctaccttgggcttttgaatcagaataaccaatatgcaccttttccatac 4680

************************************************************

[Tet2]_Dnmt3a(+/+)_WT_CD8_Reference aatgggagtgtgccagtggacaatggttcccctttcttaggttcttattccccccaggct 4740

[Tet2]_Dnmt3a(+/-)_PTCL_1_(AF(X)s8) aatgggagtgtgccagtggacaatggttcccctttcttaggttcttattccccccaggct 4740

[Tet2]_Dnmt3a(+/-)_PTCL_2_(AF%(XI)x2) aatgggagtgtgccagtggacaatggttcccctttcttaggttcttattccccccaggct 4740

[Tet2]_Dnmt3a(-/-)_PTCL_1_(AF(IX)d4) aatgggagtgtgccagtggacaatggttcccctttcttaggttcttattccccccaggct 4740

[Tet2]_Dnmt3a(-/-)_PTCL_2_(AF(IX)i4) aatgggagtgtgccagtggacaatggttcccctttcttaggttcttattccccccaggct 4740

************************************************************

[Tet2]_Dnmt3a(+/+)_WT_CD8_Reference cagtccagggatctacatagatatccaaaccaggaccatctcaccaatcagaacttacca 4800

[Tet2]_Dnmt3a(+/-)_PTCL_1_(AF(X)s8) cagtccagggatctacatagatatccaaaccaggaccatctcaccaatcagaacttacca 4800

[Tet2]_Dnmt3a(+/-)_PTCL_2_(AF%(XI)x2) cagtccagggatctacatagatatccaaaccaggaccatctcaccaatcagaacttacca 4800

[Tet2]_Dnmt3a(-/-)_PTCL_1_(AF(IX)d4) cagtccagggatctacatagatatccaaaccaggaccatctcaccaatcagaacttacca 4800

[Tet2]_Dnmt3a(-/-)_PTCL_2_(AF(IX)i4) cagtccagggatctacatagatatccaaaccaggaccatctcaccaatcagaacttacca 4800

************************************************************

[Tet2]_Dnmt3a(+/+)_WT_CD8_Reference cccatccacacccttcaccaacagacgtttggggacagtccctctaagtacttaagttat 4860

[Tet2]_Dnmt3a(+/-)_PTCL_1_(AF(X)s8) cccatccacacccttcaccaacagacgtttggggacagtccctctaagtacttaagttat 4860

[Tet2]_Dnmt3a(+/-)_PTCL_2_(AF%(XI)x2) cccatccacacccttcaccaacagacgtttggggacagtccctctaagtacttaagttat 4860

[Tet2]_Dnmt3a(-/-)_PTCL_1_(AF(IX)d4) cccatccacacccttcaccaacagacgtttggggacagtccctctaagtacttaagttat 4860

[Tet2]_Dnmt3a(-/-)_PTCL_2_(AF(IX)i4) cccatccacacccttcaccaacagacgtttggggacagtccctctaagtacttaagttat 4860

************************************************************

[Tet2]_Dnmt3a(+/+)_WT_CD8_Reference ggaaaccaaaatatgcagagagatgccttcactactaactccaccctaaaaccaaatgta 4920

[Tet2]_Dnmt3a(+/-)_PTCL_1_(AF(X)s8) ggaaaccaaaatatgcagagagatgccttcactactaactccaccctaaaaccaaatgta 4920

[Tet2]_Dnmt3a(+/-)_PTCL_2_(AF%(XI)x2) ggaaaccaaaatatgcagagagatgccttcactactaactccaccctaaaaccaaatgta 4920

[Tet2]_Dnmt3a(-/-)_PTCL_1_(AF(IX)d4) ggaaaccaaaatatgcagagagatgccttcactactaactccaccctaaaaccaaatgta 4920

[Tet2]_Dnmt3a(-/-)_PTCL_2_(AF(IX)i4) ggaaaccaaaatatgcagagagatgccttcactactaactccaccctaaaaccaaatgta 4920

************************************************************

[Tet2]_Dnmt3a(+/+)_WT_CD8_Reference caccacctagcaacgttttctccttaccccacccccaagatggatagtcatttcatggga 4980

[Tet2]_Dnmt3a(+/-)_PTCL_1_(AF(X)s8) caccacctagcaacgttttctccttaccccacccccaagatggatagtcatttcatggga 4980

[Tet2]_Dnmt3a(+/-)_PTCL_2_(AF%(XI)x2) caccacctagcaacgttttctccttaccccacccccaagatggatagtcatttcatggga 4980

[Tet2]_Dnmt3a(-/-)_PTCL_1_(AF(IX)d4) caccacctagcaacgttttctccttaccccacccccaagatggatagtcatttcatggga 4980

[Tet2]_Dnmt3a(-/-)_PTCL_2_(AF(IX)i4) caccacctagcaacgttttctccttaccccacccccaagatggatagtcatttcatggga 4980

************************************************************

[Tet2]_Dnmt3a(+/+)_WT_CD8_Reference gctgcctccagatcaccatacagccacccacacactgactacaaaaccagtgagcatcat 5040

[Tet2]_Dnmt3a(+/-)_PTCL_1_(AF(X)s8) gctgcctccagatcaccatacagccacccacacactgactacaaaaccagtgagcatcat 5040

[Tet2]_Dnmt3a(+/-)_PTCL_2_(AF%(XI)x2) gctgcctccagatcaccatacagccacccacacactgactacaaaaccagtgagcatcat 5040

[Tet2]_Dnmt3a(-/-)_PTCL_1_(AF(IX)d4) gctgcctccagatcaccatacagccacccacacactgactacaaaaccagtgagcatcat 5040

[Tet2]_Dnmt3a(-/-)_PTCL_2_(AF(IX)i4) gctgcctccagatcaccatacagccacccacacactgactacaaaaccagtgagcatcat 5040

************************************************************

[Tet2]_Dnmt3a(+/+)_WT_CD8_Reference ctaccctctcacacgatctacagctacacggcagcagcttcggggagcagttccagccac 5100

[Tet2]_Dnmt3a(+/-)_PTCL_1_(AF(X)s8) ctaccctctcacacgatctacagctacacggcagcagcttcggggagcagttccagccac 5100

[Tet2]_Dnmt3a(+/-)_PTCL_2_(AF%(XI)x2) ctaccctctcacacgatctacagctacacggcagcagcttcggggagcagttccagccac 5100

[Tet2]_Dnmt3a(-/-)_PTCL_1_(AF(IX)d4) ctaccctctcacacgatctacagctacacggcagcagcttcggggagcagttccagccac 5100

[Tet2]_Dnmt3a(-/-)_PTCL_2_(AF(IX)i4) ctaccctctcacacgatctacagctacacggcagcagcttcggggagcagttccagccac 5100

************************************************************

[Tet2]_Dnmt3a(+/+)_WT_CD8_Reference gccttccacaacaaggagaatgacaacatagccaatgggctctcaagagtgcttccaggg 5160

[Tet2]_Dnmt3a(+/-)_PTCL_1_(AF(X)s8) gccttccacaacaaggagaatgacaacatagccaatgggctctcaagagtgcttccaggg 5160

[Tet2]_Dnmt3a(+/-)_PTCL_2_(AF%(XI)x2) gccttccacaacaaggagaatgacaacatagccaatgggctctcaagagtgcttccaggg 5160

[Tet2]_Dnmt3a(-/-)_PTCL_1_(AF(IX)d4) gccttccacaacaaggagaatgacaacatagccaatgggctctcaagagtgcttccaggg 5160

[Tet2]_Dnmt3a(-/-)_PTCL_2_(AF(IX)i4) gccttccacaacaaggagaatgacaacatagccaatgggctctcaagagtgcttccaggg 5160

************************************************************

[Tet2]_Dnmt3a(+/+)_WT_CD8_Reference tttaatcatgatagaactgcttctgcccaagaactattatacagtctgactggcagcagt 5220

[Tet2]_Dnmt3a(+/-)_PTCL_1_(AF(X)s8) tttaatcatgatagaactgcttctgcccaagaactattatacagtctgactggcagcagt 5220

[Tet2]_Dnmt3a(+/-)_PTCL_2_(AF%(XI)x2) tttaatcatgatagaactgcttctgcccaagaactattatacagtctgactggcagcagt 5220

[Tet2]_Dnmt3a(-/-)_PTCL_1_(AF(IX)d4) tttaatcatgatagaactgcttctgcccaagaactattatacagtctgactggcagcagt 5220

[Tet2]_Dnmt3a(-/-)_PTCL_2_(AF(IX)i4) tttaatcatgatagaactgcttctgcccaagaactattatacagtctgactggcagcagt 5220

************************************************************

[Tet2]_Dnmt3a(+/+)_WT_CD8_Reference caggagaagcagcctgaggtgtcaggccaggatgcagctgctgtgcaggaaattgagtat 5280

[Tet2]_Dnmt3a(+/-)_PTCL_1_(AF(X)s8) caggagaagcagcctgaggtgtcaggccaggatgcagctgctgtgcaggaaattgagtat 5280

[Tet2]_Dnmt3a(+/-)_PTCL_2_(AF%(XI)x2) caggagaagcagcctgaggtgtcaggccaggatgcagctgctgtgcaggaaattgagtat 5280

[Tet2]_Dnmt3a(-/-)_PTCL_1_(AF(IX)d4) caggagaagcagcctgaggtgtcaggccaggatgcagctgctgtgcaggaaattgagtat 5280

[Tet2]_Dnmt3a(-/-)_PTCL_2_(AF(IX)i4) caggagaagcagcctgaggtgtcaggccaggatgcagctgctgtgcaggaaattgagtat 5280

************************************************************

[Tet2]_Dnmt3a(+/+)_WT_CD8_Reference tggtcagatagtgagcacaactttcaggatccttgcattggaggggtggctatagctcca 5340

[Tet2]_Dnmt3a(+/-)_PTCL_1_(AF(X)s8) tggtcagatagtgagcacaactttcaggatccttgcattggaggggtggctatagctcca 5340

[Tet2]_Dnmt3a(+/-)_PTCL_2_(AF%(XI)x2) tggtcagatagtgagcacaactttcaggatccttgcattggaggggtggctatagctcca 5340

[Tet2]_Dnmt3a(-/-)_PTCL_1_(AF(IX)d4) tggtcagatagtgagcacaactttcaggatccttgcattggaggggtggctatagctcca 5340

[Tet2]_Dnmt3a(-/-)_PTCL_2_(AF(IX)i4) tggtcagatagtgagcacaactttcaggatccttgcattggaggggtggctatagctcca 5340

************************************************************

[Tet2]_Dnmt3a(+/+)_WT_CD8_Reference actcatgggtcaattcttattgagtgtgcaaagtgtgaggttcatgccacaaccaaagta 5400

[Tet2]_Dnmt3a(+/-)_PTCL_1_(AF(X)s8) actcatgggtcaattcttattgagtgtgcaaagtgtgaggttcatgccacaaccaaagta 5400

[Tet2]_Dnmt3a(+/-)_PTCL_2_(AF%(XI)x2) actcatgggtcaattcttattgagtgtgcaaagtgtgaggttcatgccacaaccaaagta 5400

[Tet2]_Dnmt3a(-/-)_PTCL_1_(AF(IX)d4) actcatgggtcaattcttattgagtgtgcaaagtgtgaggttcatgccacaaccaaagta 5400

[Tet2]_Dnmt3a(-/-)_PTCL_2_(AF(IX)i4) actcatgggtcaattcttattgagtgtgcaaagtgtgaggttcatgccacaaccaaagta 5400

************************************************************

[Tet2]_Dnmt3a(+/+)_WT_CD8_Reference aacgatcccgaccggaatcaccccaccaggatctcacttgtactgtataggcataagaat 5460

[Tet2]_Dnmt3a(+/-)_PTCL_1_(AF(X)s8) aacgatcccgaccggaatcaccccaccaggatctcacttgtactgtataggcataagaat 5460

[Tet2]_Dnmt3a(+/-)_PTCL_2_(AF%(XI)x2) aacgatcccgaccggaatcaccccaccaggatctcacttgtactgtataggcataagaat 5460

[Tet2]_Dnmt3a(-/-)_PTCL_1_(AF(IX)d4) aacgatcccgaccggaatcaccccaccaggatctcacttgtactgtataggcataagaat 5460

[Tet2]_Dnmt3a(-/-)_PTCL_2_(AF(IX)i4) aacgatcccgaccggaatcaccccaccaggatctcacttgtactgtataggcataagaat 5460

************************************************************

[Tet2]_Dnmt3a(+/+)_WT_CD8_Reference ttgtttctaccaaaacattgtttggctctctgggaagccaaaatggctgaaaaggcccgg 5520

[Tet2]_Dnmt3a(+/-)_PTCL_1_(AF(X)s8) ttgtttctaccaaaacattgtttggctctctgggaagccaaaatggctgaaaaggcccgg 5520

[Tet2]_Dnmt3a(+/-)_PTCL_2_(AF%(XI)x2) ttgtttctaccaaaacattgtttggctctctgggaagccaaaatggctgaaaaggcccgg 5520

[Tet2]_Dnmt3a(-/-)_PTCL_1_(AF(IX)d4) ttgtttctaccaaaacattgtttggctctctgggaagccaaaatggctgaaaaggcccgg 5520

[Tet2]_Dnmt3a(-/-)_PTCL_2_(AF(IX)i4) ttgtttctaccaaaacattgtttggctctctgggaagccaaaatggctgaaaaggcccgg 5520

************************************************************

[Tet2]_Dnmt3a(+/+)_WT_CD8_Reference aaagaggaagagtgcggaaagaatggatcagaccacgtgtctcagaaaaatcatggcaaa 5580

[Tet2]_Dnmt3a(+/-)_PTCL_1_(AF(X)s8) aaagaggaagagtgcggaaagaatggatcagaccacgtgtctcagaaaaatcatggcaaa 5580

[Tet2]_Dnmt3a(+/-)_PTCL_2_(AF%(XI)x2) aaagaggaagagtgcggaaagaatggatcagaccacgtgtctcagaaaaatcatggcaaa 5580

[Tet2]_Dnmt3a(-/-)_PTCL_1_(AF(IX)d4) aaagaggaagagtgcggaaagaatggatcagaccacgtgtctcagaaaaatcatggcaaa 5580

[Tet2]_Dnmt3a(-/-)_PTCL_2_(AF(IX)i4) aaagaggaagagtgcggaaagaatggatcagaccacgtgtctcagaaaaatcatggcaaa 5580

************************************************************

[Tet2]_Dnmt3a(+/+)_WT_CD8_Reference caggaaaagcgtgagcccacagggccacaggaacccagttacctgcgtttcatccagtct 5640

[Tet2]_Dnmt3a(+/-)_PTCL_1_(AF(X)s8) caggaaaagcgtgagcccacagggccacaggaacccagttacctgcgtttcatccagtct 5640

[Tet2]_Dnmt3a(+/-)_PTCL_2_(AF%(XI)x2) caggaaaagcgtgagcccacagggccacaggaacccagttacctgcgtttcatccagtct 5640

[Tet2]_Dnmt3a(-/-)_PTCL_1_(AF(IX)d4) caggaaaagcgtgagcccacagggccacaggaacccagttacctgcgtttcatccagtct 5640

[Tet2]_Dnmt3a(-/-)_PTCL_2_(AF(IX)i4) caggaaaagcgtgagcccacagggccacaggaacccagttacctgcgtttcatccagtct 5640

************************************************************

[Tet2]_Dnmt3a(+/+)_WT_CD8_Reference cttgctgagaacacagggtctgtgactacggattctaccgtgactacatcaccatatgct 5700

[Tet2]_Dnmt3a(+/-)_PTCL_1_(AF(X)s8) cttgctgagaacacagggtctgtgactacggattctaccgtgactacatcaccatatgct 5700

[Tet2]_Dnmt3a(+/-)_PTCL_2_(AF%(XI)x2) cttgctgagaacacagggtctgtgactacggattctaccgtgactacatcaccatatgct 5700

[Tet2]_Dnmt3a(-/-)_PTCL_1_(AF(IX)d4) cttgctgagaacacagggtctgtgactacggattctaccgtgactacatcaccatatgct 5700

[Tet2]_Dnmt3a(-/-)_PTCL_2_(AF(IX)i4) cttgctgagaacacagggtctgtgactacggattctaccgtgactacatcaccatatgct 5700

************************************************************

[Tet2]_Dnmt3a(+/+)_WT_CD8_Reference ttcactcaggtcacagggccttacaacacatttgtatga 5739

[Tet2]_Dnmt3a(+/-)_PTCL_1_(AF(X)s8) ttcactcaggtcacagggccttacaacacatttgtatga 5739

[Tet2]_Dnmt3a(+/-)_PTCL_2_(AF%(XI)x2) ttcactcaggtcacagggccttacaacacatttgtatga 5739

[Tet2]_Dnmt3a(-/-)_PTCL_1_(AF(IX)d4) ttcactcaggtcacagggccttacaacacatttgtatga 5739

[Tet2]_Dnmt3a(-/-)_PTCL_2_(AF(IX)i4) ttcactcaggtcacagggccttacaacacatttgtatga 5739

***************************************
